# Thirty-one new species of the spider genus *Leclercera* from Southeast Asia (Araneae, Psilodercidae)

**DOI:** 10.3897/zookeys.913.48650

**Published:** 2020-02-19

**Authors:** Wan-Jin Chang, Shuqiang Li

**Affiliations:** 1 Institute of Zoology, Chinese Academy of Sciences, Beijing 100101, China Institute of Zoology, Chinese Academy of Sciences Beijing China

**Keywords:** Chelicerae, copulatory organs, endemic, Ochyroceratidae, taxonomy, tropical Asia

## Abstract

Thirty-one new species of the genus *Leclercera* Deeleman-Reinhold, 1995 from China, Indonesia, Malaysia, Myanmar, Nepal, and Thailand are described: *L. mianqiu***sp. nov.** (♂♀), *L. thamsangensis***sp. nov.** (♂♀), *L. yandou***sp. nov.** (♂♀), *L. thamkaewensis***sp. nov.** (♂♀), *L. xiangbabang***sp. nov.** (♂♀), *L. jianzuiyu***sp. nov.** (♂♀), *L. yamaensis***sp. nov.** (♂♀), *L. banensis***sp. nov.** (♂♀), *L. dumuzhou***sp. nov.** (♀), *L. suwanensis***sp. nov.** (♂♀), *L. maochong***sp. nov.** (♀), *L. shanzi***sp. nov.** (♀), *L. duandai***sp. nov.** (♂♀), *L. hponensis***sp. nov.** (♂♀), *L. lizi***sp. nov.** (♂), *L. xiaodai***sp. nov.** (♀), *L. yanjing***sp. nov.** (♀), *L. ekteenensis***sp. nov.** (♂), *L. zhamensis***sp. nov.** (♂), *L. sanjiao***sp. nov.** (♀), *L. selasihensis***sp. nov.** (♂♀), *L. paiensis***sp. nov.** (♀), *L. yuanzhui***sp. nov.** (♀), *L. zanggaensis***sp. nov.** (♀), *L. aniensis***sp. nov.** (♂♀), *L. renqinensis***sp. nov.** (♂♀), *L. shergylaensis***sp. nov.** (♂♀), *L. pulongensis***sp. nov.** (♂), *L. tudao***sp. nov.** (♂♀), *L. duibaensis***sp. nov.** (♂), and *L. jiazhongensis***sp. nov.** (♂♀). Types are deposited in the Institute of Zoology, Chinese Academy of Sciences (IZCAS) in Beijing.

## Introduction

The spider family Psilodercidae Machado, 1951 was previously a subfamily of Ochyroceratidae Fage, 1912 until Wunderlich (2004) elevated it to the family level. To date, Psilodercidae includes a total of 165 species in 11 genera (WSC 2019), and the family has been shown to be monophyletic (Li and Li 2018). It is distributed in Southeast Asia, southern China, and parts of South Asia (WSC 2019, Li et al. 2020). The number of species has ballooned to almost three times its size in the 21^st^ century, from only 53 species known by the end of 20^th^ century (Platnick 2000). More than half of the psilodercid genera, including *Flexicrurum* Tong & Li, 2007, *Luzonacera* Li & Li, 2017, *Priscaleclercera* Wunderlich, 2017, *Qiongocera* Li & Li, 2017, *Relictocera* Li & Li, 2017, *Sinoderces* Li & Li, 2017, and *Thaiderces* Li & Li, 2017, have been described only recently.

The genus *Leclercera* Deeleman-Reinhold, 1995 was placed in the subfamily Psilodercinae in the family Ochyroceratidae Fage, 1912 before the subfamily was elevated to family rank. A total of 11 species of the genus have been described so far (WSC 2019). More than half of these are found in Nepal (*Leclercera machadoi* (Brignoli, 1973), *L. mulcata* (Brignoli, 1973), *L. nagarjunensis* Li & Li, 2018, *L. niuqu* Li & Li, 2018, *L. sidai* Li & Li, 2018, *L. zhaoi* Li & Li, 2018), and the rest are distributed in Borneo (*L. ocellata* Deeleman-Reinhold, 1995), China (*L. undulata* Wang & Li, 2013), the Philippines (*L. negros* Deeleman-Reinhold, 1995), and Thailand (*L. khaoyai* Deeleman-Reinhold, 1995, *L. longiventris* Deeleman-Reinhold, 1995).

While examining spider collections from tropical Asia, we found 31 new species of *Leclercera* from China, Indonesia, Malaysia, Myanmar, Nepal, and Thailand. The goal of this paper is to provide detailed descriptions of these new species with images of their copulatory organs and chelicerae.

## Materials and methods

Types are deposited in the Institute of Zoology, Chinese Academy of Sciences (IZCAS) in Beijing. All specimens collected were studied and preserved in 95% ethanol. The specimens were measured and examined with a Leica M205 C stereomicroscope, and further morphological details were observed with an Olympus BX41 compound microscope. Male palps were detached from the left side of the animal for further examination (except for *Leclercera xiangbabang* sp. nov. whose right palp was detached). Carapace length was measured excluding the clypeus. Internal genitalia of the female and palpal bulbs were dissected and immersed in lactic acid. An Olympus C7070 wide zoom digital camera (7.1 megapixels) mounted on an Olympus SZX12 stereomicroscope was used to take photos at different focal planes. The photos were then transferred to the image stacking software Helicon Focus 6.7.1 to generate photos with a greater depth of field before further processing with Adobe Photoshop CC 2014. Leg measurements are shown as total length: femur, patella, tibia, metatarsus, and tarsus. Leg segments were measured from their retrolateral side. All measurements are given in millimetres (mm). All terminology follows that of Li et al. (2014).

## Taxonomy

### Family Psilodercidae Machado, 1951

#### Genus Leclercera

Taxon classificationAnimaliaAraneaePsilodercidae

Deeleman-Reinhold, 1995

C50052ED-1216-5D6F-A642-4BA298D0CFE1

##### Type species.

*Leclercera khaoyai* Deeleman-Reinhold, 1995 from Thailand.

##### Emended diagnosis.

*Leclercera* resembles *Luzonacera* Li & Li, 2017 by having a shallow fovea, a slanted clypeus and labium, cheliceral lamina with two triangular extensions, and one promarginal cheliceral tooth and two retromarginal cheliceral teeth. However, they can be differentiated by the following combination of characters: 1) absence of a cymbial protrusion (vs. presence of a cymbial protrusion); 2) presence or absence of a conductor (vs. absence of a conductor); 3) palp with a retrolateral apophysis on tibia or cymbium (vs. palp without a retrolateral apophysis); 4) a non-pyriform bulb (vs. a pyriform bulb); and 5) different forms of spermathecae, with only one pair of stalked spermathecae.

##### Composition.

*Leclercera khaoyai* Deeleman-Reinhold, 1995 (♂♀) (the type species), *L. longiventris* Deeleman-Reinhold, 1995 (♂), *L. machadoi* (Brignoli, 1973) (♂♀), *L. mulcata* (Brignoli, 1973) (♀), *L. nagarjunensis* Li & Li, 2018 (♂♀), *L. negros* Deeleman-Reinhold, 1995 (♀), *L. niuqu* Li & Li, 2018 (♂), *L. ocellata* Deeleman-Reinhold, 1995 (♀), *L. sidai* Li & Li, 2018 (♂♀), *L. undulata* Wang & Li, 2013 (♂♀), *L. zhaoi* Li & Li, 2018 (♂♀), *L. mianqiu* sp. nov. (♂♀), *L. thamsangensis* sp. nov. (♂♀), *L. yandou* sp. nov. (♂♀), *L. thamkaewensis* sp. nov. (♂♀), *L. xiangbabang* sp. nov. (♂♀), *L. jianzuiyu* sp. nov. (♂♀), *L. yamaensis* sp. nov. (♂♀), *L. banensis* sp. nov. (♂♀), *L. dumuzhou* sp. nov. (♀), *L. suwanensis* sp. nov. (♂♀), *L. maochong* sp. nov. (♀), *L. shanzi* sp. nov. (♀), *L. duandai* sp. nov. (♂♀), *L. hponensis* sp. nov. (♂♀), *L. lizi* sp. nov. (♂), *L. xiaodai* sp. nov. (♀), *L. yanjing* sp. nov. (♀), *L. ekteenensis* sp. nov. (♂), *L. zhamensis* sp. nov. (♂), *L. sanjiao* sp. nov. (♀), *L. selasihensis* sp. nov. (♂♀), *L. paiensis* sp. nov. (♀), *L. yuanzhui* sp. nov. (♀), *L. zanggaensis* sp. nov. (♀), *L. aniensis* sp. nov. (♂♀), *L. renqinensis* sp. nov. (♂♀), *L. shergylaensis* sp. nov. (♂♀), *L. pulongensis* sp. nov. (♂), *L. tudao* sp. nov. (♂♀), *L. duibaensis* sp. nov. (♂), and *L. jiazhongensis* sp. nov. (♂♀)

##### Distribution.

The genus is known from China to Philippines and south to Malaysia and Indonesia.

#### Leclercera mianqiu sp. nov.

Taxon classificationAnimaliaAraneaePsilodercidae

17DD29CA-0A6D-5A2B-97D2-351F195ECFA3

http://zoobank.org/4A9A07DD-1443-4EC7-8C72-3A9796CFB242

**Figure 1.**
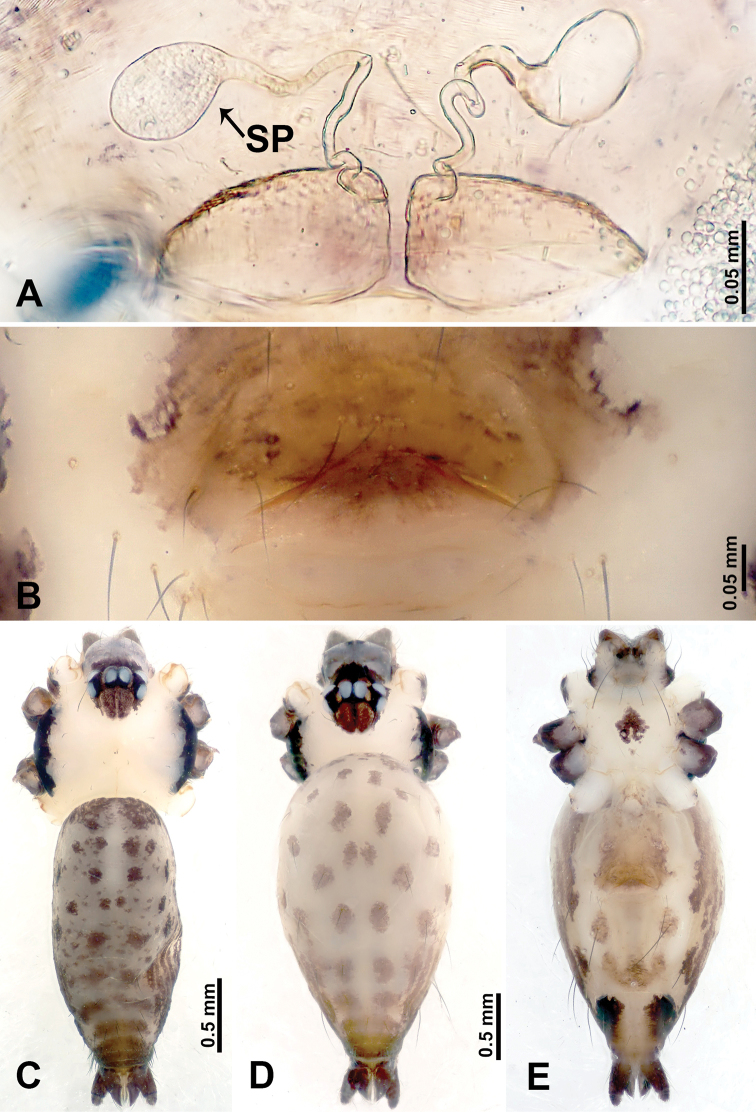
*Leclercera mianqiu* sp. nov., male holotype and female paratype. **A** Endogyne, dorsal view **B** female epigastric area, ventral view **C** male habitus, dorsal view **D** female habitus, dorsal view **E** female habitus, ventral view. Abbreviation: SP = spermatheca.

**Figure 2.**
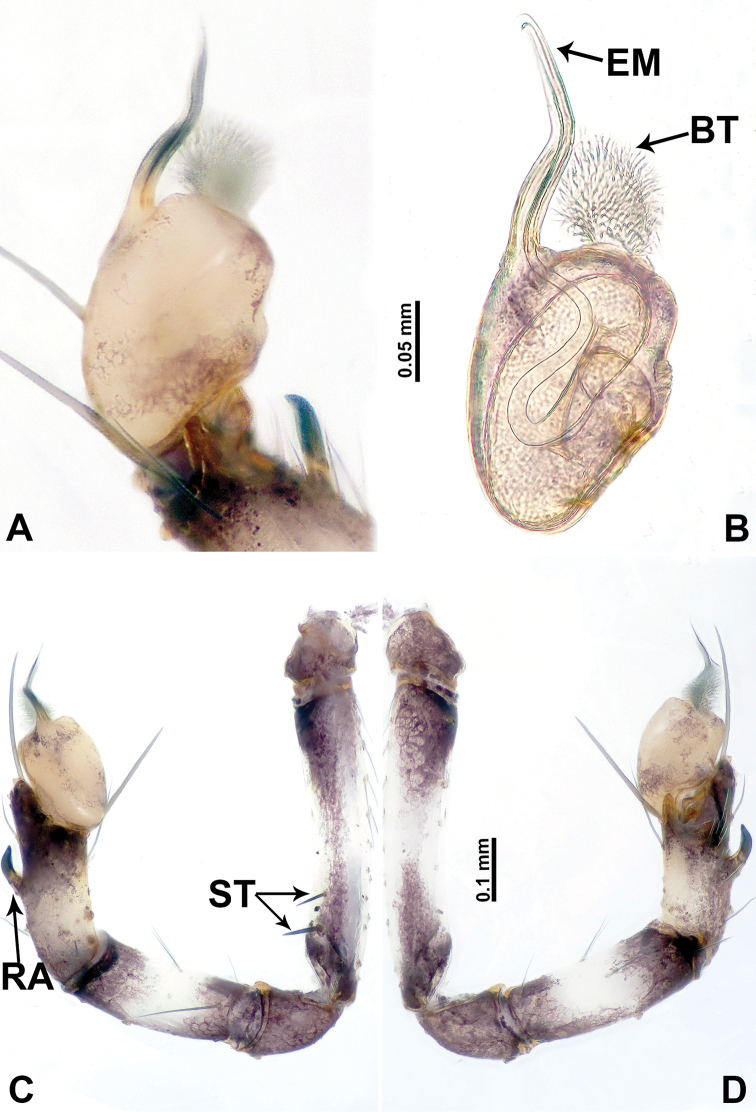
*Leclercera mianqiu* sp. nov., male holotype. **A** Palp, ventral view **B** bulb, ventral view **C** palp, prolateral view **D** palp, retrolateral view. Abbreviations: BT = bristle, EM = embolus, RA = retrolateral apophysis, ST = strong setae.

Figs 1 , 2 , 56H , 58

##### Types.

***Holotype***: ♂ (IZCAS), Indonesia, Sulawesi, mountain in Palopo, 2°57.7790'S, 120°8.5230'E, elevation ca 370 m, 13.IX.2017, H. Liu and Z. Chen leg. ***Paratype***: 1♀ (IZCAS), same data as holotype.

##### Etymology.

The species name is a noun in apposition derived from the Chinese pinyin “miánqiú” (cotton ball) and refers to the unique fluffy ball of bristles on the bulb resembling a cotton ball (Fig. 2B).

##### Diagnosis.

Males of *L. mianqiu* sp. nov. resemble *L. selasihensis* sp. nov. but can be distinguished by the presence of an attached fluffy ball of bristles adjacent to the embolus (vs. an absence of bristles but the presence of a conductor adjacent to embolus), presence of a medial-retrolateral apophysis on the cymbium (Fig. 2C) (vs. the presence of a postero-retrolateral apophysis on the cymbium (Fig. 37D)), the presence of two strong prolateral setae on the femur (Fig. 2C) (vs. the presence of one strong prolateral seta on the femur (Fig. 37D)); females can be distinguished by their rather pale coloration (vs. dark coloration), their stalked spermathecae that are globose distally (Fig. 1A) (vs. bean-shaped spermathecae that are strongly depressed anteriorly, and with median spiralled ducts (Fig. 36A)).

##### Description.

**Male** (Holotype). Total length 2.60; carapace 1.00 long, 1.00 wide; abdomen 1.60 long, 0.80 wide. Carapace round and pale yellow, margin with black bands laterally (Fig. 1C). Chelicerae dark brown (Fig. 56H). Clypeus greyish brown. Endites pale yellow with dark edges. Labium light brown. Sternum pale yellow, with dark brown spots centrally. Abdomen elongated, dorsum with complex dark brown sports, antero-ventrally with rectangular brown patch, posterior half pale yellow with dark and light brown spots. Legs uniformly brown; measurements: I 31.16 (7.69, 0.40, 8.01, 12.82, 2.24), II 19.21 (5.45, 0.40, 5.13, 6.73, 1.50), III 14.90 (4.25, 0.40, 4.25, 4.75, 1.25), IV 20.97 (6.41, 0.40, 6.09, 6.47, 1.60). Palp (Fig. 2A–D): femur slender, four times longer than patella, anteriorly with two strong setae prolaterally, dark purplish proximally and distally; patella not swollen, dark purplish; tibia 1.5 times shorter than femur, dark purplish proximally and distally; cymbium two times shorter than femur, dark purplish distally, with broad and slightly curved medio-retrolateral apophysis; bulb light brown, obovoid, with embolus and a clump of bristles distally; embolus slightly curved, as long as tegulum, adjacent to bristles; bristles forming a rounded fluffy clump anteriorly, adjacent to embolus (Fig. 2B).

**Female** (Paratype). General features and coloration similar to those of male (Fig. 1D, E). Measurements: total length 2.76; carapace 0.88 long, 0.86 wide; abdomen 1.88 long, 1.00 wide. Leg measurements: I 20.59 (5.13, 0.40, 5.45, 7.69, 1.92), II 12.25 (3.25, 0.40, 3.40, 4.00, 1.20), III missing, IV 14.49 (4.00, 0.40, 4.00, 4.81, 1.28). Epigastric area (Fig. 1B): an elliptical patch with distinct dark brown lines anteriorly and posteriorly, posterior pale yellow. Endogyne (Fig. 1A): stalked spermathecae, globose distally and with a pair of deltoid-shaped receptacles, spermathecal heads almost five times wider than the stalks, stalks three times longer than heads.

##### Distribution.

Known only from the type locality (Fig. 58).

#### Leclercera thamsangensis sp. nov.

Taxon classificationAnimaliaAraneaePsilodercidae

E38B63EB-717F-5ADD-8DC2-A322FB21927D

http://zoobank.org/0A664B6A-03A5-4A15-BBEE-06D754270C97

**Figure 3.**
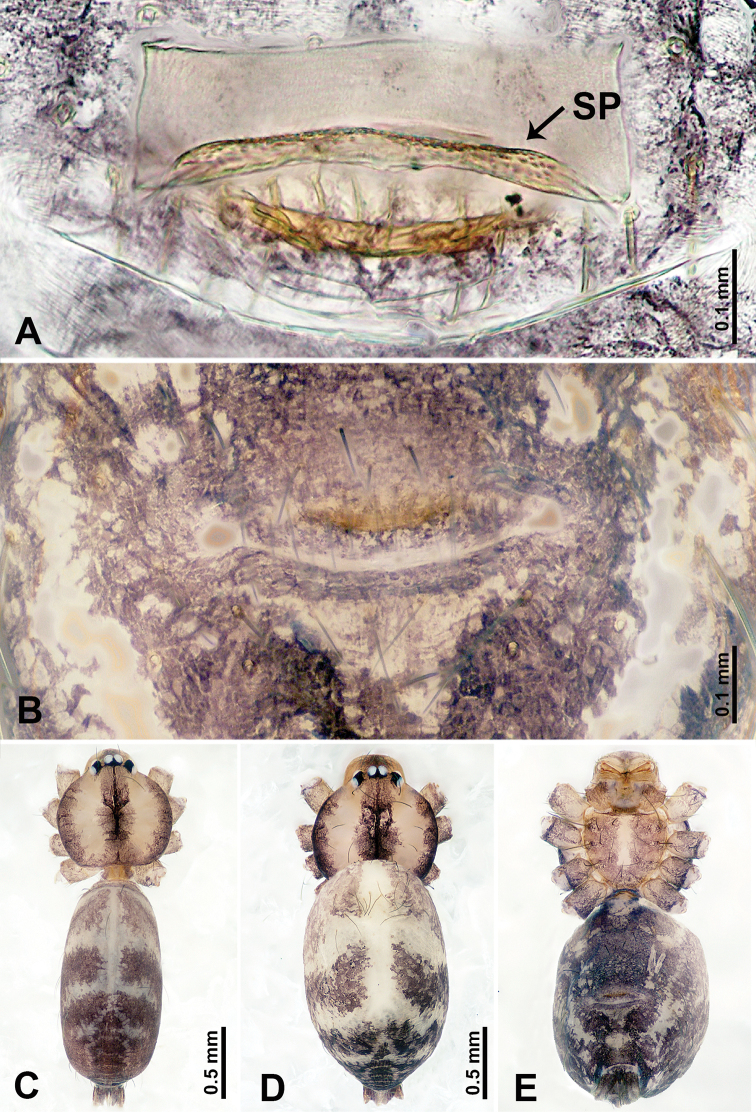
*Leclercera thamsangensis* sp. nov., male holotype and female paratype. **A** Endogyne, dorsal view **B** female epigastric area, ventral view **C** male habitus, dorsal view **D** female habitus, dorsal view **E** female habitus, ventral view. Abbreviation: SP = spermatheca.

**Figure 4.**
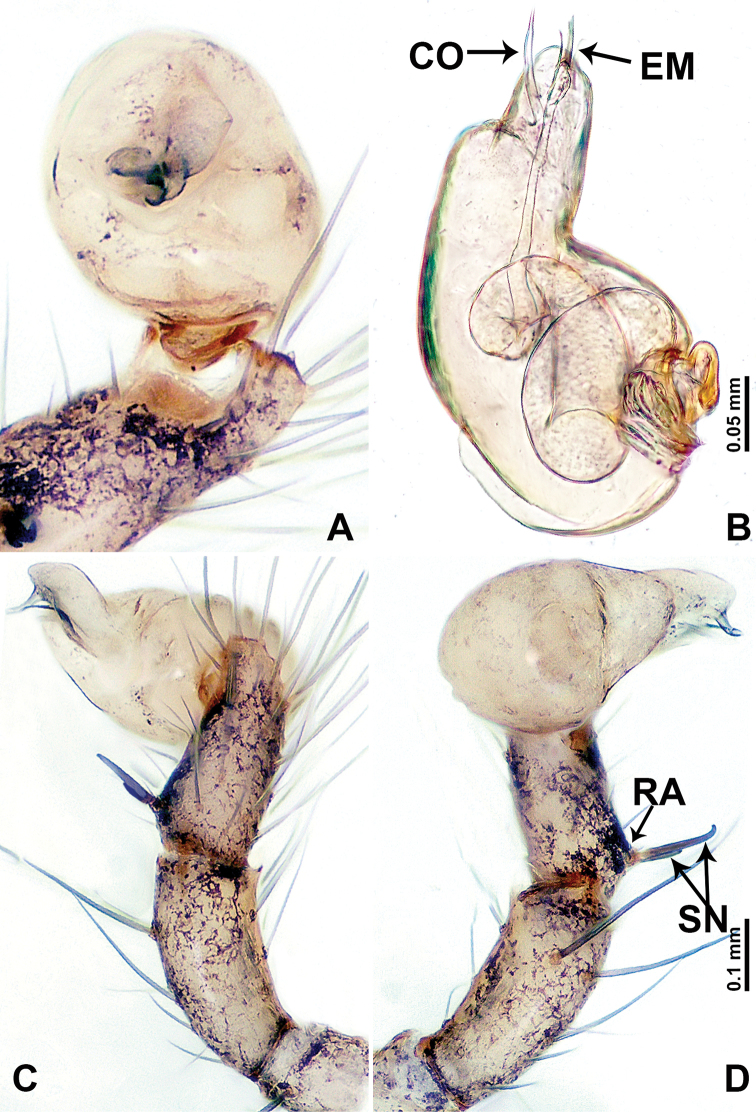
*Leclercera thamsangensis* sp. nov., male holotype. **A** Palp, ventral view **B** bulb, ventral view **C** palp, prolateral view **D** palp, retrolateral view. Abbreviations: CO = conductor, EM = embolus, RA = retrolateral apophysis, SN = spines.

Figs 3 , 4 , 56E , 58

##### Types.

***Holotype***: ♂ (IZCAS), Thailand, Loei Province, Phu Kradueng District, Phan Nok Kao Subdistrict, Tham Wat Phu Sang One, 16°49.0620'N, 101°56.4330'E, elevation ca 385 m, 29.XI.2016, H. Zhao leg. ***Paratype***: 1♀ (IZCAS), same data as holotype.

##### Etymology.

The species name is an adjective referring to the type locality.

##### Diagnosis.

Males of *L. thamsangensis* sp. nov. can be distinguished from congeners by the presence of two spines on a small retrolateral protrusion (retrolateral apophysis) of the cymbium (Fig. 4D) (vs. the absence of a retrolateral apophysis with two spines on the cymbium), the conductor and embolus are not widely separated, appearing to be similar in length and width (Fig. 4B) (vs. conductor and embolus different in congeners); females can be differentiated from congeners by rectangular, sheet-like spermathecae (Fig. 3A) (vs. absence of sheet-like spermathecae in congeners).

##### Description.

**Male** (Holotype). Total length 2.44; carapace 0.82 long, 0.84 wide; abdomen 1.62 long, 0.74 wide. Carapace round and brown, with three longitudinal dark brown bands, median band two times as wide as lateral band (Fig. 3C). Chelicerae pale brown (Fig. 56E). Clypeus brown. Endites brown. Labium dark brown basally. Sternum purplish, delimiting a light brown band medially. Abdomen elongated, dorsum with dark brown stripes laterally, delimiting a light brown band medially, antero-ventrally dark brown with elliptical patch, posterior part with indistinct dark and light brown pattern. Legs uniformly brown; measurements: I 9.76 (2.69, 0.38, 3.13, 2.53, 1.03), II 7.48 (2.09, 0.30, 2.34. 1.94, 0.81), III 5.56 (1.56, 0.30, 1.63, 1.41, 0.66), IV 8.74 (2.50, 0.30, 2.69. 2.31, 0.94). Palp (Fig. 4A–D): femur slender, 3.5 times longer than patella; patella not swollen; tibia 1.5 times shorter than femur; cymbium 2.5 times shorter than femur, with a small retrolateral apophysis bearing two spines basally, one spine half the length of the other; bulb light brown, pyriform, with embolus and conductor arising distally; embolus straight and thin, basally connected to conductor; conductor almost as long and wide as embolus (Fig. 4B).

**Female** (Paratype). General features and coloration similar to that of male (Fig. 3D, E). Measurements: total length 1.97; carapace 0.81 long, 0.84 wide; abdomen 1.16 long, 1.00 wide. Leg measurements: I 8.97 (2.44, 0.31, 2.91, 2.34, 0.97), II 6.72 (1.94, 0.31, 1.91, 1.78, 0.78), III 5.19 (1.41, 0.31, 1.50, 1.31, 0.66), IV 7.87 (2.19, 0.31, 2.50, 2.03, 0.84). Epigastric area (Fig. 3B): purplish crescent-shaped patch with a few setae, with random purplish patterns. Endogyne (Fig. 3A): pair of spermathecae slightly concave toward the posterior, bearing a rectangular sheet anteriorly.

##### Distribution.

Known only from the type locality (Fig. 58).

#### Leclercera yandou sp. nov.

Taxon classificationAnimaliaAraneaePsilodercidae

DB9DADC7-F5D9-5D41-9DD7-875B127C4B6B

http://zoobank.org/71776DDF-B11C-4F46-9650-9D56E92D76BC

**Figure 5.**
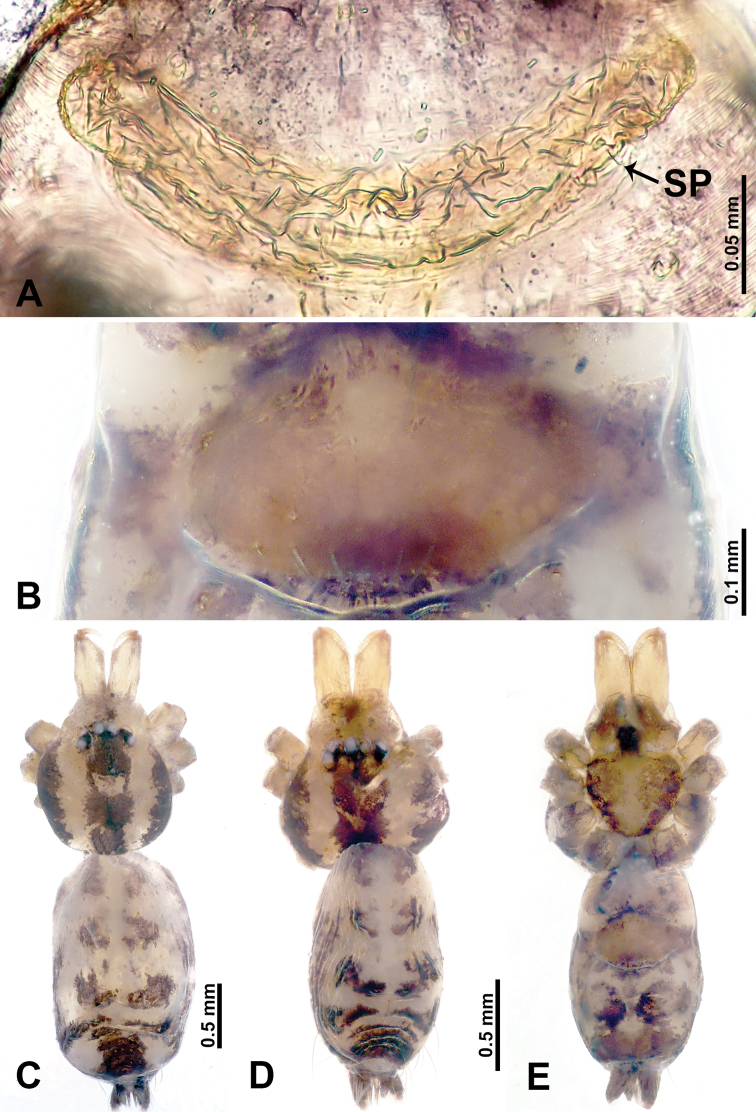
*Leclercera yandou* sp. nov., male holotype and female paratype. **A** Endogyne, dorsal view **B** female epigastric area, ventral view **C** male habitus, dorsal view **D** female habitus, dorsal view **E** female habitus, ventral view. Abbreviation: SP = spermatheca.

**Figure 6.**
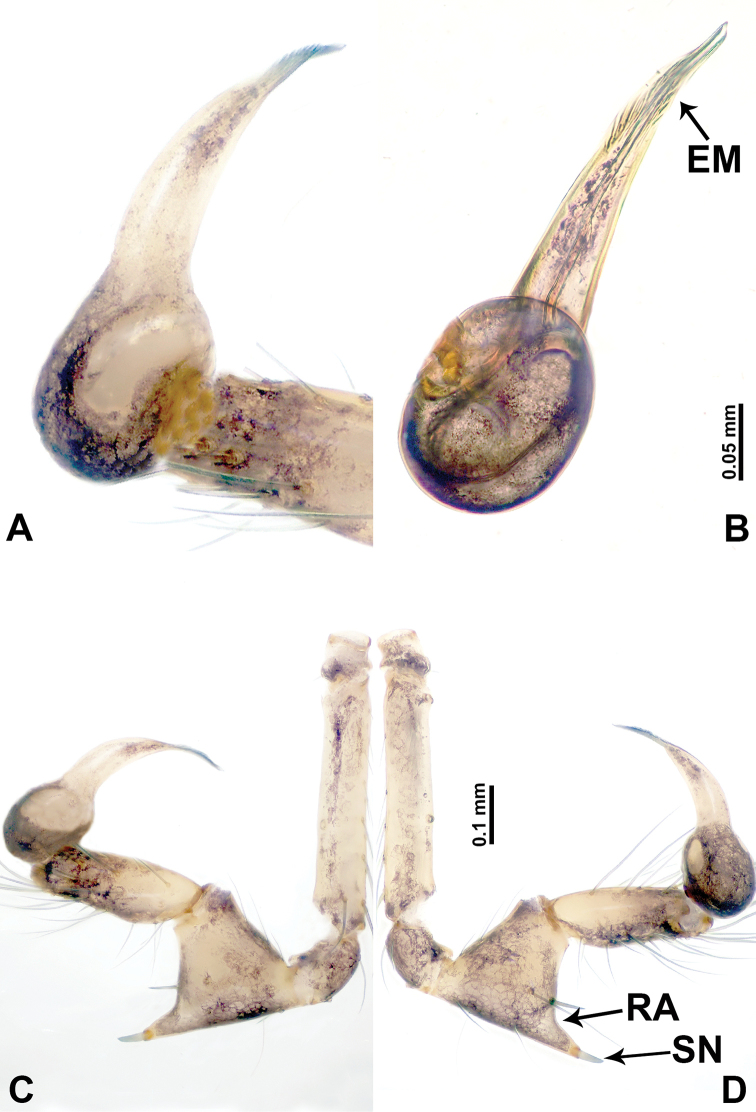
*Leclercera yandou* sp. nov., male holotype. **A** Palp, ventral view **B** bulb, ventral view **C** palp, prolateral view **D** palp, retrolateral view. Abbreviations: EM = embolus, RA = retrolateral apophysis, SN = spine.

Figs 5 , 6 , 55I , 58

##### Types.

***Holotype***: ♂ (IZCAS), Malaysia, Malay Peninsula, Pahang States, Fraser’s Hill, Telecom loop, Secondary Forest, 3°43.1050'N, 101°45.1643'E, elevation ca 1300 m, 17.II.2015, H. Zhao leg. ***Paratype***: 1♀ (IZCAS), same data as holotype.

##### Etymology.

The species name is a noun in apposition derived from the Chinese pinyin “yāndǒu” (smoking pipe) and refers to the palpal bulb which resembles a smoking pipe (Fig. 6B).

##### Diagnosis.

Males of *L. yandou* sp. nov. can be distinguished from congeners by the structure of the bulb, with a rounded base bearing a slightly curved and elongated embolus (Fig. 6B), the presence of a swollen triangular tibia with a retrolateral apophysis (Fig. 6D) (vs. absence of swollen triangular tibia in congeners); females can be differentiated from congeners by a pair of saucer-shaped, sinuous spermathecae (Fig. 5A)

##### Description.

**Male** (Holotype). Total length 2.50; carapace 0.75 long, 0.94 wide; abdomen 1.75 long, 0.80 wide. Carapace round and brown, with three longitudinal dark brown bands, median band twice as wide as lateral band (Fig. 5C). Chelicerae brown (Fig. 55I). Clypeus brown. Endites dark brown, light brown basally. Labium dark brown. Sternum brown, with dark brown patched laterally. Abdomen elongated, dorsum with 3 pairs of dark brown spots medially, median dark brown bands concentrated posteriorly, antero-ventrally brown with elliptical patch, posterior part with indistinct dark and light brown pattern. Legs uniformly brown; measurements: I 8.54 (2.81, 0.25, 2.97, 1.88, 0.63), II missing, III missing, IV 8.09 (2.40, 0.25, 2.19, 2.25, 1.00). Palp (Fig. 6A–D): femur slender, five times longer than patella; patella not swollen; tibia swollen, 1.5 times shorter than femur, forming a triangular shape with a retrolateral apophysis bearing a spine; cymbium two times shorter than femur, dark brown anteriorly; bulb spatulate with circular base, elongated embolus arises distally; embolus slightly bent, two times longer than the length of rounded tegulum and half the width of tegulum (Fig. 6B).

**Female** (Paratype). General features and coloration similar to those of male (Fig. 5D, E). Measurements: total length 2.10; carapace 0.80 long, 0.88 wide; abdomen 1.30 long, 0.70 wide. Leg measurements: I–III missing, IV 5.78 (1.63, 0.25, 1.60, 1.50, 0.80). Epigastric area (Fig. 5B): ovoid dark brown patch. Endogyne (Fig. 5A): a pair of sinuous spermathecae arching towards the anterior, ratio of width to length of entire spermathecae 1:7.

##### Distribution.

Known only from the type locality (Fig. 58).

#### Leclercera thamkaewensis sp. nov.

Taxon classificationAnimaliaAraneaePsilodercidae

DE21F989-4F15-5EBE-8529-B44F40E2F4A9

http://zoobank.org/5CB1D41A-D4C8-4056-B27C-3AD6A0284E4C

**Figure 7.**
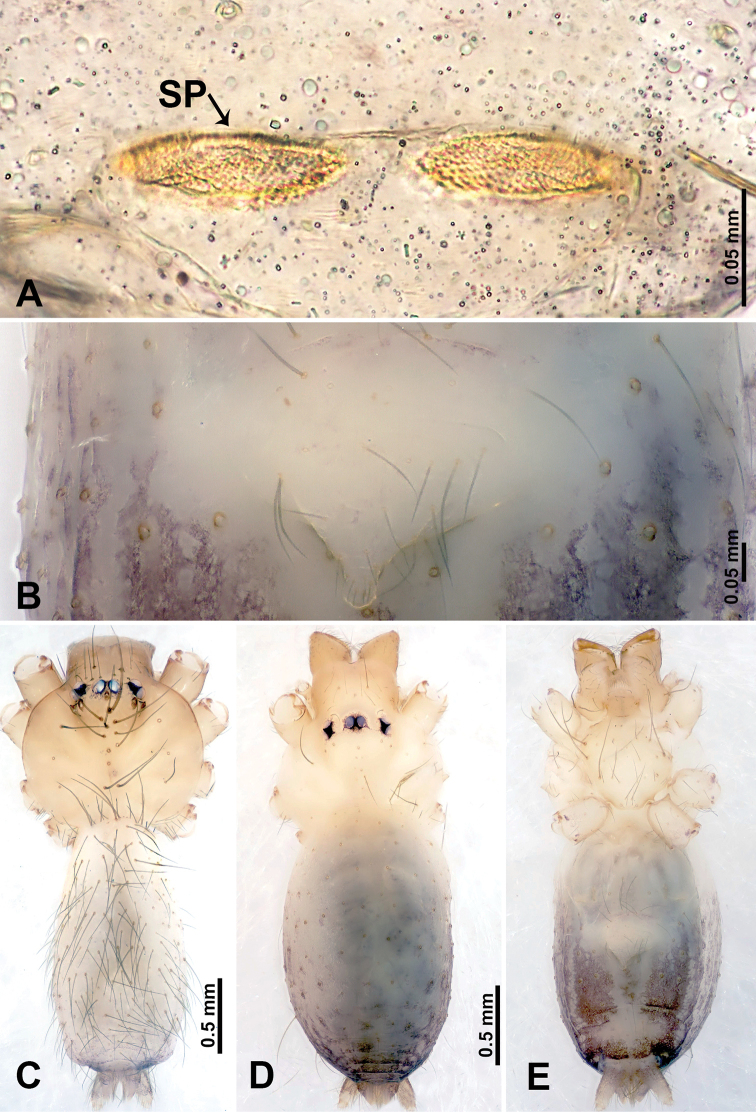
*Leclercera thamkaewensis* sp. nov., male holotype and female paratype. **A** Endogyne, dorsal view **B** female epigastric area, ventral view **C** male habitus, dorsal view **D** female habitus, dorsal view **E** female habitus, ventral view. Abbreviation: SP = spermatheca.

**Figure 8.**
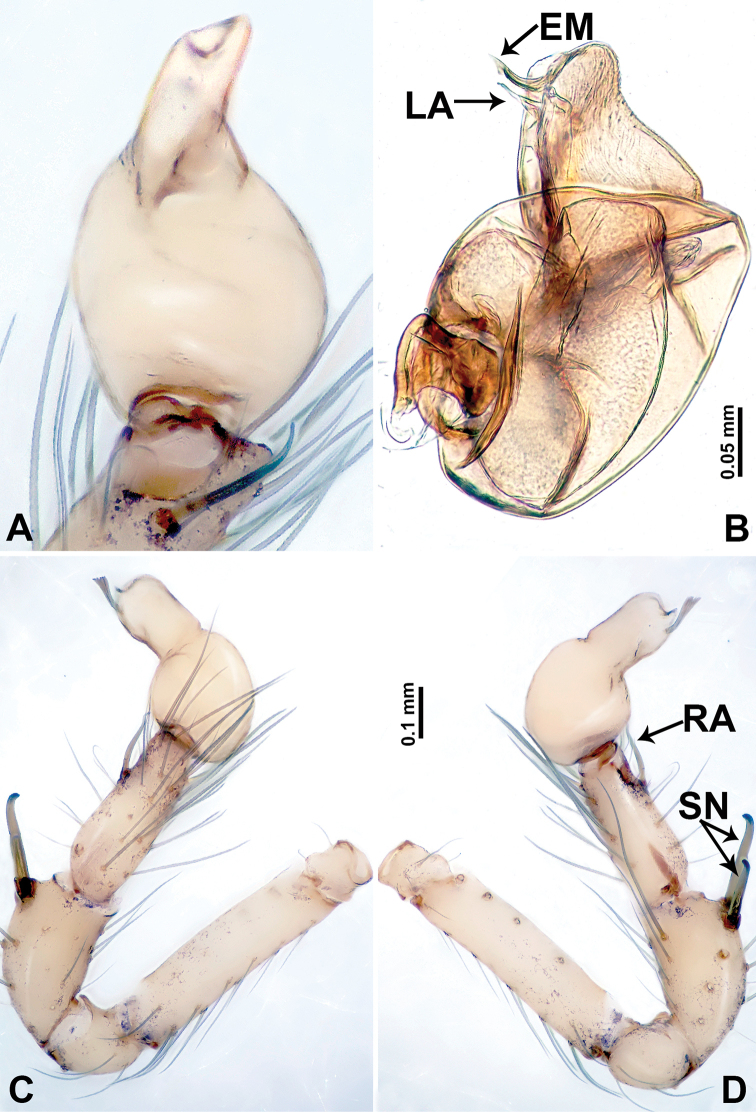
*Leclercera thamkaewensis* sp. nov. **A** Palp, ventral view **B** bulb, ventral view **C** palp, prolateral view **D** palp, retrolateral view. Abbreviations: EM = embolus, LA = laminar apophysis, RA = retrolateral apophysis, SN = spines.

Figs 7 , 8 , 56C , 58

##### Types.

***Holotype***: ♂ (IZCAS), Thailand, Sakaew Province, Klong Hat Subdistrict, Tham Phet Sai Kaew, 13°24.9620'N, 102°19.5890'E, elevation ca 243 m, 9.XI.2016, H. Zhao leg. ***Paratype***: 1♀ (IZCAS), same data as holotype.

##### Etymology.

The species name is an adjective referring to the type locality.

##### Diagnosis.

Males of *L. thamkaewensis* sp. nov. can be distinguished from congeners by the presence of a laminar apophysis adjacent to the embolus (vs. absence of a laminar apophysis, or if present, with more than one laminar apophyses or apophysis adheres to embolus in congeners), cymbium with fine retrolateral apophysis anteriorly, tibia swollen with retrolateral apophyses bearing two spines anteriorly (Fig. 8D) (vs. absence of such a combination of retrolateral apophyses in congeners); females can be differentiated from congeners by a pair of transverse, ovoid spermathecae (Fig. 7A).

##### Description.

**Male** (Holotype). Total length 2.50; carapace 0.90 long, 1.17 wide; abdomen 1.60 long, 0.86 wide. Carapace round and pale brown, with setae concentrated at ocular region (Fig. 7C). Chelicerae brown (Fig. 56C). Clypeus brown. Endites and labium pale brown. Sternum pale yellow, with sparse setae. Abdomen elongated, pale yellow, dorsum with dense setae, antero-ventrally pale yellow with inverted triangular genitalic lobe, dark brown posteriorly, defining pale yellow longitudinal band and a transverse band. Legs uniformly brown; measurements: I 12.90 (3.60, 0.40, 4.00, 3.50, 1.40), II 10.49 (3.00, 0.40, 3.25, 2.75, 1.09), III 8.08 (2.34, 0.40, 2.40, 2.00, 0.94), IV 11.40 (3.20, 0.40, 3.60, 3.00, 1.20). Palp (Fig. 8A–D): femur slender, four times longer than patella; patella not swollen; tibia swollen, 1.2 times shorter than femur, with retrolateral apophyses bearing two spines slightly bent at tip, one spine half the length of the other; cymbium 1.5 times shorter than femur, with a thin retrolateral apophysis anteriorly; bulb pyriform with embolus and laminar apophysis arising distally; embolus thin and sheet-like, widening toward tip; laminar apophysis shorter and thinner than embolus, adjacent to embolus (Fig. 8B).

**Female** (Paratype). General features and coloration similar to those of male (Fig. 7D, E). Measurements: total length 2.57; carapace 0.94 long, 0.94 wide; abdomen 1.63 long, 1.09 wide. Leg measurements: I 11.61 (3.21, 0.40, 3.60, 3.00, 1.40), II–III missing, IV 9.76 (2.80, 0.40, 2.97, 2.50, 1.09). Epigastric area (Fig. 7B): inverted triangle with rounded tip. Endogyne (Fig. 7A): pair of transverse ovoid spermathecae, width/length ratio of a spermatheca: 1:3.

##### Distribution.

Known only from the type locality (Fig. 58).

#### Leclercera xiangbabang sp. nov.

Taxon classificationAnimaliaAraneaePsilodercidae

60983BF1-245C-5E88-A340-7858EE638015

http://zoobank.org/63ED67F9-CF8B-4BD9-8579-66B1A8145341

**Figure 9.**
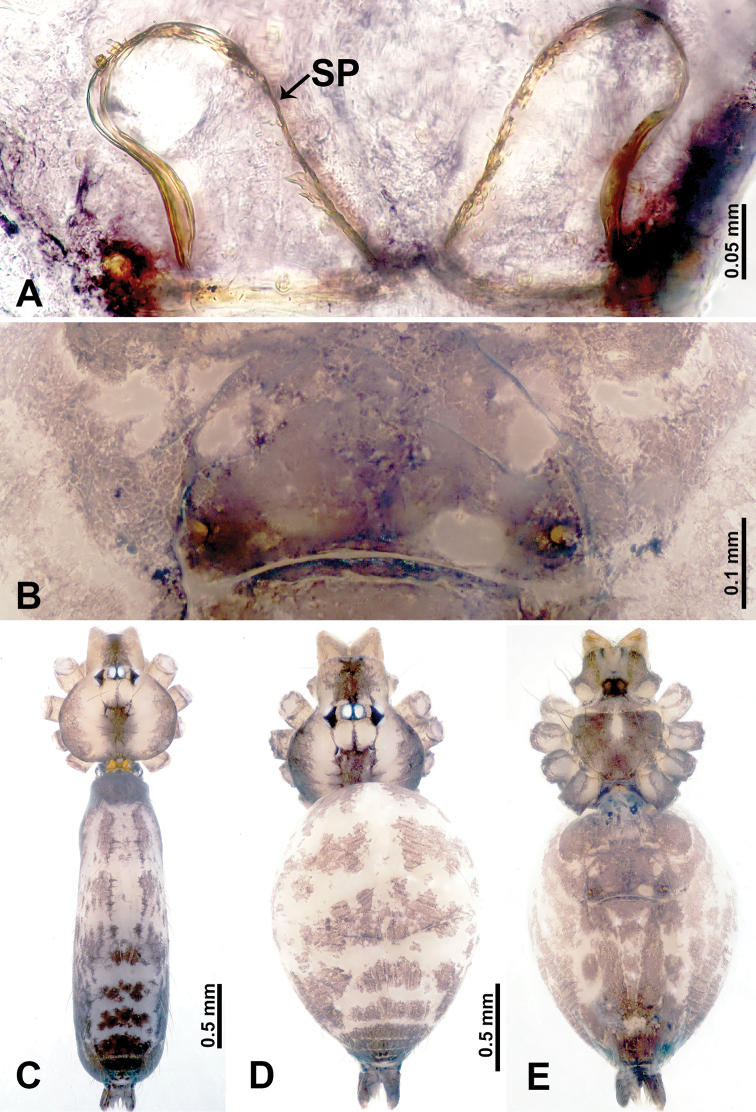
*Leclercera xiangbabang* sp. nov., male holotype and female paratype. **A** Endogyne, dorsal view **B** female epigastric area, ventral view **C** male habitus, dorsal view **D** female habitus, dorsal view **E** female habitus, ventral view. Abbreviation: SP = spermatheca.

**Figure 10.**
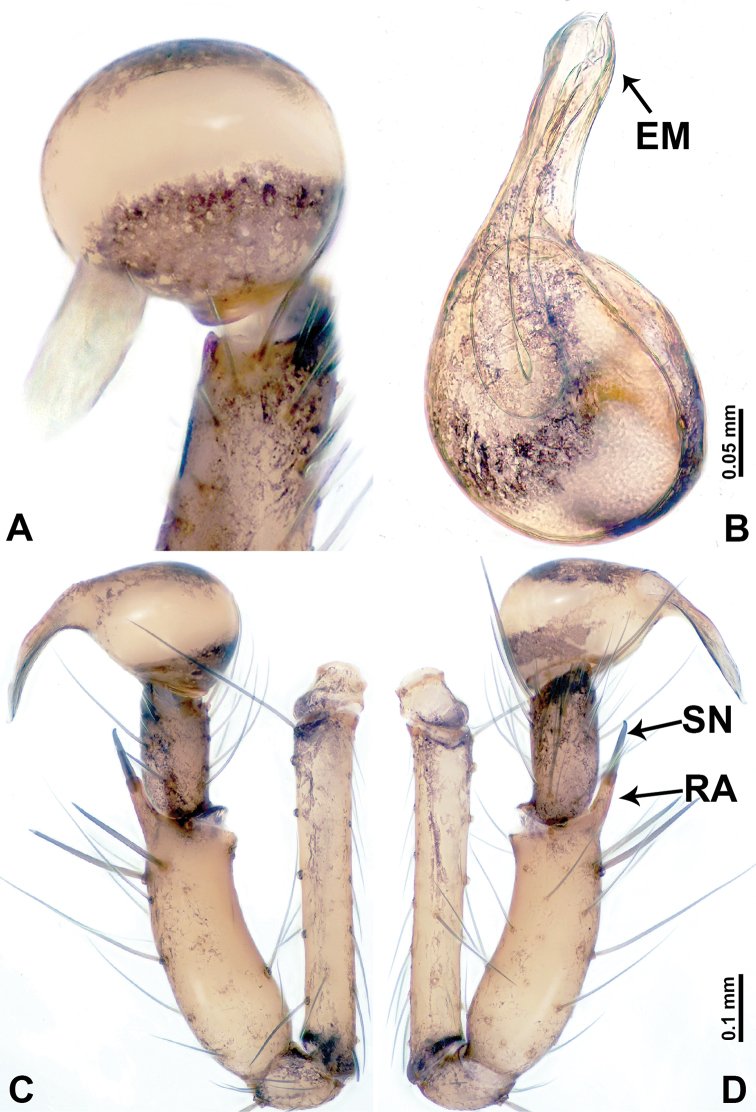
*Leclercera xiangbabang* sp. nov. **A** Palp, ventral view **B** bulb, ventral view **C** palp, prolateral view **D** palp, retrolateral view. Abbreviations: EM = embolus, RA = retrolateral apophysis, SN = spine.

Figs 9 , 10 , 55F , 58

##### Types.

***Holotype***: ♂ (IZCAS), Thailand, Kanchanaburi Province, Sai Yok District, Wang Krachae Subdistrict, Cave without name, 14°12.1820'N, 99°01.4161'E, elevation ca 342 m, 01.XI.2014, H. Zhao, Y. Li, Z. Chen leg. ***Paratype***: 1♀ (IZCAS), same data as holotype.

##### Etymology.

The species name is a noun in apposition derived from the Chinese pinyin “xiàngbábàng” (geoduck) and refers to the entire structure of the bulb which resembles the appearance of a Pacific geoduck bivalve.

##### Diagnosis.

**Figure 11.**
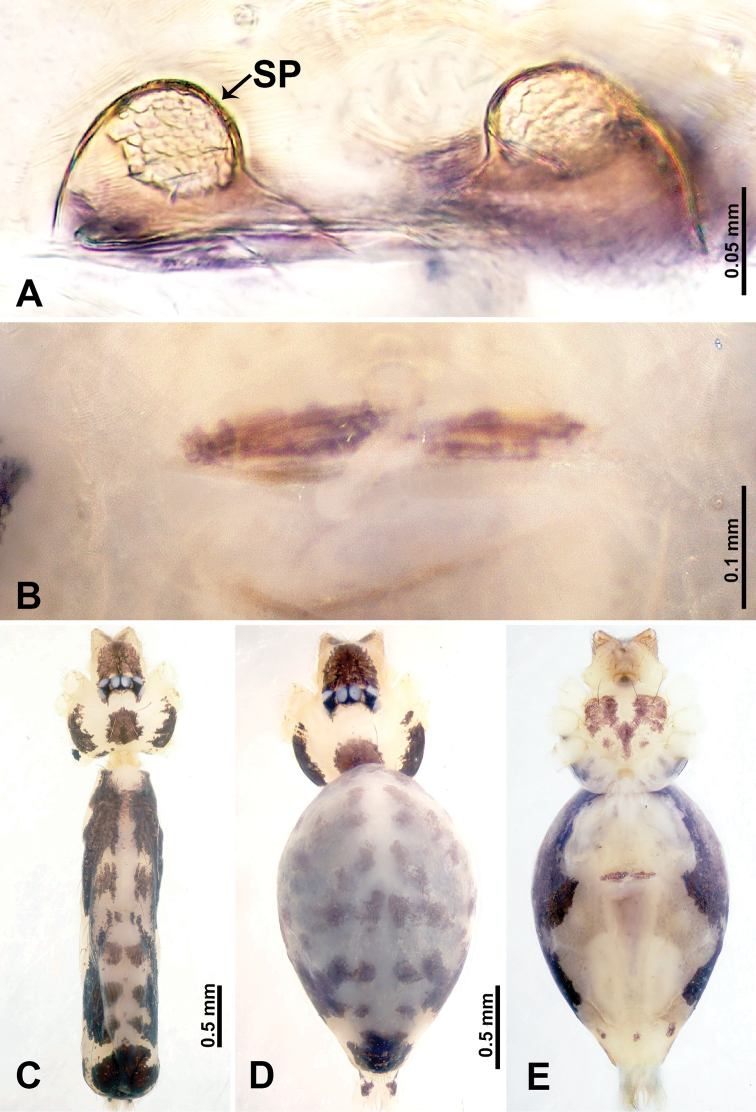
*Leclercera jianzuiyu* sp. nov., male holotype and female paratype. **A** Endogyne, dorsal view **B** female epigastric area, ventral view **C** male habitus, dorsal view **D** female habitus, dorsal view **E** female habitus, ventral view. Abbreviation: SP = spermatheca.

**Figure 12.**
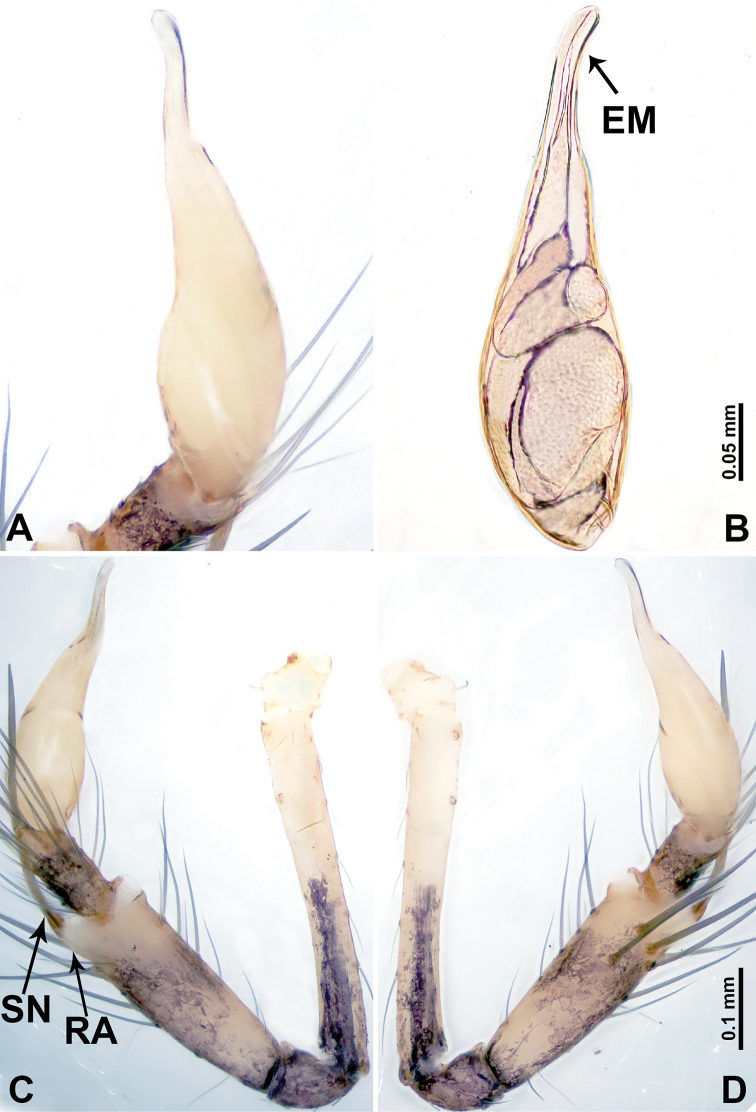
*Leclercera jianzuiyu* sp. nov. **A** Palp, ventral view **B** bulb, ventral view **C** palp, prolateral view **D** palp, retrolateral view. Abbreviations: EM = embolus, RA = retrolateral apophysis, SN = spine.

Males of *L. xiangbabang* sp. nov. resemble *L. jianzuiyu* sp. nov. by having a spatulate bulb and a retrolateral apophysis on the tibia but can be distinguished by a rather bulging bulb and a wider embolus (Fig. 10B) (vs. a rather slender bulb and thin embolus (Fig. 12B)), a retrolateral apophysis on tibia, half the length of the tegulum (Fig. 10D) (vs. retrolateral apophysis on tibia equal in length to tegulum (Fig. 12C)); females can be differentiated by the elongated tubular spermathecae (Fig. 9A) (vs. rounded spermathecae (Fig. 11A)).

##### Description.

**Male** (Holotype). Total length 3.18; carapace 0.80 long, 0.88 wide; abdomen 2.38 long, 0.80 wide. Carapace round and brown, with three longitudinal dark brown bands, median band two times wider than lateral band (Fig. 9C). Chelicerae brown (Fig. 55F). Clypeus dark brown medially, light brown laterally. Endites dark brown, light brown basally. Labium dark brown. Sternum dark brown, delimiting a short, light brown band medially. Abdomen elongated, anterior and posterior edge dark brown, with scattered dark and light brown patterns, antero-ventrally with dark brown circular patches laterally, with complex dark and light brown patterns posteriorly. Legs uniformly brown; measurements: I 12.75 (4.00, 0.25, 3.75, 3.00, 1.75), II 9.06 (2.75, 0.31, 2.40, 2.60, 1.00), III 6.51 (1.88, 0.25, 1.88, 1.75, 0.75), IV 9.51 (3.20, 0.31, 2.40, 2.60, 1.00). Palp (Fig. 10A–D): femur slender, four times longer than patella; patella not swollen; tibia swollen, 1.5 times shorter and two times wider than femur, with retrolateral apophysis anteriorly bearing a spine, about half the length of tegulum, spine and apophysis almost equal in length; cymbium dark brown, three times shorter than femur; bulb bulging, spatulate, brown, with embolus arising distally; embolus elongated, with blunt tip, as long as tegulum, tegulum three times wider than embolus (Fig. 10B).

**Female** (Paratype). General features and coloration similar to those of male (Fig. 9D, E). Measurements: total length 2.26; carapace 0.63 long, 0.75 wide; abdomen 1.63 long, 1.13 wide. Leg measurements: I missing, II 7.51 (2.25, 0.25, 2.13, 2.00, 0.88), III 7.81 (2.25, 0.25, 2.34, 2.03, 0.94), IV 5.30 (1.60, 0.25, 1.50, 1.25, 0.70). Epigastric area (Fig. 9B): dark brown slit that slightly curves posteriorly. Endogyne (Fig. 9A): elongated tubular spermathecae, slightly slanting, length of a spermatheca is 2.5 times its width.

##### Distribution.

Known only from the type locality (Fig. 58).

#### Leclercera jianzuiyu sp. nov.

Taxon classificationAnimaliaAraneaePsilodercidae

43548059-222E-5EF1-A49F-7B3CBA85CDA9

http://zoobank.org/8294145B-88E3-41DC-9E2F-8B0AB36F6A53

Figs 11 , 12 , 55E , 58

##### Types.

***Holotype***: ♂ (IZCAS), Thailand, Prachuap Kiri Khan Province, Hua Hin District, Nong Phlap Subdistrict, Laplae Cave and Kailone Cave, 12°36.2550'N, 99°43.3410'E, elevation ca 175 m, 30.X.2014, H. Zhao, Y. Li and Z. Chen leg. ***Paratype***: 1♀ (IZCAS), same data as holotype.

##### Etymology.

The species name is a noun in apposition derived from the Chinese pinyin “jiānzuĭyú” (bird wrasse – a type of fish) and refers to the entire structure of bulb which resembles the mouth of a bird wrasse.

##### Diagnosis.

Diagnostic features of males and females are discussed in the diagnosis of *L. xiangbabang* sp. nov.

##### Description.

**Male** (Holotype). Total length 2.88; carapace 0.63 long, 0.78 wide; abdomen 2.25 long, 0.63 wide. Carapace round and pale yellow, with three longitudinal dark brown bands, median band two times wider than lateral bands (Fig. 11C). Chelicerae brown (Fig. 55E). Clypeus dark brown medially, pale yellow laterally. Endites pale yellow. Labium brown, circular dark brown spot basally. Sternum pale yellow, anterior with dark brown patches laterally, posterior with dark brown band medially. Abdomen elongated, edges of anterior and posterior dark brown, with complex scattered dark brown pattern, antero-ventrally pale yellow with transverse brown band medially, black patches from edges of anterior to posterior. Legs uniformly brown; measurements: I 13.06 (3.75, 0.31, 4.00, 3.60, 1.40), II 8.63 (2.50, 0.25, 2.60, 2.34, 0.94), III missing, IV 9.20 (2.80, 0.20, 2.66, 2.60, 0.94). Palp (Fig. 12A–D): femur slender, five times longer than patella; patella not swollen; tibia swollen, 1.5 times shorter and two times wider than femur, with retrolateral apophysis anteriorly bearing a spine almost as long as tegulum; cymbium dark brown, 3.5 times shorter than femur; bulb thinly spatulate, pale brown, with embolus arising distally; embolus elongated with slightly bent, blunt tip, half the width of tegulum (Fig. 12B).

**Female** (Paratype). General features and coloration similar to those of male (Fig. 11E, F). Measurements: total length 2.00; carapace 0.60 long, 0.70 wide; abdomen 1.40 long, 1.25 wide. Leg measurements: I–II missing, III 4.45 (1.25, 0.20, 1.30, 1.20, 0.50), IV 7.20 (2.19, 0.25, 2.13, 1.88, 0.75). Epigastric area (Fig. 11B): a pair of dark brown horizontal patches laterally. Endogyne (Fig. 11A): a pair of circular spermathecae with thin strips laterally, wavy patterns in spermathecae, lateral stripes two times longer than circular spermathecae.

##### Distribution.

Known only from the type locality (Fig. 58).

#### Leclercera yamaensis sp. nov.

Taxon classificationAnimaliaAraneaePsilodercidae

FD0B3227-F381-5613-8FD8-24BABBC94764

http://zoobank.org/BDB65387-B1D6-4D78-A322-767846BF4E06

**Figure 13.**
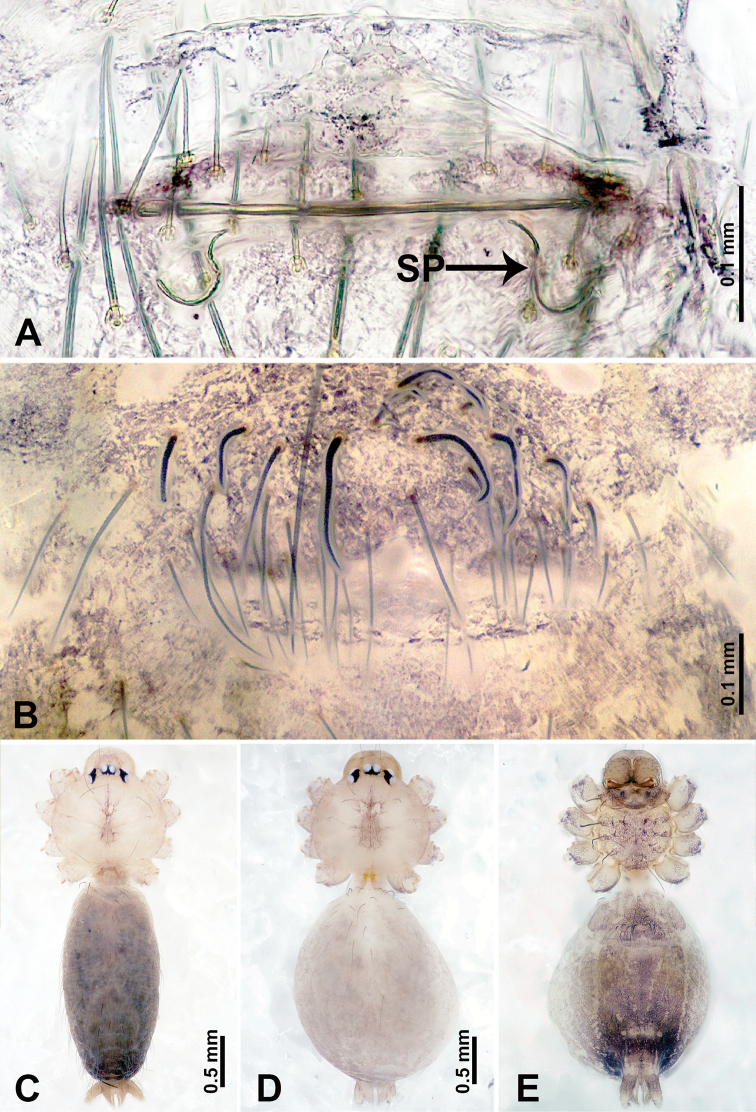
*Leclercera yamaensis* sp. nov., male holotype and female paratype. **A** Endogyne, dorsal view **B** female epigastric area, ventral view **C** male habitus, dorsal view **D** female habitus, dorsal view **E** female habitus, ventral view. Abbreviation: SP = spermatheca.

**Figure 14.**
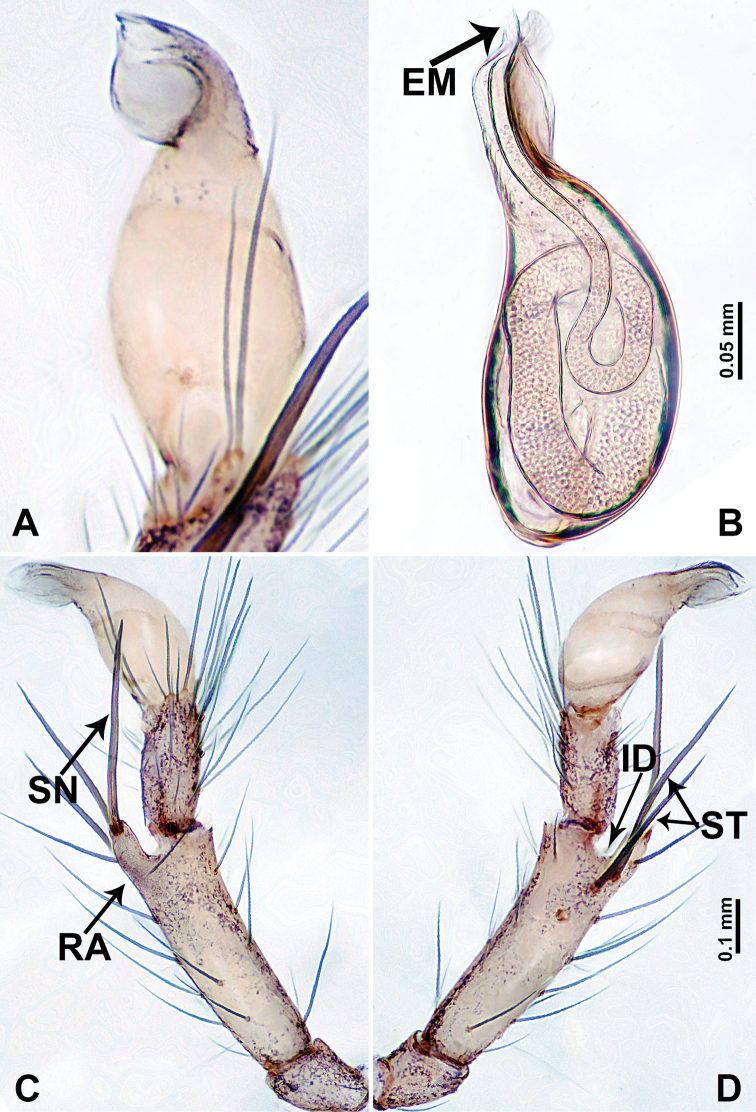
*Leclercera yamaensis* sp. nov. **A** Palp, ventral view **B** bulb, ventral view **C** palp, prolateral view **D** palp, retrolateral view. Abbreviations: EM = embolus, ID = indentation, RA = retrolateral apophysis, SN = spine, ST = strong setae.

Figs 13 , 14 , 56D , 58

##### Types.

***Holotype***: ♂ (IZCAS), Thailand, Tak Province, Umphang District, Umphang Subdistrict, Ya Mae Cave, 16°02.3530'N, 98°50.8120'E, elevation ca 454 m, 15.XI.2016, H. Zhao leg. ***Paratype***: 1♀ (IZCAS), same data as holotype.

##### Etymology.

The species name is an adjective referring to the type locality.

##### Diagnosis.

Males of *L. yamaensis* sp. nov. can be distinguished from congeners by an indentation on the anterior end of the palpal tibia formed by the presence of a retrolateral apophysis bearing a spine that is longer than the tegulum (Fig. 14D) (vs. the absence of such an indentation from a retrolateral apophysis in congeners), the presence of two strong setae on the retrolateral apophysis (Fig. 14D) (vs. the absence of setae on the retrolateral apophysis in congeners), the presence of a laminar apophysis adhering to the embolus (vs. absence of laminar apophysis, or if present, widely separated from the embolus in congeners); females can be differentiated from congeners by a pair of hook-like spermathecae (Fig. 13A).

##### Description.

**Male** (Holotype). Total length 2.62; carapace 0.82 long, 0.92 wide; abdomen 1.80 long, 0.80 wide. Carapace round and pale brown, with dark brown traces medially (Fig. 13C). Chelicerae brown (Fig. 56D). Clypeus pale brown. Endites and labium dark brown. Sternum with scattered purplish traces. Abdomen elongated, dorsum dark brown, antero-ventrally dark brown with triangular spot, posterior with dark brown, purplish pattern. Legs uniformly brown; measurements: I 13.91 (4.00, 0.31, 4.50, 3.75, 1.35), II 11.43 (3.32, 0.31, 3.60, 3.00, 1.20), III 8.22 (2.50, 0.31, 2.41, 2.03, 0.97), IV missing. Palp (Fig. 14A–D): femur slender, five times longer than patella; patella not swollen; tibia swollen, 1.5 times shorter and two times wider than femur, with retrolateral apophysis anteriorly bearing a spine longer than the length of the tegulum, apophysis forms an indentation on tibia anteriorly; cymbium 3.5 times shorter than femur; bulb pyriform, with embolus and laminar apophysis arising distally; embolus thin, with laminar apophysis attached; laminar apophysis five times shorter than the length of tegulum, adhering to embolus (Fig. 14B).

**Female** (Paratype). General features and coloration similar to those of male (Fig. 13E, F). Measurements: total length 2.50; carapace 0.80 long, 0.90 wide; abdomen 1.70 long, 1.20 wide. Leg measurements: I 12.45 (3.53, 0.31, 4.00, 3.33, 1.28), II 10.11 (2.84, 0.31, 3.20, 2.68, 1.08), III 6.99 (2.00, 0.31, 2.08, 1.76, 0.84), IV 10.15 (3.00, 0.31, 3.20, 2.60, 1.04). Epigastric area (Fig. 13B): brown slit surrounded with purplish spots. Endogyne (Fig. 13A): hook-like spermathecae, ratio of spermatheca width and spermatheca interdistance 1:4.5.

##### Distribution.

Known only from the type locality (Fig. 58).

#### Leclercera banensis sp. nov.

Taxon classificationAnimaliaAraneaePsilodercidae

622AF87C-B41F-5C90-B3E0-0812AB697758

http://zoobank.org/41B9DA08-475B-45BE-A8AC-6A23C903EAA5

**Figure 15.**
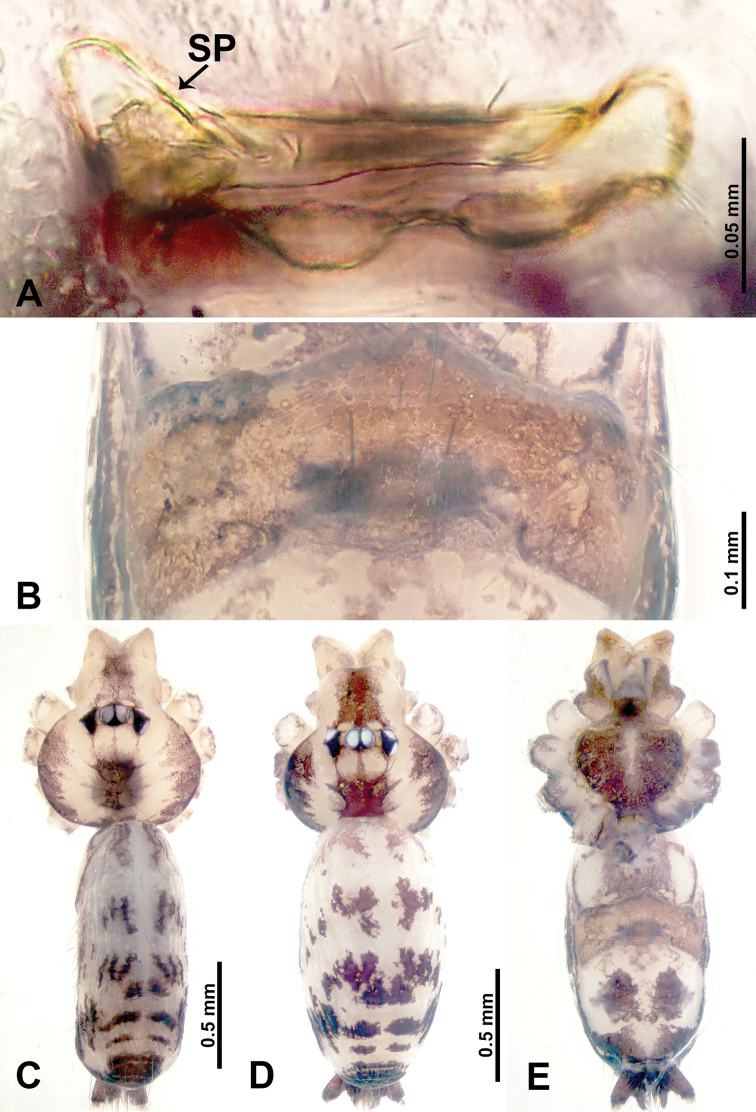
*Leclercera banensis* sp. nov., male holotype and female paratype. **A** Endogyne, dorsal view **B** female epigastric area, ventral view **C** male habitus, dorsal view **D** female habitus, dorsal view **E** female habitus, ventral view. Abbreviation: SP = spermatheca.

**Figure 16.**
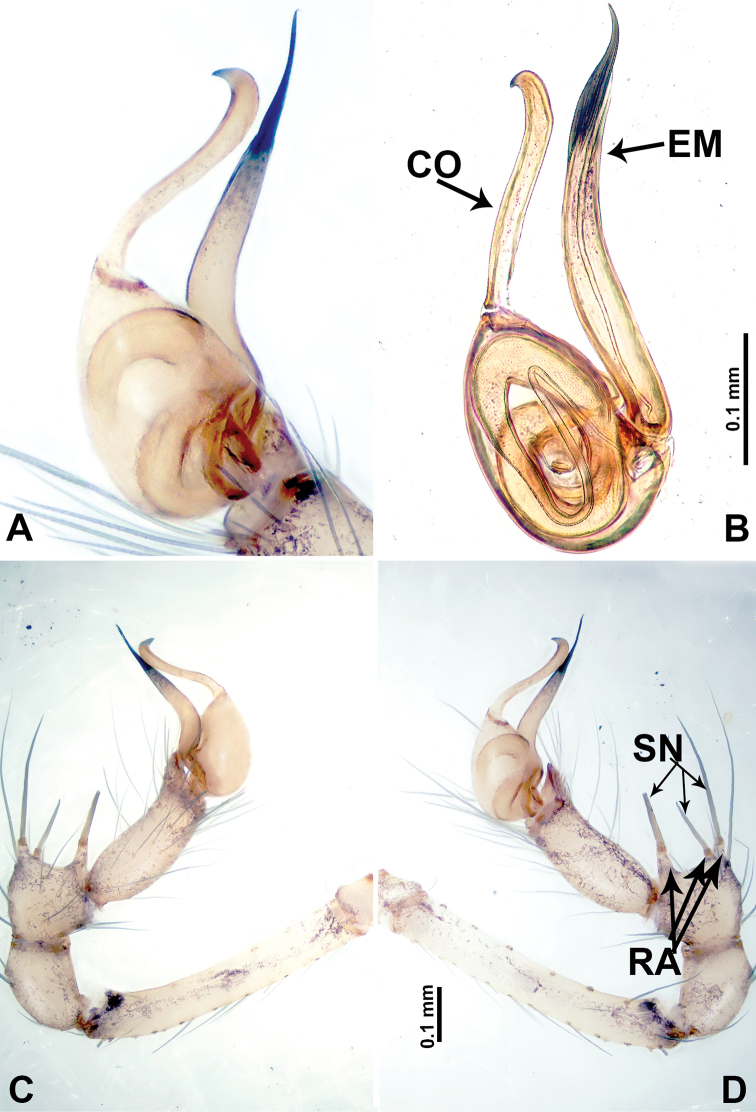
*Leclercera banensis* sp. nov. **A** Palp, ventral view **B** bulb, ventral view **C** palp, prolateral view **D** palp, retrolateral view. Abbreviations: CO = conductor, EM = embolus, RA = retrolateral apophyses, SN = spines.

Figs 15 , 16 , 55D , 58

##### Types.

***Holotype***: ♂ (IZCAS), Thailand, Krabi Province, Muang District, Ban Chong Plee Village, 8°5.1218'N, 98°51.2228'E, elevation ca 442 m, 25.X.2014, H. Zhao, Y. Li and Z. Chen leg. ***Paratype***: 1♀ (IZCAS), same data as holotype.

##### Etymology.

The species name is an adjective referring to the type locality.

##### Diagnosis.

Males of *L. banensis* sp. nov. resemble *L. suwanensis* sp. nov. but can be distinguished by an embolus that is longer and almost as wide as the conductor (Fig. 16B) (vs. an embolus that is equally as long as the conductor but almost four times wider basally (Fig. 19B)), bulb rather slender (vs. bulb rather expanded), patella swollen, cymbium not swollen (vs. patella not swollen, cymbium swollen), hexagonal swollen tibia (vs. pentagonal swollen tibia), tibia with three retrolateral apophyses, each bearing a spine (Fig. 16D) (vs. tibia with a single retrolateral apophysis bearing a spine and a strong seta (Fig. 19D)); females can be differentiated by a pair of flattened spermathecae with an anterior extension laterally (Fig. 15A) (vs. a pair of bulging, curled spermathecae with posterior extension laterally (Fig. 18A)).

##### Description.

**Male** (Holotype). Total length 1.83; carapace 0.63 long, 0.75 wide; abdomen 1.20 long, 0.70 wide. Carapace round and pale brown, with three longitudinal dark brown bands, median band two times wider than the lateral bands (Fig. 15C). Chelicerae brown (Fig. 55D). Clypeus dark brown medially, light brown laterally. Endites dark brown, light brown basally. Labium dark brown. Sternum dark brown with tiny light brown band medially. Abdomen elongated, dorsum with dark brown spots, antero-ventrally with brown horizontal band centrally, posterior with a pair of dark brown spots. Legs uniformly brown; measurements: I–II missing, III 5.21 (1.56, 0.25, 1.40, 1.40, 0.60), IV 7.85 (2.19, 0.25, 2.34, 2.19, 0.88). Palp (Fig. 16A–D): femur slender, 3 times longer than patella; patella swollen; tibia 3.5 times shorter than femur, hexagonally swollen, with three retrolateral apophyses anteriorly, each bearing a spine, with one spine longer than the other two; cymbium two times shorter than femur; bulb pale brown and ovoid, conductor and embolus separated, with conductor arising distally, embolus arising basally; conductor elongated and slightly hooked at tip, 2 times shorter than, but almost equally as wide as, embolus; embolus elongated, darkening distally (Fig. 16B).

**Female** (Paratype). General features and coloration similar to those of the male (Fig. 15D, E). Measurements: total length 1.80; carapace 0.60 long, 0.70 wide; abdomen 1.20 long, 0.70 wide. Leg measurements: I 7.97 (2.40, 0.25, 2.66, 2.03, 0.63), II 6.64 (1.88, 0.25, 1.88, 1.88, 0.75), III 4.15 (1.30, 0.20, 1.00, 1.10, 0.55), IV 6.28 (1.88, 0.20, 1.70, 1.75, 0.75). Epigastric area (Fig. 15B): an elliptical dark brown patch surrounded with a brown horizontal band. Endogyne (Fig. 15A): a pair of flattened spermathecae with tubular extensions laterally, and a pair of ovoid ducts posteriorly.

##### Distribution.

Known only from the type locality (Fig. 58).

#### Leclercera dumuzhou sp. nov.

Taxon classificationAnimaliaAraneaePsilodercidae

B54C15F5-DC03-5074-9C10-87A57147C765

http://zoobank.org/30CC9F7A-D769-46DC-9EBE-6BFE69D9A1C3

Figs 17 , 56A , 58

##### Types.

***Holotype***: ♀ (IZCAS), Thailand, Krabi Province, Muang District, Ban Klom Nong Thale Subdistrict, 8°8.1550'N, 98°48.4300'E, elevation ca 89 m, 26.X.2014, H. Zhao, Y. Li, Z. Chen leg.

##### Etymology.

The species name is a noun in apposition derived from the Chinese pinyin “dúmùzhōu” (canoe) and refers to the structure of the spermathecae which resembles a canoe.

##### Diagnosis.

**Figure 17.**
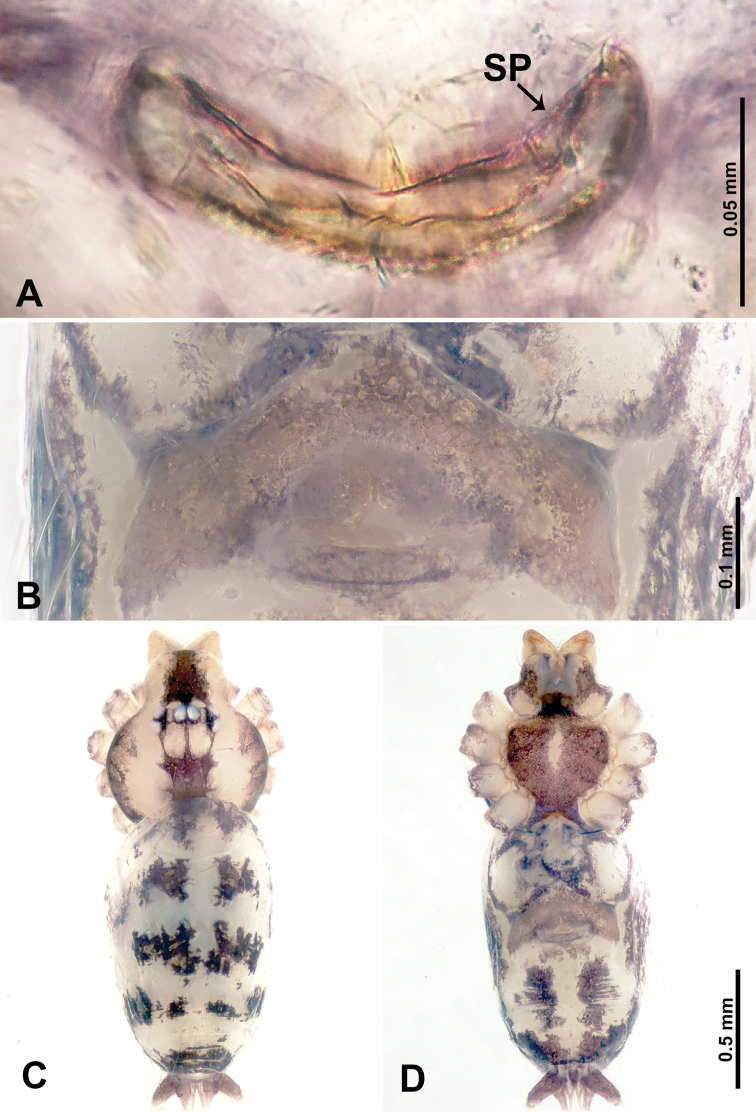
*Leclercera dumuzhou* sp. nov., female paratype. **A** Endogyne, dorsal view **B** female epigastric area, ventral view **C** female habitus, dorsal view **D** female habitus, ventral view. Abbreviation: SP = spermatheca.

Female of *L. dumuzhou* sp. nov. resembles the female of *L. banensis* sp. nov. but can be differentiated by the antero-ventral dark brown band surrounding the external genitalia which does not extend across the entire width of the abdomen (Fig. 17B) (vs. the dark brown transverse band fully covering the entire width of the abdomen (Fig. 15B)), spermathecae slightly curved anteriorly with tip directed laterally (Fig. 17A) (vs. spermathecae flattened with tubular extensions laterally (Fig. 15A)).

##### Description.

**Female.** Total length 1.76; carapace 0.63 long, 0.70 wide; abdomen 1.13 long, 0.70 wide. Carapace round and brown, with three longitudinal dark brown bands, median band 3 times wider than lateral bands, anterior with trident of dark brown stripes (Fig. 17C). Chelicerae brown (Fig. 56A). Clypeus dark brown medially, light brown laterally. Endites dark brown, delimiting circular light brown spots basally. Labium dark brown. Sternum dark brown with median light brown strip. Abdomen elongated, dorsum with a few pairs of dark brown patches (Fig. 17C), antero-ventrally with a pair of dark brown circular lines laterally, external genitalia region dark brown with band, posterior with a pair of dark brown patches (Fig. 17D). Legs uniformly brown; measurements: I missing, II 5.73 (1.63, 0.20, 1.60, 1.60, 0.70), III 4.10 (1.20, 0.20, 1.00, 1.10, 0.60), IV 6.10 (1.72, 0.25, 1.75, 1.63, 0.75). Epigastric area (Fig. 17B): dark brown band delimiting a light brown triangle medially. Endogyne (Fig. 17A): spermathecae transverse, slightly curved upwards, with pointed tips.

**Male**. Unknown.

##### Distribution.

Known only from the type locality (Fig. 58).

#### Leclercera suwanensis sp. nov.

Taxon classificationAnimaliaAraneaePsilodercidae

301AE5D0-80B7-5BB6-97B9-7DDD1CA29500

http://zoobank.org/1A2BC8D6-607F-4F10-BF56-97D0746BC611

**Figure 18.**
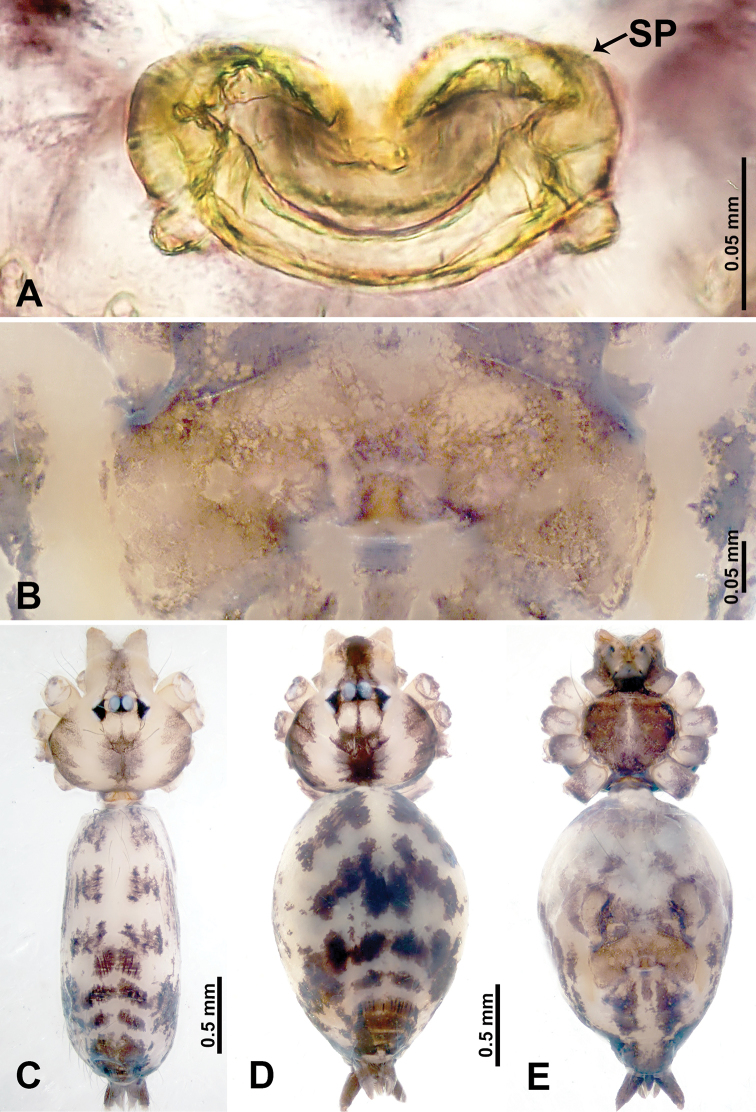
*Leclercera suwanensis* sp. nov. male holotype and female paratype. **A** Endogyne, dorsal view **B** female epigastric area, ventral view **C** male habitus, dorsal view **D** female habitus, dorsal view **E** female habitus, ventral view. Abbreviation: SP = spermatheca.

**Figure 19.**
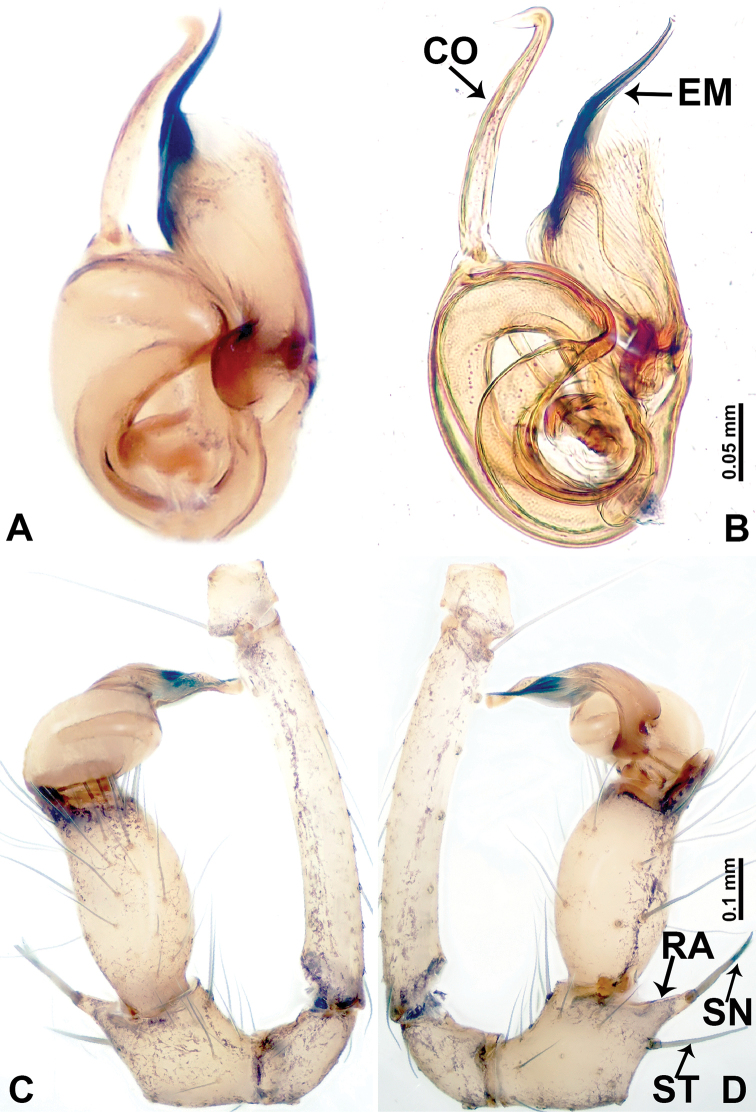
*Leclercera suwanensis* sp. nov. **A** Palp, ventral view **B** bulb, ventral view **C** palp, prolateral view **D** palp, retrolateral view. Abbreviations: CO = conductor, EM = embolus, RA = retrolateral apophysis, SN = spine, ST = strong seta.

Figs 18 , 19 , 55J , 58

##### Types.

***Holotype***: ♂ (IZCAS), Thailand, Phangnga Province, Takuathung District, Suwankuha Cave, 8°25.7695'N, 98°28.2693'E, elevation ca 19 m, 9.X.2015, Q. Zhao, G. Zhou and Z. Chen leg. ***Paratype***: 1♀ (IZCAS), same data as holotype.

##### Etymology.

The species name is an adjective referring to the type locality.

##### Diagnosis.

Diagnostic features of males and females are discussed in the diagnosis of *L. banensis* sp. nov.

##### Description.

**Male** (Holotype). Total length 2.45; carapace 0.70 long, 0.86 wide; abdomen 1.75 long, 0.78 wide. Carapace round and pale brown, with three longitudinal bands, median band 2 times wider than the lateral bands (Fig. 18C). Chelicerae pale brown (Fig. 55J). Clypeus slanting, dark brown medially, light brown laterally. Endites dark brown, light brown basally. Labium dark brown. Sternum dark brown, delimiting a light brown strip medially. Abdomen elongated, dorsum with pairs of dark brown spots, ventrum with pair of oval brown patches laterally followed by inverted ‘U’-shaped brown band, posterior with a pair of dark brown patches. Legs uniformly brown; measurements: I 11.17 (3.21, 0.31, 3.40, 3.25, 1.00), II 8.27 (2.40, 0.25, 2.50, 2.34, 0.78), III 5.72 (1.72, 0.31, 1.50, 1.56, 0.63), IV 8.41 (2.50, 0.31, 2.40, 2.40, 0.80). Palp (Fig. 19A–D): femur slender, five times longer than patella; patella not swollen; tibia half the length of femur, swollen and pentagonal in lateral view, with a retrolateral apophysis bearing a distal spine which is as long as the apophysis itself and a strong seta near the base of the retrolateral apophysis (Fig. 19D); cymbium half the width and length of the femur; bulb pale brown, bulging, ovoid, conductor and embolus separated, with conductor and embolus arising distally; conductor elongated and slightly hooked at the tip, as long as embolus; embolus elongated, basally swollen and progressively darkening and thinning to a pointed tip (Fig. 19B).

**Female** (Paratype). General features and coloration similar to those of male (Fig. 18D, E). Measurements: total length 1.70; carapace 0.50 long, 0.60 wide; abdomen 1.20 long, 1.00 wide. Leg measurements: I missing, II 6.65 (1.92, 0.20, 2.03, 1.72, 0.78), III 4.70 (1.40, 0.25, 1.25, 1.20, 0.60), IV 6.63 (2.00, 0.25, 1.88, 1.75, 0.75). Epigastric area (Fig. 18B): brown patch resembles an inverted ‘U’ shape. Endogyne (Fig. 18A): a pair of spermathecae curling towards each other, with pointed tips and rounded posterior extensions laterally.

##### Distribution.

Known only from the type locality (Fig. 58).

#### Leclercera maochong sp. nov.

Taxon classificationAnimaliaAraneaePsilodercidae

76935E75-DA5C-5E38-9319-B7C7B726606E

http://zoobank.org/90E05F42-497A-4109-9E10-1CB16F79F6E5

**Figure 20.**
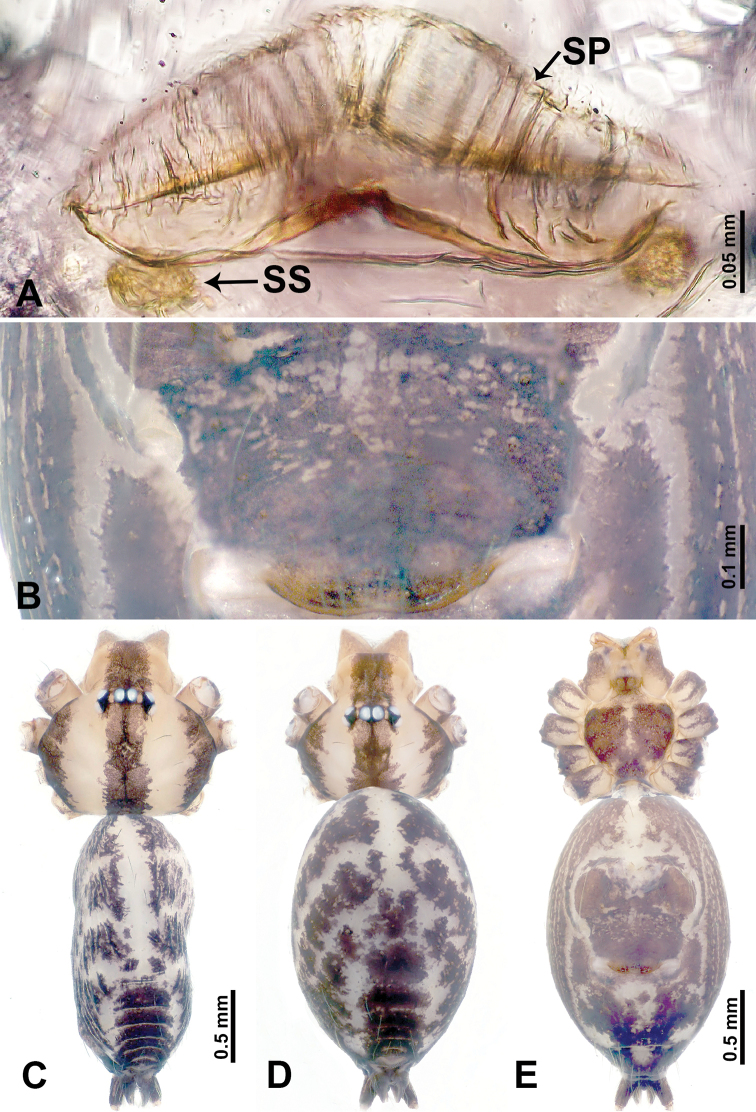
*Leclercera maochong* sp. nov., male holotype and female paratype. **A** Endogyne, dorsal view **B** female epigastric area, ventral view **C** male habitus, dorsal view **D** female habitus, dorsal view **E** female habitus, ventral view. Abbreviations: SP = spermatheca, SS = spherical structure.

**Figure 21.**
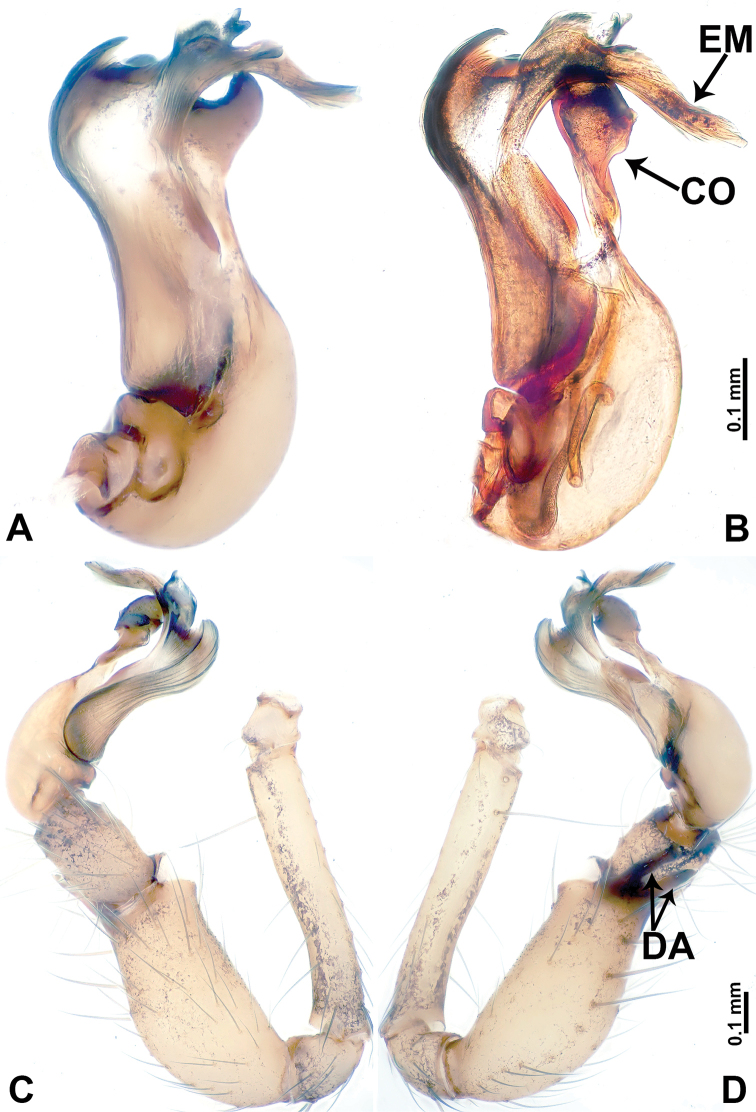
*Leclercera maochong* sp. nov. **A** Bulb, ventral view **B** bulb, ventral view **C** palp, prolateral view **D** palp, retrolateral view. Abbreviations: CO = conductor, DA = dorsal apophysis, EM = embolus.

Figs 20 , 21 , 55G , 58

##### Types.

***Holotype***: ♂ (IZCAS), China, Yunnan Province, Yuxi Town, Yuanjiang County, Yangchajiexiang Nature Reserves, Nanxi Region, 23°9.6320'N, 101°45.5640'E, elevation ca 2144 m, 4.VI.2015, Y. Li and Z. Chen leg. ***Paratype***: 1♀ (IZCAS), same data as holotype.

##### Etymology.

The species name is a noun in apposition derived from the Chinese pinyin “máochóng” (caterpillar) and refers to the structure of spermathecae which resemble a caterpillar in lateral view.

##### Diagnosis.

**Figure 22.**
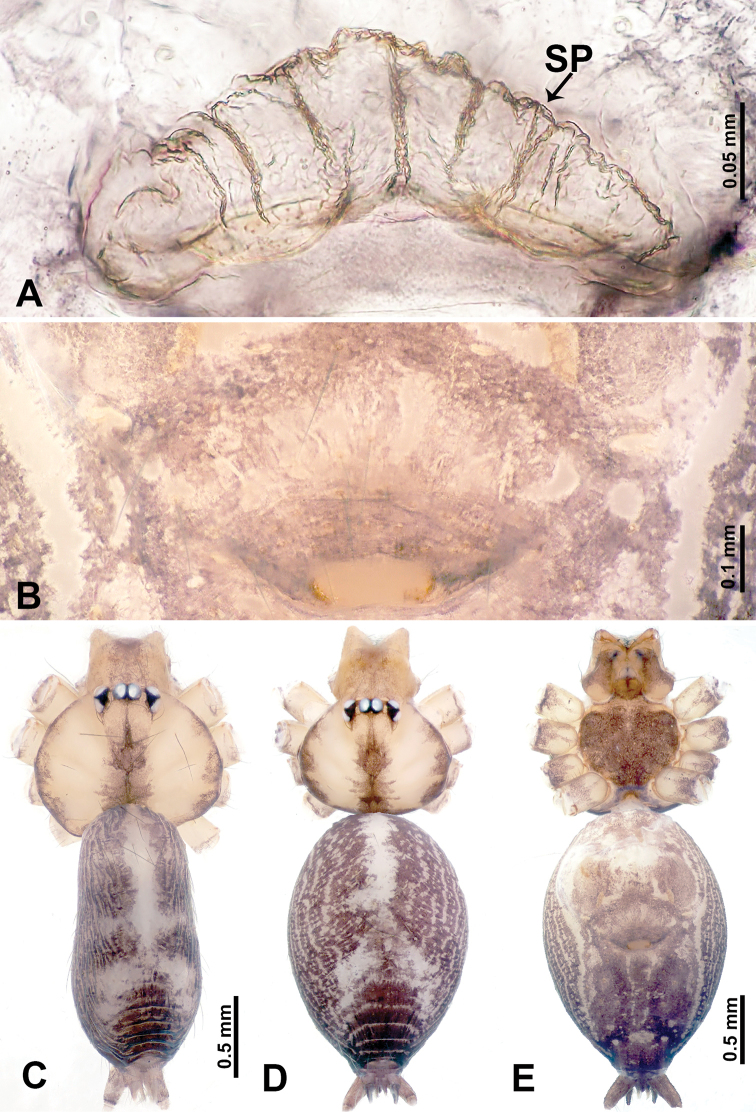
*Leclercera shanzi* sp. nov., male holotype and female paratype. **A** Endogyne, dorsal view **B** female epigastric area, ventral view **C** male habitus, dorsal view **D** female habitus, dorsal view **E** female habitus, ventral view. Abbreviation: SP = spermatheca.

**Figure 23.**
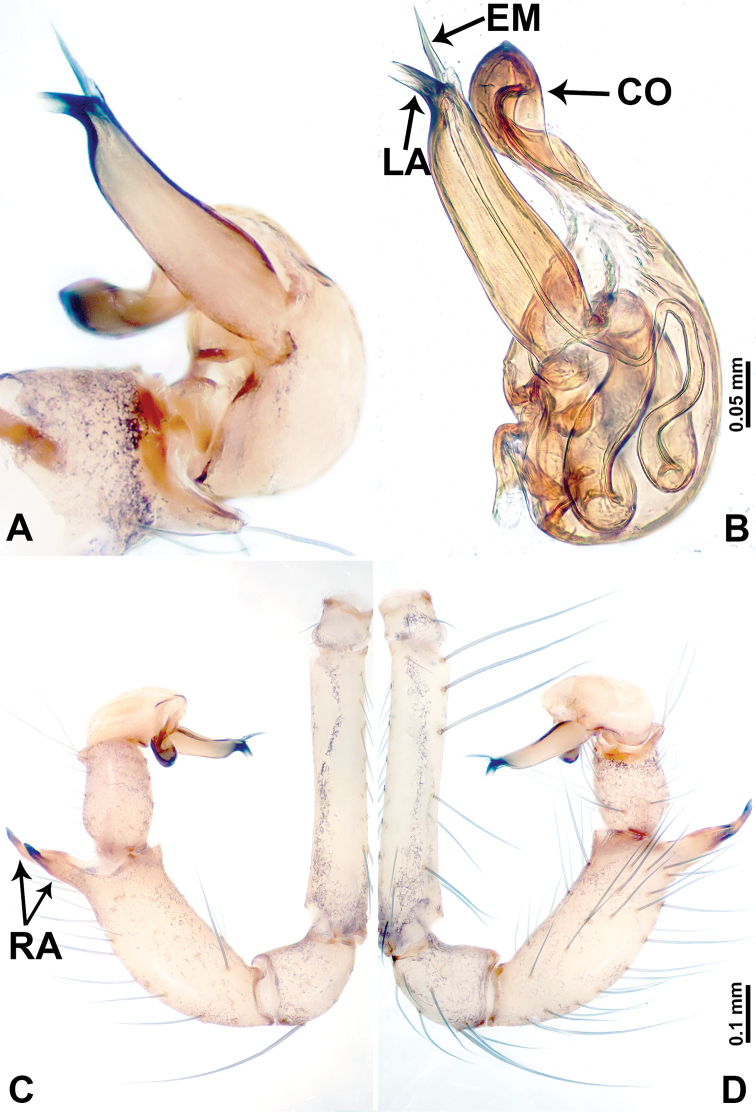
*Leclercera shanzi* sp. nov. **A** Palp, ventral view **B** bulb, ventral view **C** palp, prolateral view **D** palp, retrolateral view. Abbreviations: CO = conductor, EM = embolus, LA = laminar apophysis, RA = retrolateral apophyses.

Males of *L. maochong* sp. nov. resemble *L. shanzi* sp. nov. but can be distinguished by a pair of divided conductors (Fig. 21B) (i.e. – consisting of two components) (vs. a slightly twisted undivided conductor (Fig. 23B)), an absence of a laminar apophysis adjacent to embolus (vs. presence of a laminar apophysis adjacent to embolus), embolus two times longer than tegulum (vs. embolus of similar length to tegulum), presence of two dorsal apophyses anteriorly on tibia (Fig. 21D) (vs. the presence of two retrolateral tibial apophyses anteriorly (Fig. 23C)); females can be recognized by having more coils of the spermathecae and by having a pair of lateral, spherical structures connected via the duct system to the posterior ends of the spermathecae (Fig. 20A) (vs. an absence of posterior extensions of the spermathecae), external genitalia a dark purplish, rectangular patch (Fig. 20B) (vs. fan-shaped external genitalia (Fig. 22B)), dorsum with oblique lateral dark brown stripes (Fig. 20D) (vs. dark brown lateral bands with parallel lines on dorsum (Fig. 22D)).

##### Description.

**Male** (Holotype). Total length 2.58; carapace 0.88 long, 1.20 wide; abdomen 1.70 long, 0.80 wide. Carapace round and brown, with three dark brown longitudinal bands, median band two times wider than lateral band (Fig. 20C). Chelicerae brown (Fig. 55G). Clypeus with dark brown band medially. Endites dark brown, light brown basally. Labium dark brown. Sternum dark brown with vertically thin median light brown band. Abdomen elongated, dorsum with pairs of dark brown spots, posterior with dark brown horizontal stripes, antero-ventrally with a pair of ovoid brown patches followed by rectangular dark brown patch, posterior with complicated dark brown pattern. Legs uniformly brown; measurements: I–IV missing. Palp (Fig. 21A–D): femur slender, four times longer than patella; patella not swollen; tibia swollen, 1.5 times shorter and 3 times wider than femur, with two dorsal apophyses anteriorly (apophyses almost as long as cymbium); cymbium three times shorter than femur, dark brown distally; bulb brown, pyriform, with conductor and embolus arising distally; conductor comprises two components, basally and distally merged with embolus; embolus elongated and sheet-like, slightly twisted, two times longer than tegulum (Fig. 21B).

**Female** (Paratype). General features and coloration similar to those of male (Fig. 20D, E). Measurements: total length 2.66; carapace 0.78 long, 0.94 wide; abdomen 1.88 long, 1.25 wide. Leg measurements: I 8.84 (2.40, 0.31, 2.88, 2.00, 1.25), II 7.06 (2.00, 0.31, 2.03, 1.72, 1.00), III missing, IV 8.17 (2.40, 0.31, 2.34, 2.03, 1.09). Epigastric area (Fig. 20B): rectangular dark brown patch anteriorly, followed by brown crescent-shaped slit posteriorly. Endogyne (Fig. 20A): spermathecae resembles a crawling caterpillar in lateral view, with pointed ends, connected posteriorly to a pair of lateral spherical bodies by a duct system double-looped like a shallow “W”.

##### Distribution.

Known only from the type locality (Fig. 58).

#### Leclercera shanzi sp. nov.

Taxon classificationAnimaliaAraneaePsilodercidae

813AD6F3-B06D-57E7-B1E1-94A0D9CB2D8C

http://zoobank.org/DFBA723A-2878-4E9E-A4A7-2AC218788D09

Figs 22 , 23 , 55H , 58

##### Types.

***Holotype***: ♂ (IZCAS), China, Yunnan Province, Wenshan State, Pingbian County, outside of Dawei Mountain Provincial Nature Reserves, 22°54.6450'N, 103°41.7810'E, elevation ca 2070 m, 21.V.2015, Y. Li and Z. Chen leg. ***Paratype***: 1♀ (IZCAS), same data as holotype.

##### Etymology.

The species name is a noun in apposition derived from the Chinese pinyin “shànzĭ” (fan) and refers to the resemblance of the external genitalia to a hand fan.

##### Diagnosis.

Diagnostic features of males and females are discussed in the diagnosis of *L. maochong* sp. nov.

##### Description.

**Male** (Holotype). Total length 2.63; carapace 1.00 long, 1.13 wide; abdomen 1.63 long, 0.80 wide. Carapace round and brown, with three dark brown longitudinal bands, median band five times wider than lateral band (Fig. 22C). Chelicerae brown (Fig. 55H). Clypeus with dark brown band medially. Endites dark brown delimiting light brown circular area basally. Labium dark brown. Sternum dark brown. Abdomen elongated, dorsum medially with dark brown lateral patches with parallel lines delimiting an inverted “Y”-shape (Fig. 22C), antero-ventrally with a pair of dark brown kidney-shaped lateral spots, followed by a fan-shaped dark brown region, with an indistinct dark brown pattern posteriorly. Legs uniformly brown; measurements: I 14.78 (4.25, 0.40, 4.50, 4.00. 1.63), II 13.17 (3.85, 0.40, 4.17, 3.25, 1.50), III 8.94 (2.75, 0.31, 2.60, 2.03, 1.25), IV missing. Palp (Fig. 23A–D): femur slender, three times longer than patella; patella not swollen; tibia swollen, 1.5 times shorter and wider than femur, with two retrolateral apophyses anteriorly, apophyses darken distally (Fig. 23C); cymbium three times shorter than femur; bulb brown, pyriform, with conductor, embolus, and laminar apophysis arising distally; conductor twisted distally, with blunt tip, slightly shorter than, and basally merged with, embolus; embolus stalk almost as long as tegulum, embolus finely pointed; laminar apophysis black, adjacent to embolus, basally merged with and slightly wider than embolus (Fig. 23B).

**Female** (Paratype). General features and coloration similar to those of male (Fig. 22D, E). Measurements: total length 2.60; carapace 0.80 long, 1.00 wide; abdomen 1.80 long, 1.25 wide. Leg measurements: I 13.15 (3.50, 0.40, 4.00, 3.50, 1.75), II 11.06 (3.21, 0.40, 3.25, 2.80, 1.40), III 7.85 (2.34, 0.31, 2.20, 1.80, 1.20), IV 10.91 (3.20, 0.31, 3.20, 2.80, 1.40). Epigastric area (Fig. 22B): purplish and brownish pattern resembling the shape of a hand fan. Endogyne (Fig. 22A): pair of spermathecae resembling an isosceles triangle but without flattened base, base convex, both ends rounded.

##### Distribution.

Known only from the type locality (Fig. 58).

#### Leclercera duandai sp. nov.

Taxon classificationAnimaliaAraneaePsilodercidae

32A1D380-8B18-5006-BC28-CD704A598D49

http://zoobank.org/0106710C-BC7E-4174-8DBB-ADE9FF54715B

Figs 24 , 55K , 58

##### Types.

***Holotype***: ♀ (IZCAS), China, Tibet Autonomous Region, Nyingchi, Medog County, Beibung Village, around Jiagagou Bridge, 29°15.0670'N, 95°11.7170'E, elevation ca 805 m, 18.VI.2016, J. Wu leg.

##### Etymology.

**Figure 24.**
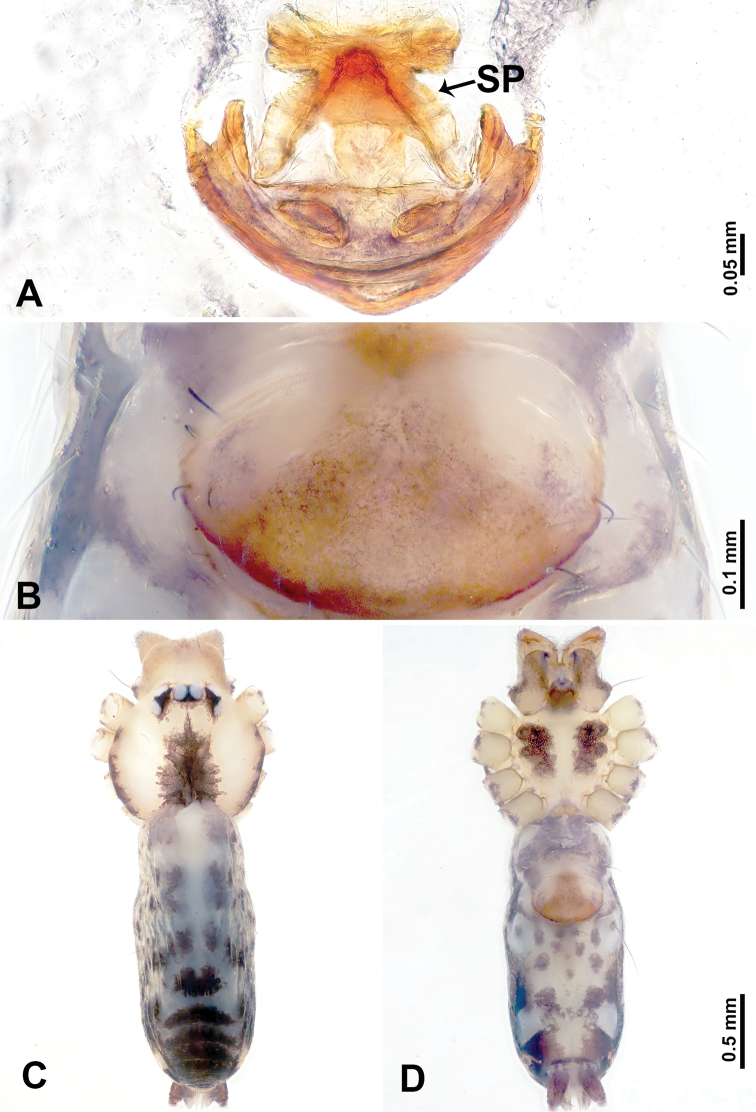
*Leclercera duandai* sp. nov., female paratype. **A** Endogyne, dorsal view **B** female epigastric area, ventral view **C** female habitus, dorsal view **D** female habitus, ventral view. Abbreviation: SP = spermatheca.

The species name is a noun in apposition derived from the Chinese pinyin “duàndài” (ribbon) and refers to the structure of the spermathecae resembling a ribbon knot (Fig. 24A).

##### Diagnosis.

Females of *L. duandai* sp. nov. can be distinguished from other congeners by the unique orange coloration of the external genitalia and the spermathecae (Fig. 24A, B) which resemble a pair of orange-coloured ribbon knots (vs. absence of orange coloration on the external genitalia or spermathecae in congeners).

##### Description.

**Female.** Total length 2.88; carapace 0.96 long, 1.00 wide; abdomen 1.92 long, 0.80 wide. Carapace round and brown, with three longitudinal dark brown bands, median band 8 times wider than the lateral bands (Fig. 24C). Chelicerae brown (Fig. 55K). Clypeus light brown. Endites light brown, dark brown marginally. Labium dark brown. Sternum light brown, with longitudinal dark brown spots laterally. Abdomen elongated, dorsum with dark brown spots, with posterior dark brown stripes medially (Fig. 24C), antero-ventrally with ovoid, orangish external genitalia, with scattered dark brown spots posteriorly (Fig. 24D). Leg measurements: I–IV missing. Epigastric area (Fig. 24B): an elliptical, orangish patch. Endogyne (Fig. 24A): a pair of spermathecae resembling a ribbon knot, posterior receptacles with a pair of ovoid bodies with two-branched, upturned ends.

**Male**. Unknown.

##### Distribution.

Known only from the type locality (Fig. 58).

#### Leclercera hponensis sp. nov.

Taxon classificationAnimaliaAraneaePsilodercidae

D28E4D44-57E1-5694-9D48-05D7490BEAE0

http://zoobank.org/8B33B61D-7B2C-4632-A1D8-9A6F335B04F3

**Figure 25.**
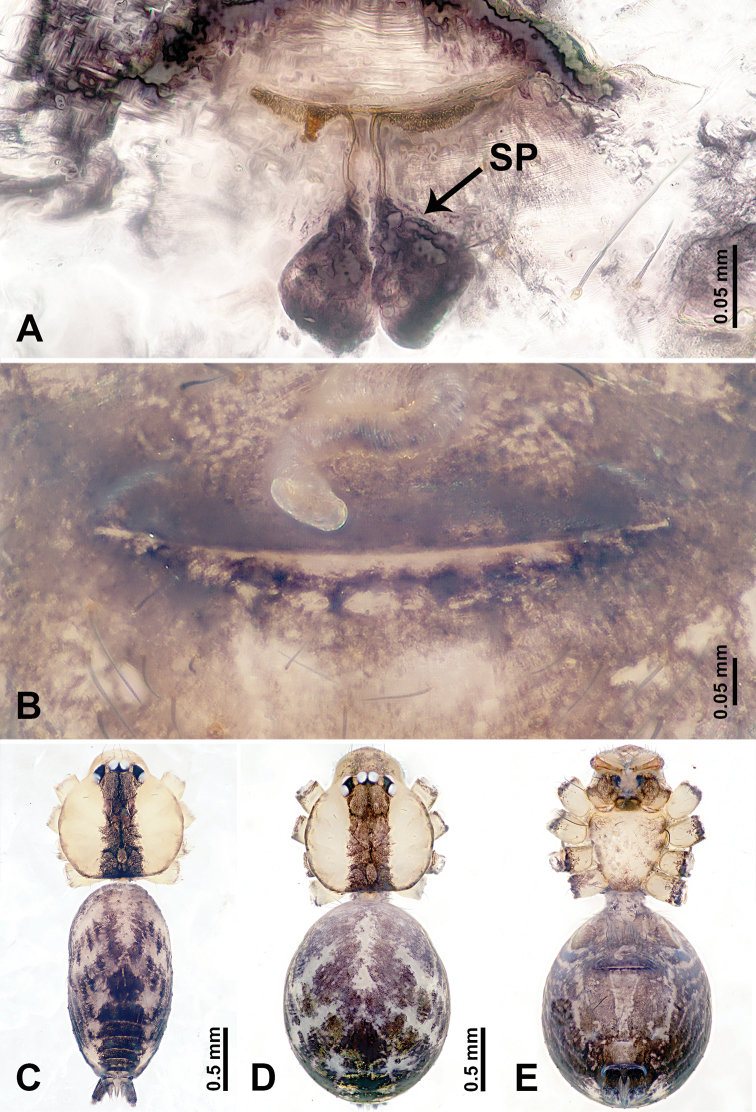
*Leclercera hponensis* sp. nov., male holotype and female paratype. **A** Endogyne, dorsal view **B** female epigastric area, ventral view **C** male habitus, dorsal view **D** female habitus, dorsal view **E** female habitus, ventral view. Abbreviation: SP = spermatheca.

**Figure 26.**
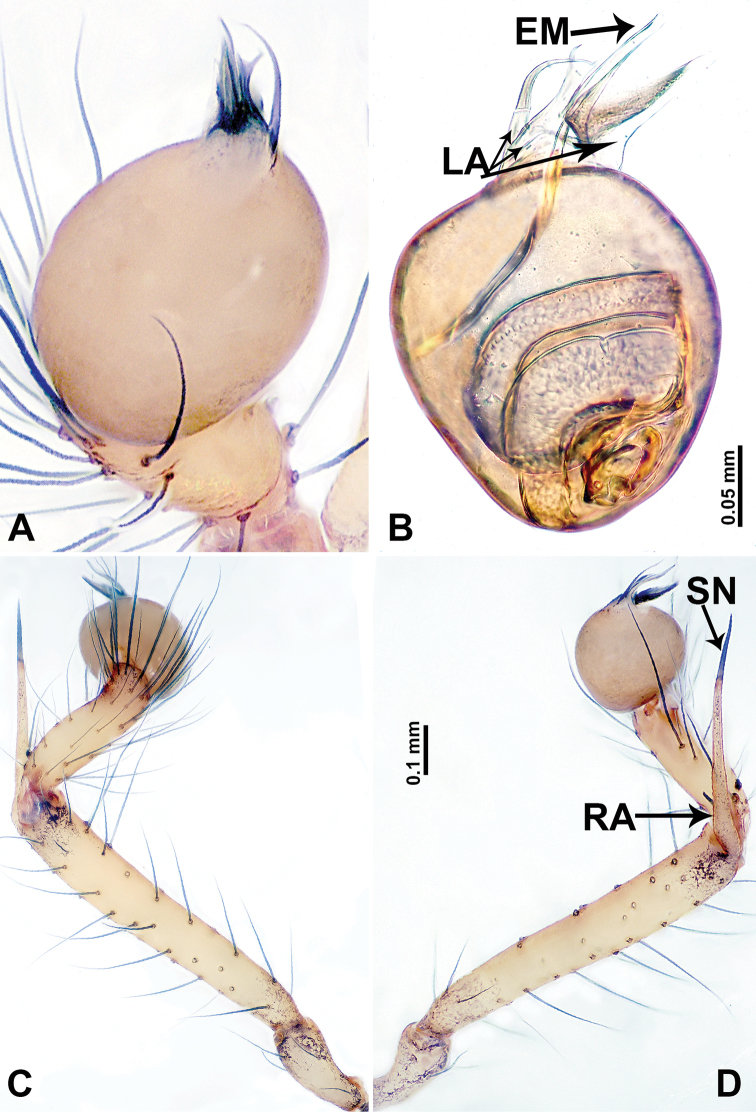
*Leclercera hponensis* sp. nov. **A** Palp, ventral view **B** bulb, ventral view **C** palp, prolateral view **D** palp, retrolateral view. Abbreviations: EM = embolus, LA = laminar apophysis, RA = retrolateral apophysis, SN = spine.

Figs 25 , 26 , 56F , 58

##### Types.

***Holotype***: ♂ (IZCAS), Myanmar, Kachin State, Putao Town, Hponkanrazi Wildlife Sanctuary Roadside between Camp 2 to Camp 3, 27°37.1500'N, 96°58.9170'E, elevation ca 2806 m, 16.XII.2016, J. Wu leg. ***Paratype***: 1♀ (IZCAS), same data as holotype.

##### Etymology.

The species name is an adjective referring to the type locality.

##### Diagnosis.

Males of *L. hponensis* sp. nov. can be distinguished from congeners by the presence of a three-branched, laminar apophysis on the distal end of the bulb (Fig. 26B) (vs. the absence of a three-branched laminar apophysis in congeners), a spheroid bulb (vs. the bulb of congeners have other shapes), tibia with a retrolateral apophysis bearing a spine that is three times shorter than the apophysis, the entire apophysis, including the spine, is two times longer than the tegulum (Fig. 26D) (vs. the absence of such a combination of a tibia apophysis and spine in congeners); the female can be differentiated from congeners by the pair of stalked spermathecae with a triangular distal part (Fig. 25A).

##### Description.

**Male** (Holotype). Total length 2.30; carapace 0.80 long, 0.88 wide; abdomen 1.50 long, 0.75 wide. Carapace round and brown, with dark brown longitudinal median band (Fig. 25C). Chelicerae brown (Fig. 56F). Clypeus light brown. Endites dark brown, light brown basally. Labium dark brown. Sternum light brown. Abdomen elongated, antero-dorsally with dark brown spots, posterior with dark brown stripes medially, antero-ventrally with dark brown elliptical patch, posterior with indistinct dark brown pattern. Legs uniformly brown; measurements: I 8.97 (2.53, 0.31, 2.81, 2.19, 1.13), II 7.10 (1.94, 0.31, 2.19, 1.72, 0.94), III 5.25 (1.44, 0.31, 1.47, 1.25, 0.78), IV 7.97 (2.06, 0.31, 2.13, 2.38, 1.09). Palp (Fig. 26A–D): femur slender, four times longer than patella; patella not swollen; tibia 1.5 times shorter than femur, with retrolateral apophysis anteriorly bearing a spine, two times longer than tegulum, spine three times shorter than apophysis (Fig. 26D); cymbium three times shorter than femur; bulb brown, spheroid, with embolus and laminar apophysis arising distally; three-branched, laminar apophyses almost equal in length but shorter than embolus; embolus laminar, longer than all other laminar apophyses of its own (Fig. 26B).

**Female** (Paratype). General features and coloration similar to those of male (Fig. 25D, E). Measurements: total length 2.40; carapace 0.84 long, 0.94 wide; abdomen 1.56 long, 1.19 wide. Leg measurements: I 7.75 (2.06, 0.31, 2.44, 1.88, 1.06), II 6.47 (1.72, 0.31, 1.94, 1.56, 0.94), III 4.53 (1.25, 0.31, 1.13, 1.09, 0.75), IV 6.44 (1.72, 0.31, 1.88, 1.56, 0.97). Epigastric area (Fig. 25B): dark brown patch delimiting a horizontal light brown slit. Endogyne (Fig. 25A): a pair of stalked spermathecae with anterior stalks and posteriorly a triangular distal part.

##### Distribution.

Known only from the type locality (Fig. 58).

#### Leclercera lizi sp. nov.

Taxon classificationAnimaliaAraneaePsilodercidae

CD38BD03-0041-5CC9-AA82-E39BBFACC761

http://zoobank.org/5CA634EB-DCB3-4CEA-803A-167BFBBFFC20

**Figure 27.**
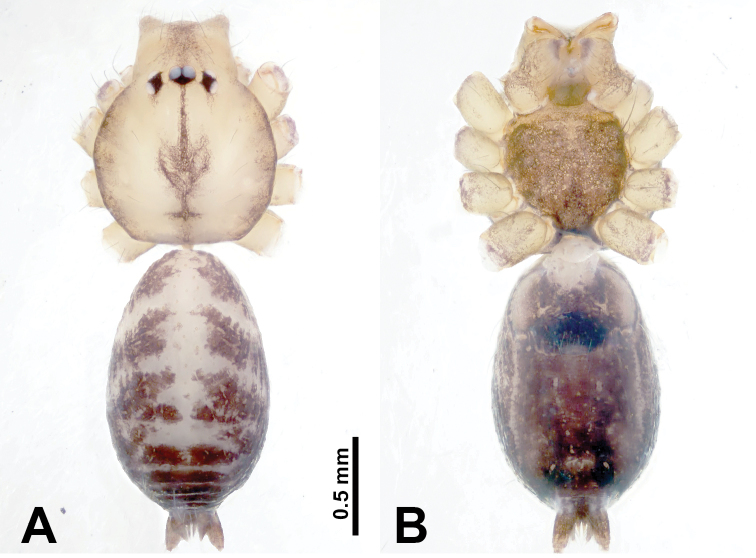
*Leclercera lizi* sp. nov., male holotype. **A** Habitus, dorsal view **B** habitus ventral view.

**Figure 28.**
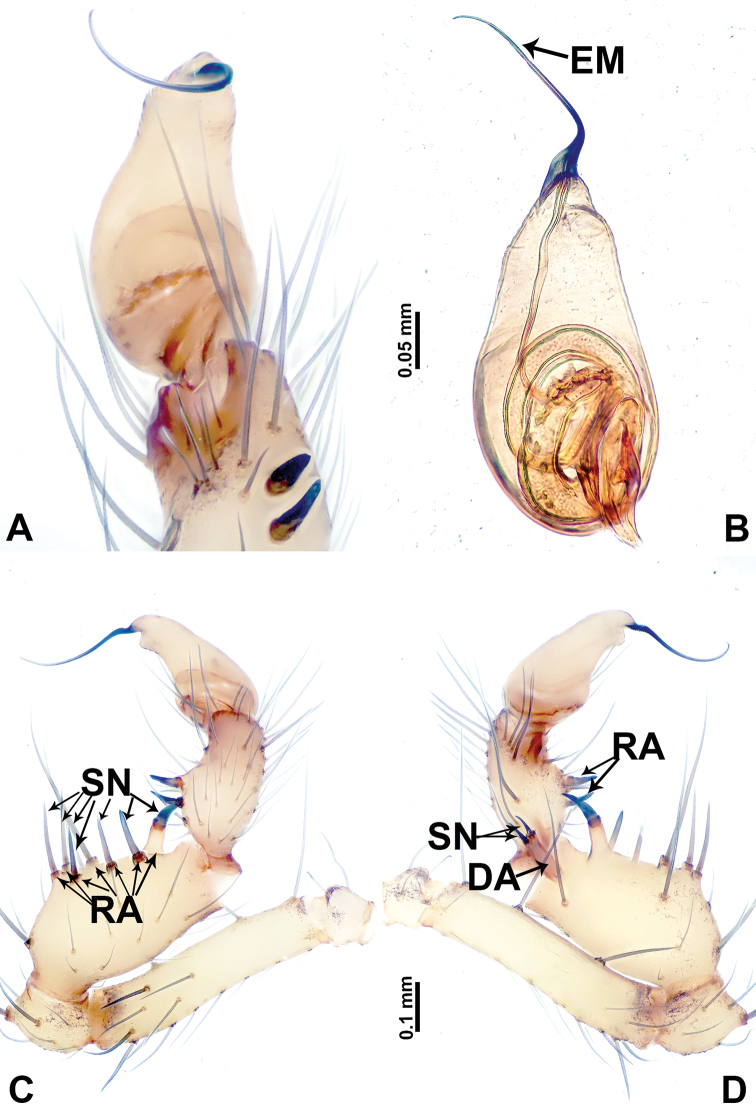
*Leclercera lizi* sp. nov. **A** Palp, ventral view **B** bulb, ventral view **C** palp, prolateral view **D** palp, retrolateral view. Abbreviations: EM = embolus, DA = dorsal apophysis, RA = retrolateral apophyses, SN = spines.

Figs 27 , 28 , 56G , 58

##### Types.

***Holotype***: ♂ (IZCAS), China, Tibet Autonomous Region, Xigaze, Dinggye County, Changga Village, 27°51.6290'N, 87°25.4802'E, elevation ca 2239 m, 7.VIII.2017, X. Zhang, Z. Bai leg.

##### Etymology.

The species name is a noun in apposition derived from the Chinese pinyin “lízĭ” (pear) and refers to the structure of the bulb resembling a pear (Fig. 28B).

##### Diagnosis.

Males of *L. lizi* sp. nov. can be distinguished from congeners by the abundance of apophyses and spines on the palp (Fig. 28C, D): cymbium with two retrolateral apophyses posteriorly, seven retrolateral apophyses on the swollen tibia with an anterior dorsal apophysis bearing two spines; embolus almost as long as the tegulum (Fig. 28B) (vs. the absence of congeners with such a profusion of apophyses and spines).

##### Description.

**Male** (Holotype). Total length 2.13; carapace 0.88 long, 0.90 wide; abdomen 1.25 long, 0.90 wide. Carapace round and brown, with three dark brown longitudinal bands, median band three times wider than lateral bands (Fig. 27A). Chelicerae brown (Fig. 56G). Clypeus light brown, with a trace of dark brown medially. Endites dark brown, light brown basally. Labium and sternum dark brown. Abdomen elongated, antero-dorsally with three pairs of dark brown spots laterally, posterior with dark brown stripes medially, antero-ventrally with black, elliptical patch delimiting kidney-shaped, light brown patch laterally, posterior with indistinct dark brown pattern. Legs uniformly brown; measurements: I–II missing, III 4.19 (1.25, 0.31, 0.94, 0.94, 0.75), IV 6.00 (1.60, 0.40, 1.60, 1.40, 1.00). Palp (Fig. 28A–D): femur slender, three times longer than patella; patella not swollen; tibia swollen, 1.2 times shorter and twice wider than femur, with seven anterior retrolateral apophyses bearing spines, anterior-most with longest apophysis and widest spine, antero-dorsally with dark brown apophysis bearing two spines resembling a fork; cymbium dark brown anteriorly, with two retrolateral apophyses posteriorly; bulb brown, pyriform with embolus arising distally, embolus thin and black, rather spiralled, almost equal in length to tegulum (Fig. 28B).

**Female**. Unknown.

##### Distribution.

Known only from the type locality (Fig. 58).

#### Leclercera xiaodai sp. nov.

Taxon classificationAnimaliaAraneaePsilodercidae

8E243F0B-6D0B-5969-AC61-E343570A6BC1

http://zoobank.org/C8700959-8BAE-49AD-9FA0-3300650DB5FC

Figs 29 , 55A , 58

##### Types.

***Holotype***: ♀ (IZCAS), China, Tibet Autonomous Region, Nyingchi, Bomê County, around Zhamo Town, 29°50.8590'N, 95°45.8610'E, elevation ca 2800 m, 17.VII.2013, Y. Lin leg.

##### Etymology.

**Figure 29.**
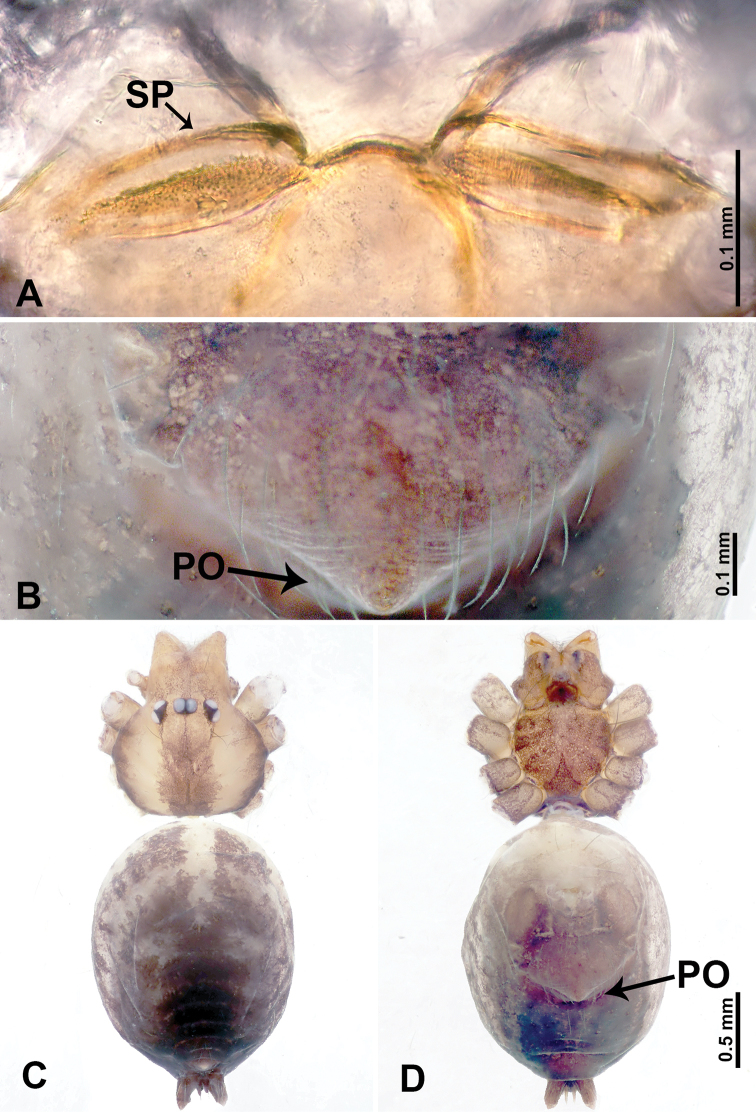
*Leclercera xiaodai* sp. nov., female paratype. **A** Endogyne, dorsal view **B** female epigastric area, ventral view **C** female habitus, dorsal view **D** female habitus, ventral view. Abbreviations: PO = pouch, SP = spermatheca.

The species name is a noun in apposition derived from the Chinese pinyin “xiǎodài” (small pouch) and refers to the distinct, pouch-like structure of the external genitalia (Fig. 29B).

##### Diagnosis.

Females of *L. duandai* sp. nov. can be distinguished from other congeners by the unique, pouch-like external genitalia (Fig. 29B) and a pair of fusiform spermathecae with two pairs of apophyses extending both anteriorly and posteriorly (Fig. 29A) (vs. an absence of pouch-like external genitalia in congeners).

##### Description.

**Female.** Total length 2.44; carapace 0.88 long, 0.90 wide; abdomen 1.56 long, 1.25 wide. Carapace round and brown, with three longitudinal dark brown bands, median band three times wider than lateral bands (Fig. 29C). Chelicerae brown (Fig. 55A). Clypeus light brown. Endites, labium, and sternum dark brown. Abdomen elongated, dorsum with indistinct dark brown spots (Fig. 29C), antero-ventrally with a pair of rounded dark brown patches laterally, followed by cone-shaped external genitalia resembling a small pouch, posterior dark brown (Fig. 29D). Legs uniformly brown; measurements: I 7.09 (2.03, 0.31, 2.19, 1.56, 1.00), II missing, III 4.53 (1.25, 0.31, 1.25, 1.09, 0.63), IV 6.64 (1.80, 0.31, 2.03, 1.56, 0.94). Epigastric area (Fig. 29B): inverted triangular pouch. Endogyne (Fig. 29A): pair of connected, fusiform spermathecae with anterior and posterior extensions.

**Male**. Unknown.

##### Distribution.

Known only from the type locality (Fig. 58).

#### Leclercera yanjing sp. nov.

Taxon classificationAnimaliaAraneaePsilodercidae

E1ED95BA-B7C7-5539-A24F-4EB5070DD500

http://zoobank.org/A4C77193-1588-4F4C-9646-3A82B6F532BC

Figs 30 , 55L , 58

##### Types.

***Holotype***: ♀ (IZCAS), China, Tibet Autonomous Region, Shannan, Cona County, Lemenba Ethnic Village, along the road between Lewang Bridge to Zhisimuzha scenic area, 27°49.5710'N, 91°43.7560'E, elevation ca 2793 m, 1.VI.2016, J. Wu leg.

##### Etymology.

**Figure 30.**
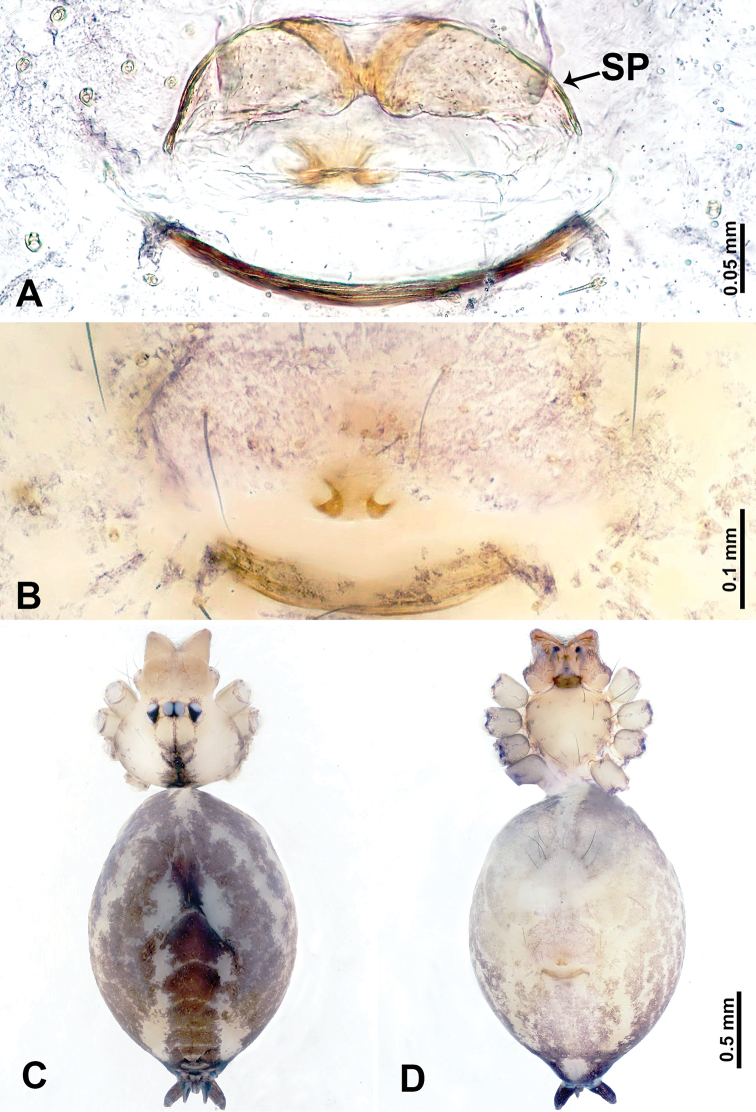
*Leclercera yanjing* sp. nov., female paratype. **A** Endogyne, dorsal view **B** female epigastric area, ventral view **C** female habitus, dorsal view **D** female habitus, ventral view. Abbreviation: SP = spermatheca.

The species name is a noun in apposition derived from the Chinese pinyin “yǎnjìng” (spectacles) and refers to the structure of the spermathecae resembling a pair of spectacles (Fig. 30A).

##### Diagnosis.

Females of *L. yanjing* sp. nov. can be distinguished from other congeners by a unique curved ‘x’ spot on the external genitalia (Fig. 30B) and the spermathecae which resemble a pair of aviator glasses (Fig. 30A) (vs. absence of such characteristics in congeners).

##### Description.

**Female.** Total length 2.88; carapace 0.88 long, 0.88 wide; abdomen 2.00 long, 1.50 wide. Carapace round and brown, with dark brown band medially (Fig. 30C). Chelicerae brown (Fig. 55L). Clypeus light brown. Endites dark brown, light brown basally. Labium dark brown. Sternum light brown. Abdomen elongated, dorsum with dark brown band laterally, medially with dark brown stripes (Fig. 30C), antero-ventrally pale brown with a distinct curved ‘x’ spot on external genitalia, with indistinct brown spots posteriorly (Fig. 30D). Legs uniformly brown; measurements: I 7.34 (2.03, 0.31, 2.19, 1.72, 1.09), II 6.57 (1.88, 0.31, 1.88, 1.56, 0.94), III 4.98 (1.41, 0.31, 1.28, 1.20, 0.78), IV 6.91 (1.88, 0.31, 2.00, 1.72, 1.00). Epigastric area (Fig. 30B): ovoid pinkish patch followed by a curvy ‘x’ mark and a brown slit posteriorly. Endogyne (Fig. 30A): a pair of connected, cuneate spermathecae resembling a pair of aviator glasses, with a curved ‘x’ spot posteriorly.

**Male**. Unknown.

##### Distribution.

Known only from the type locality (Fig. 58).

#### Leclercera ekteenensis sp. nov.

Taxon classificationAnimaliaAraneaePsilodercidae

E86053A2-BAC9-594A-9F98-1FFA75815B63

http://zoobank.org/8091EA7F-694F-489F-9384-633264AC554B

**Figure 31.**
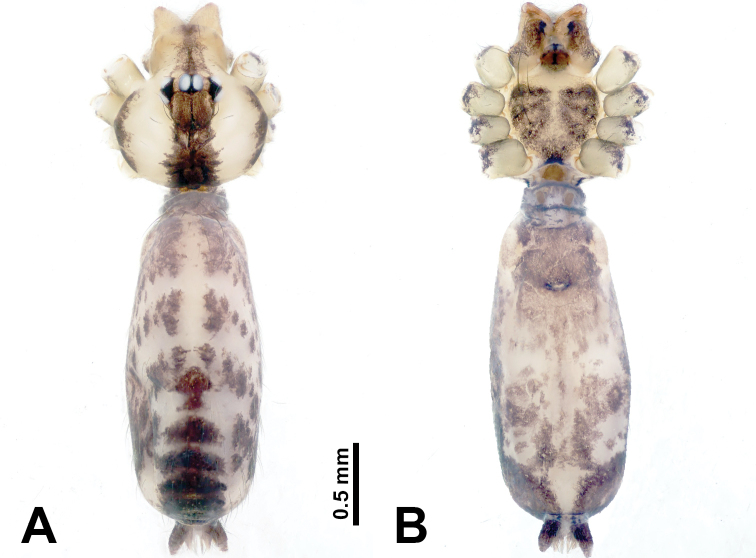
*Leclercera ekteenensis* sp. nov., male holotype. **A** Habitus, dorsal view **B** habitus, ventral view.

**Figure 32.**
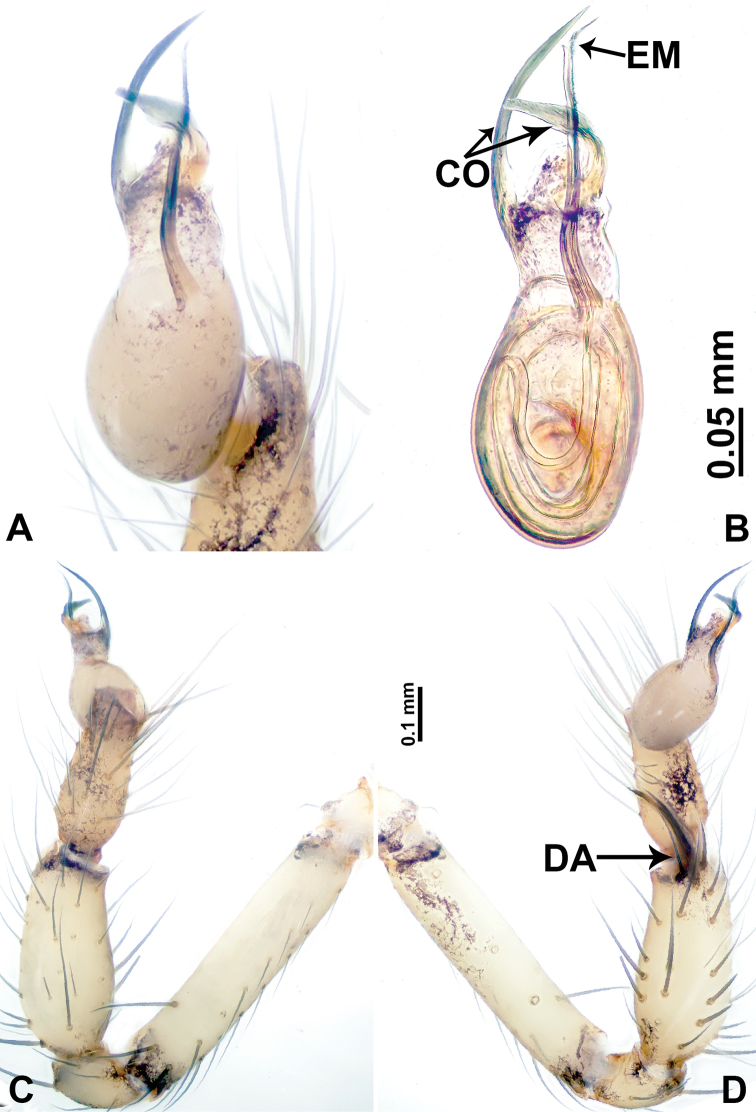
*Leclercera ekteenensis* sp. nov. **A** Palp, ventral view **B** bulb, ventral view **C** palp, prolateral view **D** palp, retrolateral view. Abbreviations: CO = conductor, DA = dorsal apophysis, EM = embolus.

Figs 31 , 32 , 56B , 58

##### Types.

***Holotype***: ♂ (IZCAS), Nepal, Mechi District, Ekteen Village, 27°13.1333'N, 87°50.7833'E, elevation ca 2088 m, 27.XI.2016, Q. Zhao leg.

##### Etymology.

The species name is an adjective referring to the type locality.

##### Diagnosis.

Males of *L. ekteenensis* sp. nov. can be distinguished from congeners by the presence of a conductor with two distinct branches (Fig. 32B) (vs. the absence of a two-branched conductor in congeners), the embolus arising from medial tegulum (vs. embolus arising distally in congeners), the presence of a distinct darkened apophysis anteriorly on the tibia (Fig. 32D) (vs. the absence of a darkened tibial apophysis in congeners).

##### Description.

**Male** (Holotype). Total length 3.09; carapace 0.90 long, 1.00 wide; abdomen 2.19 long, 0.94 wide. Carapace round and brown, with three dark brown longitudinal bands, median band five times wider than the lateral bands (Fig. 31A). Chelicerae brown (Fig. 56B). Clypeus light brown, with dark brown band medially. Endites dark brown, light brown basally. Labium dark brown. Sternum with dark brown stripes laterally, delimiting light brown anterior and median region. Abdomen elongated, antero-dorsally with three pairs of dark brown spots laterally, light brown medially, posterior with dark brown stripes medially, antero-ventrally with dark brown elliptical patch, posterior with indistinct dark and light brown spots, posterior edge with a pair of dark brown fusiform patches laterally. Legs uniformly brown; measurements: I 12.76 (3.40, 0.31, 3.60, 3.53, 1.92), II 8.71 (2.40, 0.31, 2.40, 2.40, 1.20), III 6.39 (1.88, 0.25, 1.75, 1.63, 0.88), IV 9.82 (2.80, 0.31, 2.80, 2.66, 1.25). Palp (Fig. 32A–D): femur slender, four times longer than patella; patella not swollen; tibia swollen, 1.5 times shorter and wider than femur, antero-dorsally with a black apophysis, almost as long as tegulum; cymbium with dark brown spots, two times shorter than femur; bulb light brown, pyriform, with conductor arising distally, embolus arising medially; conductor dark and thin with two branches, one shorter than the other, short branch bends towards long branch; embolus arising from median tegulum, attached to short branch of conductor, almost as long as long branch of the conductor (Fig. 32B).

**Female**. Unknown.

##### Distribution.

Known only from the type locality (Fig. 58).

#### Leclercera zhamensis sp. nov.

Taxon classificationAnimaliaAraneaePsilodercidae

C41CBD3D-4B53-55EA-9B8C-4C7F0576E221

http://zoobank.org/9B9A0613-F125-4FD6-870D-A84219EA8955

**Figure 33.**
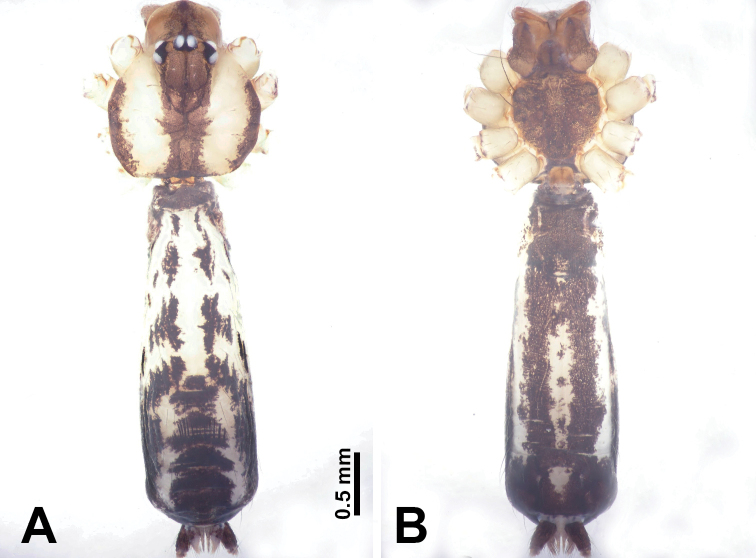
*Leclercera zhamensis* sp. nov., male holotype. **A** Habitus, dorsal view **B** habitus ventral view.

**Figure 34.**
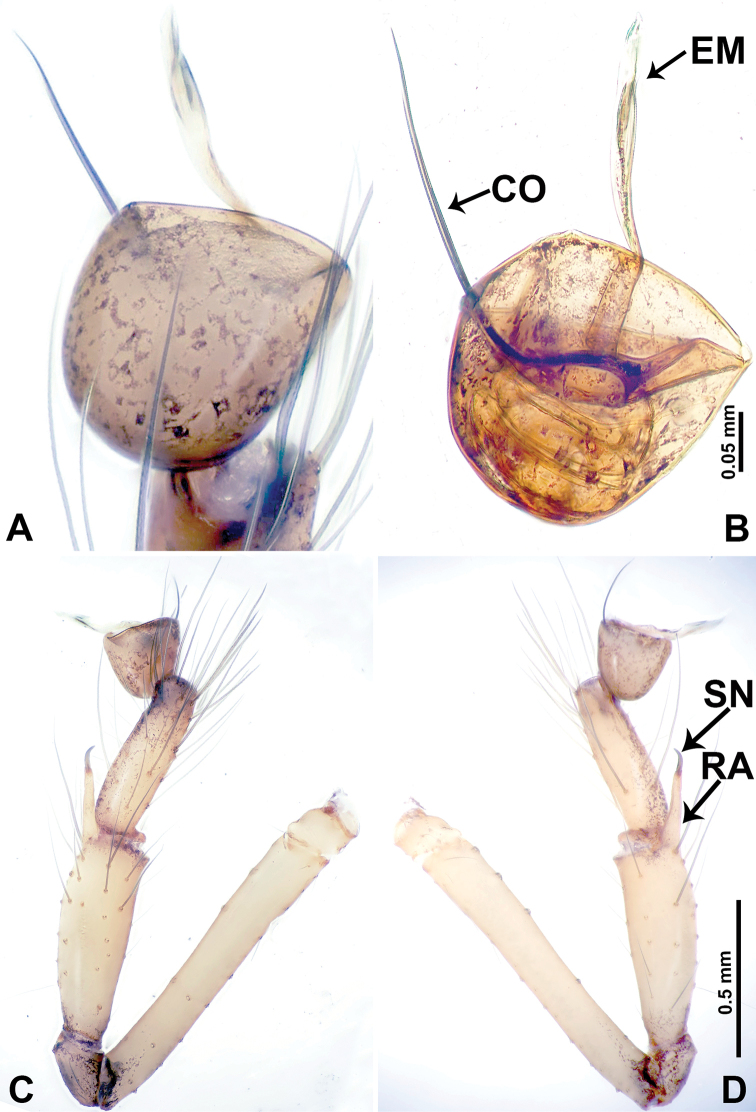
*Leclercera zhamensis* sp. nov. **A** Palp, ventral view **B** bulb, ventral view **C** palp, prolateral view **D** palp, retrolateral view. Abbreviations: CO = conductor, EM = embolus, RA = retrolateral apophysis, SN = spine.

Figs 33 , 34 , 55B , 58

##### Types.

***Holotype***: ♂ (IZCAS), China, Tibet Autonomous Region, Xigaze, Nyalam County, Zham Town, 27°59.0250'N, 85°58.9720'E, elevation ca 2450 m, 29.VIII.2014, Y. Li leg.

##### Etymology.

The species name is an adjective referring to the type locality.

##### Diagnosis.

Males of *L. zhamensis* sp. nov. can be distinguished from congeners by the laminar-shaped embolus that is basally fused with the conductor (Fig. 34B) (vs. the absence of a basally fused conductor and embolus in congeners); the presence of a retrolateral apophysis on the tibia with a spine four times shorter than the apophysis (Fig. 34D) (vs. the absence of a spine with such characteristics in congeners).

##### Description.

**Male** (Holotype). Total length 3.84; carapace 1.28 long, 1.25 wide; abdomen 2.56 long, 0.88 wide. Carapace round and brown, with three dark brown longitudinal bands, median band 3 times wider than lateral bands (Fig. 33A). Chelicerae brown (Fig. 55B). Clypeus brown, with dark brown band medially. Endites dark brown, light brown basally. Labium and sternum dark brown. Abdomen elongated, antero-dorsally with three pairs of dark brown spots laterally, pale brown medially, posterior with dark brown stripes medially, antero-ventrally with dark brown patch, and a pair of longitudinal dark brown bands laterally, with dark brown posterior patches laterally. Legs uniformly brown; measurements: I 24.21 (7.05, 0.50, 7.37, 7.05, 2.24), II 17.81 (5.13, 0.50, 5.13, 5.13, 1.92), III 11.53 (3.53, 0.40, 3.00, 3.00, 1.60), IV 17.32 (4.81, 0.50, 5.13, 5.13, 1.75). Palp (Fig. 34A–D): femur slender, six times longer than patella; patella not swollen; tibia slightly swollen anteriorly, 1.5 times shorter than femur, with an anterior retrolateral apophysis bearing a spine four times shorter than the apophysis (Fig. 34D); cymbium dark brown anteriorly, 2.5 times shorter than femur; bulb brown, semicircle with conductor and embolus arising distally; conductor dark and thin, basally fused with embolus, almost as long as embolus, two times longer than tegulum; embolus laminar-like, almost transparent (Fig. 34B).

**Female**. Unknown.

##### Distribution.

Known only from the type locality (Fig. 58).

#### Leclercera sanjiao sp. nov.

Taxon classificationAnimaliaAraneaePsilodercidae

5A3D9631-C299-53E8-81E3-FA75A15016E2

http://zoobank.org/F7DF65E5-B728-4856-A17F-2BDD1D4B4C0D

Figs 35 , 55C , 58

##### Types.

***Holotype***: ♀ (IZCAS), China, Tibet Autonomous Region, Xigaze, Gyirong County, Zalong Village, 28°22.8650'N, 85°21.1580'E, elevation ca 2715 m, 31.VIII.2014, Y. Li leg.

##### Etymology.

The species name is a noun in apposition derived from the Chinese pinyin “sānjiǎo” (triangle) and refers to the distinct triangular shape of the external genitalia.

##### Diagnosis.

**Figure 35.**
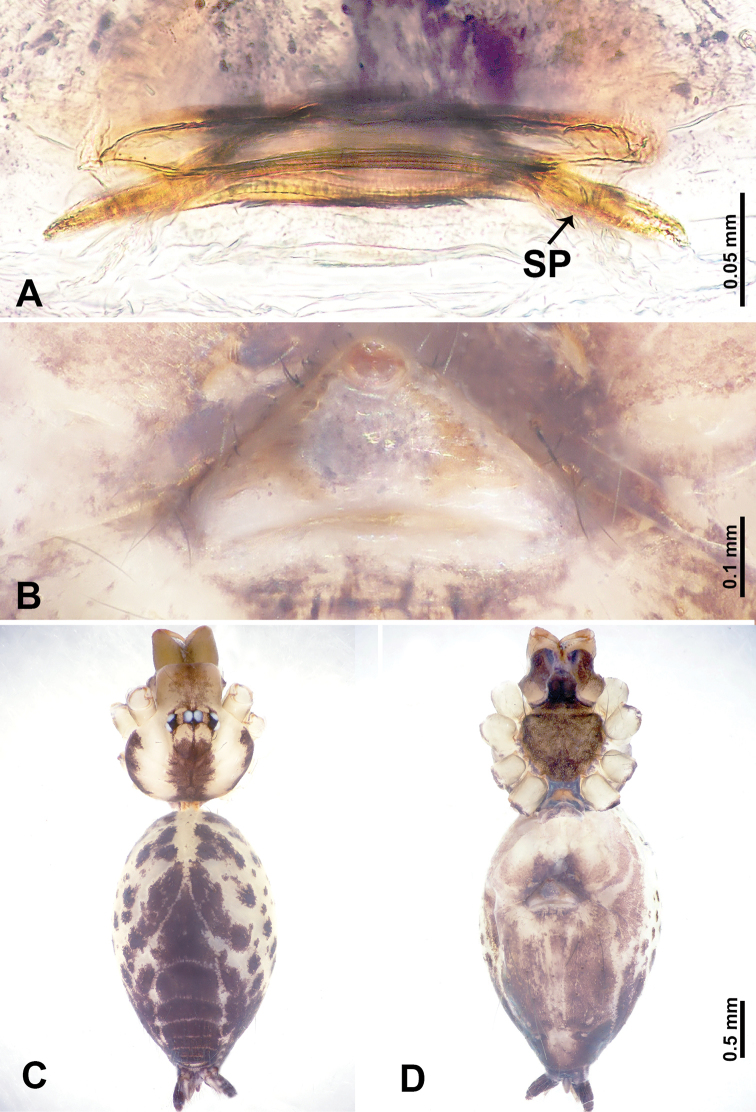
*Leclercera sanjiao* sp. nov., female paratype. **A** Endogyne, dorsal view **B** female epigastric area, ventral view **C** female habitus, dorsal view **D** female habitus, ventral view. Abbreviation: SP = spermatheca.

Females of *L. sanjiao* sp. nov. can be distinguished from other congeners by the distinct triangular form of the external genitalia (Fig. 35B) (vs. the absence of triangular external genitalia in congeners), and a pair of transverse linear spermathecae (Fig. 35A) (vs. the absence of linear spermathecae in congeners).

##### Description.

**Female.** Total length 3.80; carapace 1.20 long, 1.20 wide; abdomen 2.60 long, 1.60 wide. Carapace round and brown, with three longitudinal dark brown bands, median band four times wider than the lateral band (Fig. 35C). Chelicerae brown (Fig. 55C). Clypeus brown, with dark brown band medially. Endites dark brown, light brown basally. Labium and sternum dark brown. Abdomen elongated, dorsum with scattered dark brown spots laterally, with dark brown median stripes (Fig. 35C), antero-ventrally light brown with a distinct triangular external genitalia region, with indistinct dark brown patches posteriorly (Fig. 35D). Legs uniformly brown; measurements: I 17.49 (4.81, 0.50, 5.13, 5.13, 1.92), II 12.90 (3.80, 0.50, 3.60, 3.60, 1.40), III 9.17 (2.88, 0.40, 2.40, 2.40, 1.09), IV 13.67 (4.17, 0.50, 3.75, 3.75, 1.50). Epigastric area (Fig. 35B): triangular pale brown patch with posterior yellowish slit. Endogyne (Fig. 35A): a pair of transverse, linear spermathecae, with the posterior pair curving downwards, slightly longer than the anterior pair; both anterior and posterior pairs with rounded tips and similar in width.

**Male**. Unknown.

##### Distribution.

Known only from the type locality (Fig. 58).

#### Leclercera selasihensis sp. nov.

Taxon classificationAnimaliaAraneaePsilodercidae

2FACDF47-236F-54D1-8026-E10E3EC58CA7

http://zoobank.org/67E75A89-0332-460F-8968-FC6D89E72FA8

**Figure 36.**
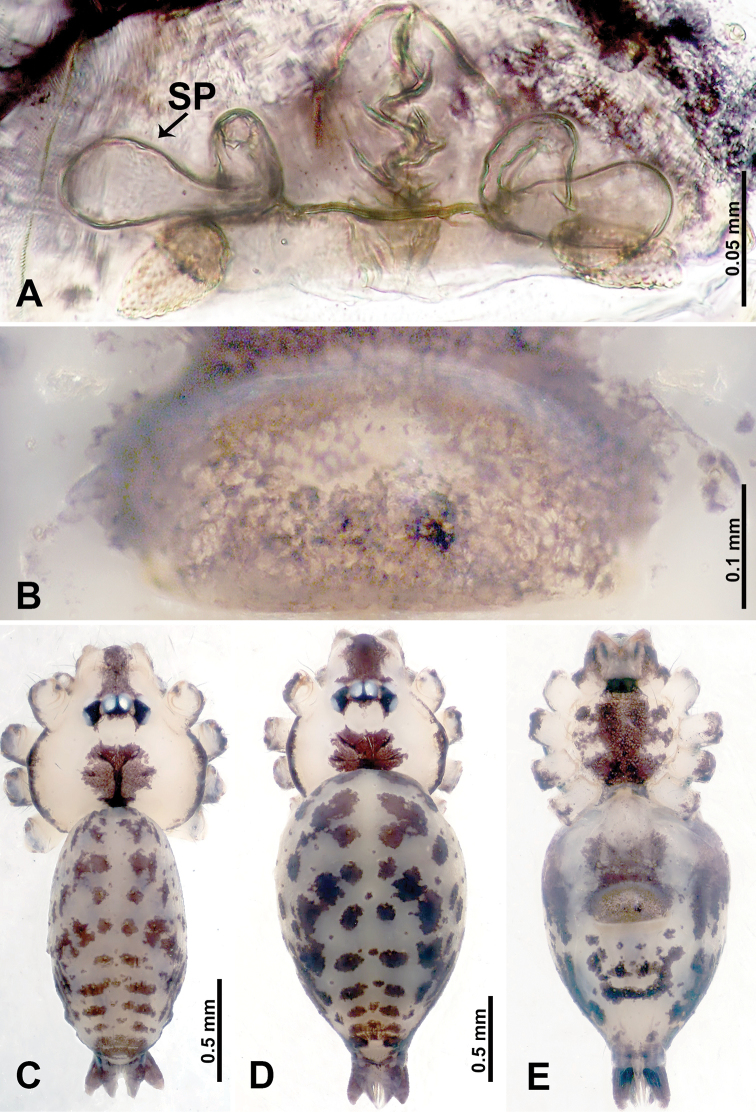
*Leclercera selasihensis* sp. nov., male holotype and female paratype. **A** Endogyne, dorsal view **B** female epigastric area, ventral view **C** male habitus, dorsal view **D** female habitus, dorsal view **E** female habitus, ventral view. Abbreviation: SP = spermatheca.

**Figure 37.**
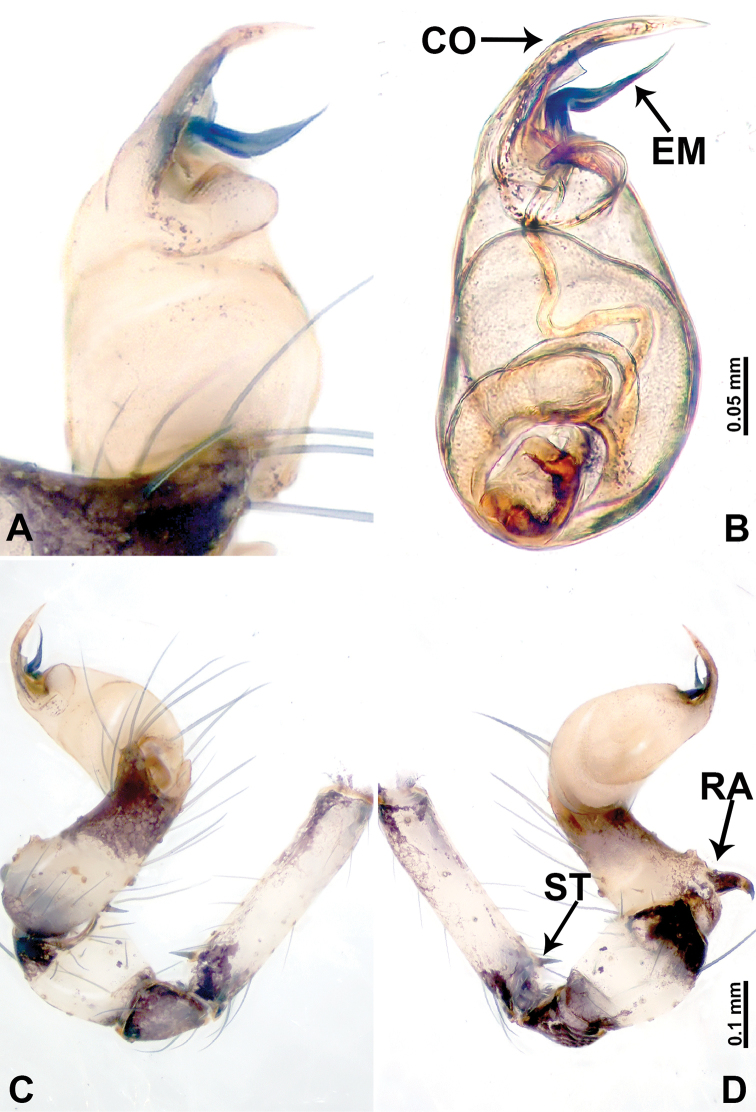
*Leclercera selasihensis* sp. nov. **A** Palp, ventral view **B** bulb, ventral view **C** palp, prolateral view **D** palp, retrolateral view. Abbreviations: CO = conductor, EM = embolus, RA = retrolateral apophysis, ST = strong seta.

Figs 36 , 37 , 56I , 58

##### Types.

***Holotype***: ♂ (IZCAS), Indonesia, Sumatra, West Sumatra Province, Solok Bukit Selasih Village, 0°46.0400'S, 100°43.1750'E, elevation ca 426 m, 28.V.2014, Z. Yao leg. ***Paratype***: 1♀ (IZCAS), same data as holotype.

##### Etymology.

The species name is an adjective referring to the type locality.

##### Diagnosis.

Diagnostic features of males and females are discussed in the diagnosis of *L. mianqiu* sp. nov.

##### Description.

**Male** (Holotype). Total length 1.80; carapace 0.70 long, 0.75 wide; abdomen 1.10 long, 0.63 wide. Carapace round and pale brown, with three longitudinal dark brown bands, median band six times wider than lateral band (Fig. 36C). Chelicerae dark brown (Fig. 56I). Clypeus pale brown, dark brown band medially. Endites dark brown, light brown basally. Labium dark brown. Sternum dark brown, with lateral pale brown spots. Abdomen elongated, dorsum with complex dark brown spots, antero-ventrally with “V”-shaped dark brown patch and brown ovoid patch, posterior with brown spots forming a ring shape. Legs uniformly brown; measurements: I missing, II 8.43 (2.50, 0.31, 2.34, 2.50, 0.78), III 6.52 (1.88, 0.25, 1.88, 1.88, 0.63), IV 9.56 (2.75, 0.25, 2.81, 2.97, 0.78). Palp (Fig. 37A–D): femur slender, four times longer than patella, anterior with one strong seta prolaterally; patella not swollen, dark purplish; tibia swollen, 1.2 times shorter and two times wider than femur, dark purplish proximally and distally; cymbium two times shorter than femur, dark purplish distally, basally swollen with slightly curved postero-retrolateral apophysis that is almost perpendicular to cymbium (Fig. 37D); bulb light brown, pyriform, with embolus and conductor arising distally; embolus thin and dark, arises medially from tegulum, half the length of, and adjacent to, conductor; conductor arises laterally from tegulum, two times shorter than tegulum, attached with tiny triangular protrusion (Fig. 37B).

**Female** (Paratype). General features and coloration similar to those of male (Fig. 36D, E). Measurements: total length 1.80; carapace 0.60 long, 0.70 wide; abdomen 1.20 long, 0.86 wide. Leg measurements: I missing, II 6.76 (1.80, 0.25, 1.88, 2.03, 0.80), III 5.10 (1.50, 0.20, 1.30, 1.50, 0.60), IV 7.60 (2.03, 0.20, 2.25, 2.34, 0.78). Epigastric area (Fig. 36B): an elliptical dark brown patch. Endogyne (Fig. 36A): a pair of bean-shaped spermathecae with a strong depression and rounded ends, spiralled duct system medially.

##### Distribution.

Known only from the type locality (Fig. 58).

#### Leclercera yuanzhui sp. nov.

Taxon classificationAnimaliaAraneaePsilodercidae

83B1D618-77FE-530B-9A51-43706481006D

http://zoobank.org/F1620E22-6F83-4C7D-88D7-F5BECD248BB9

Figs 38 , 56J , 58

##### Types.

***Holotype***: ♀ (IZCAS), China, Yunnan Province, Wenshan State, Jinping County, mountain around bus station (direction of timber factory), 22°47.6920'N, 103°13.3360'E, elevation ca 1399 m, 14.V.2015, Z. Chen and F. Li leg.

##### Etymology.

**Figure 38.**
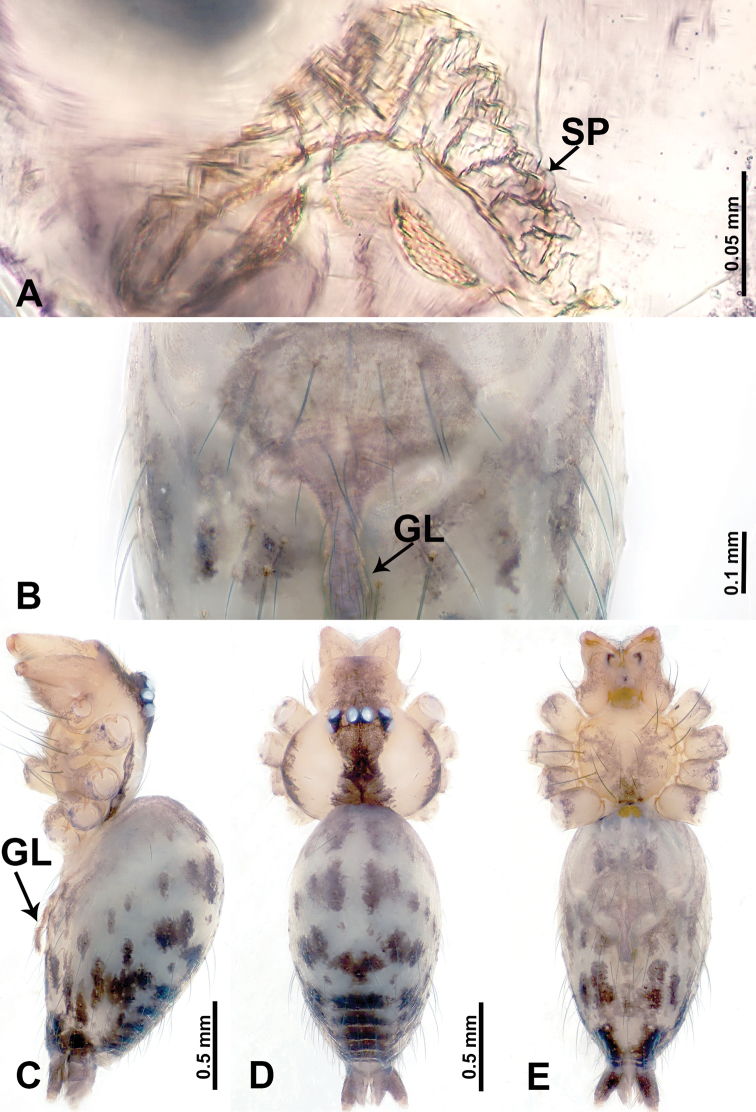
*Leclercera yuanzhui* sp. nov., female paratype. **A** Endogyne, dorsal view **B** female epigastric area, ventral view **C** female habistus, lateral view **D** female habitus, dorsal view **E** female habitus, ventral view. Abbreviations: GL = genitalic lobe, SP = spermatheca.

The species name is a noun in apposition derived from the Chinese pinyin “yuánzhuī” (conical) and refers to the shape of the external genitalia with a genitalic lobe that greatly resembles the shape of a conical flask (Fig. 38B, C).

##### Diagnosis.

Females of *L. yuanzhui* sp. nov. resemble *L. maochong* sp. nov. and *L. shanzi* sp. nov. but can be distinguished by the presence of a protruded genitalic lobe (Fig. 38C) (vs. the absence of a genitalic lobe), the presence of a pair of widely separated semi-circular bodies posteriorly (vs. the absence of semi-circular bodies or with circular bodies), and entire spermathecae rather thin and narrow (Fig. 38A) (vs. entire spermathecae rather thick and wide).

##### Description.

**Female.** Total length 2.20; carapace 0.70 long, 0.90 wide; abdomen 1.50 long, 0.90 wide. Carapace round and brown, with three dark brown longitudinal bands, median band six times wider than lateral band (Fig. 38D). Chelicerae brown (Fig. 56J). Clypeus dark brown medially. Endites brown, light brown basally. Labium brown. Sternum with purplish stripes laterally. Abdomen elongated, dorsum with dark brown spots, posteriorly with dark brown stripes (Fig. 38D), antero-ventrally with conical shaped external genitalia region, with protruded genitalic lobe (Fig. 38C), posterior with complex pattern of dark brown spots. Legs uniformly brown; measurements: I–II missing, III 4.91 (1.41, 0.20, 1.40, 1.20, 0.70), IV 8.08 (2.40, 0.31, 2.40, 2.03, 0.94). Epigastric area (Fig. 38B): inverted triangular brown patch sagging with distinct genitalic lobe. Endogyne (Fig. 38A): pair of spermathecae resembling inverted “V” with loops, posteriorly with a pair of semi-circular bodies.

**Male**. Unknown.

##### Distribution.

Known only from the type locality (Fig. 58).

#### Leclercera paiensis sp. nov.

Taxon classificationAnimaliaAraneaePsilodercidae

73AB1EED-3D47-5C72-AC48-AE55CCBEE6A1

http://zoobank.org/CB98BBAE-D4B3-42A6-975C-1AD98C337991

Figs 39 , 56K , 58

##### Types.

***Holotype***: ♀ (IZCAS), China, Tibet Autonomous Region, Nyingchi, Mainling County, around Pai Town (about 65km on Gangpai highway), 29°30.7020'N, 94°52.0860'E, elevation ca 3004 m, 5.VIII.2015, J. Wu leg.

##### Etymology.

The species name is an adjective referring to the type locality.

##### Diagnosis.

**Figure 39.**
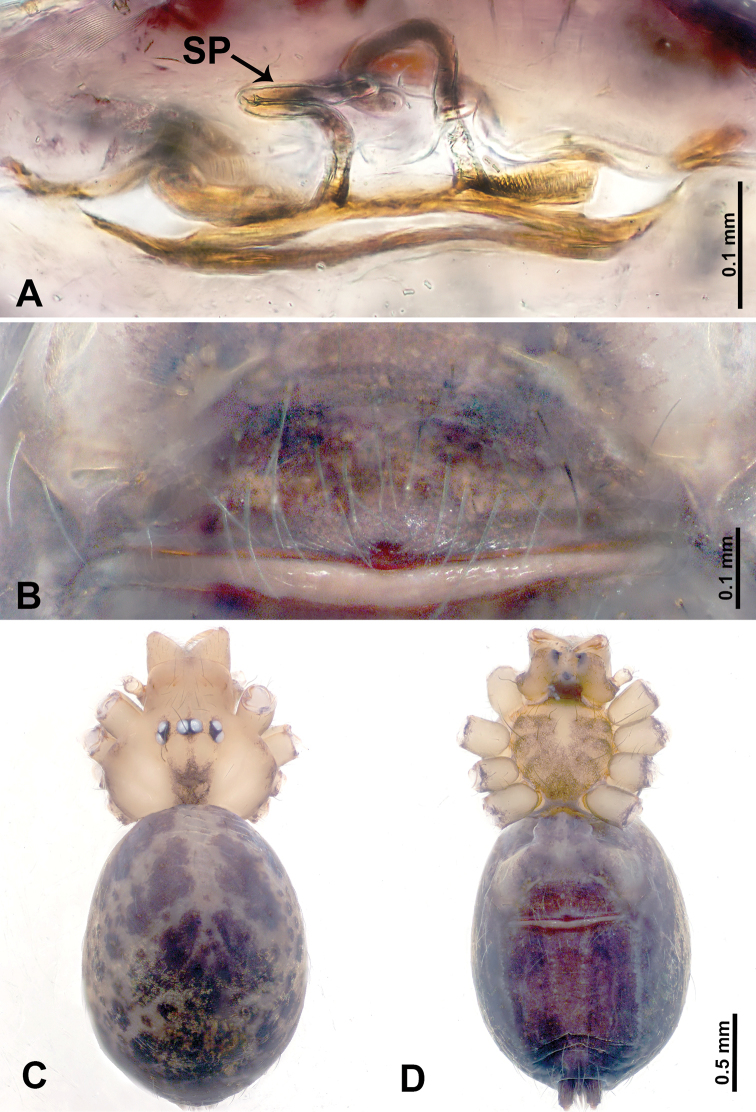
*Leclercera paiensis* sp. nov., female paratype. **A** Endogyne, dorsal view **B** female epigastric area, ventral view **C** female habitus, dorsal view **D** female habitus, ventral view. Abbreviation: SP = spermatheca.

Females of *L. paiensis* sp. nov. can be distinguished from other congeners by the presence of a distinct horizontal, thick lip posterior to the external genitalia region (Fig. 39B) (vs. the absence of a thick lip on the external genitalia region in congeners), and twisted, stalked spermathecae with ovoid bases (Fig. 39A) (vs. an absence of twisted, stalked spermathecae in congeners).

##### Description.

**Female.** Total length 3.20; carapace 1.00 long, 1.20 wide; abdomen 2.20 long, 1.56 wide. Carapace round and brown, with median dark brown band (Fig. 39C). Chelicerae brown (Fig. 56K). Clypeus light brown. Endites light brown. Labium dark brown. Sternum dark brown, delimiting light brown “T”-shape anteriorly. Abdomen elongated, dorsum with indistinct dark brown spots (Fig. 39C), antero-ventrally with a semi-circular dark brown patch followed by horizontal thick lip of external genitalia region, dark brown with complex pattern (Fig. 39D). Legs uniformly brown; measurements: I 9.74 (2.75, 0.40, 3.00, 2.34, 1.25), II 8.66 (2.34, 0.38, 2.66, 2.03, 1.25), III 6.36 (1.80, 0.31, 1.75, 1.50, 1.00), IV 9.00 (2.50, 0.40, 2.66, 2.19, 1.25). Epigastric area (Fig. 39B): dark brown, semi-circular patch with thick horizontal, light brown base posteriorly. Endogyne (Fig. 39A): a pair of twisted, stalked spermathecae with ovoid base and posteriorly connected with a wavy duct system.

**Male**. Unknown.

##### Distribution.

Known only from the type locality (Fig. 58).

#### Leclercera zanggaensis sp. nov.

Taxon classificationAnimaliaAraneaePsilodercidae

233B882A-AC4F-58FE-9DCB-9964886395EB

http://zoobank.org/459210A3-07D8-4E8A-9A3A-10A234DFF659

Figs 40 , 56L , 58

##### Types.

***Holotype***: ♀ (IZCAS), China, Tibet Autonomous Region, Xigaze, Dinggye County, Chentang Town, Zangga Village, 27°51.6307'N, 87°25.4768'E, elevation ca 2219 m, 14.VIII.2017, X. Zhang, Z. Bai leg.

##### Etymology.

The species name is an adjective referring to the type locality.

##### Diagnosis.

**Figure 40.**
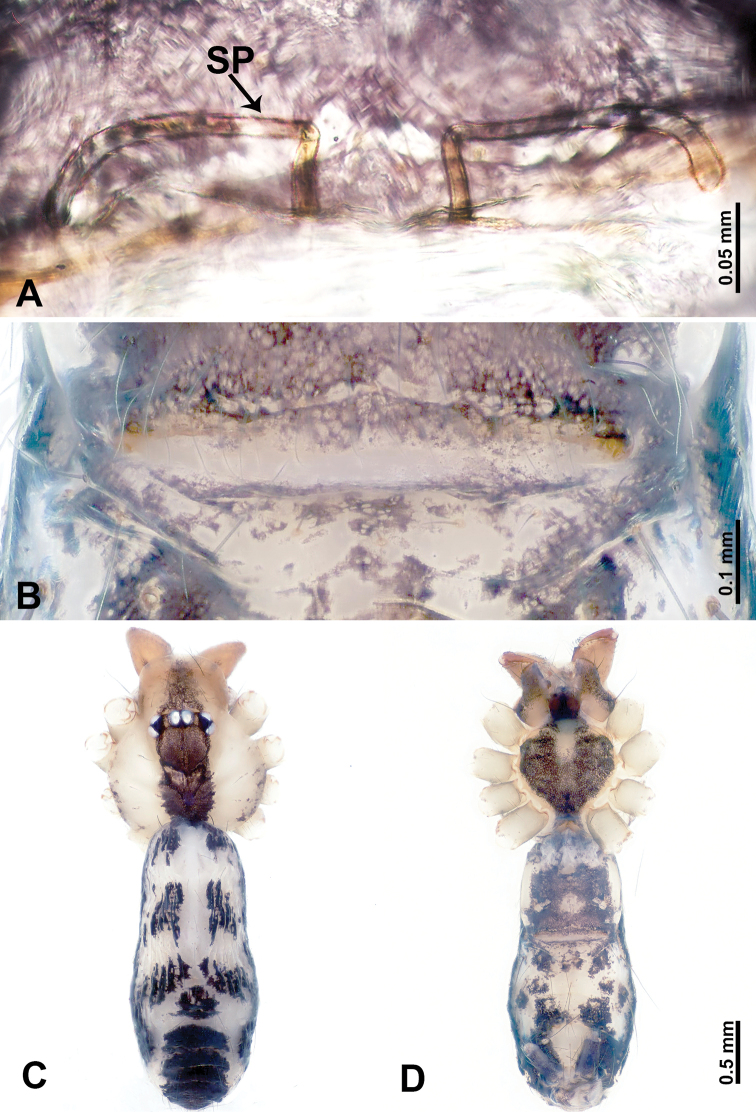
*Leclercera zanggaensis* sp. nov., female paratype. **A** Endogyne, dorsal view **B** female epigastric area, ventral view **C** female habitus, dorsal view **D** female habitus, ventral view. Abbreviation: SP = spermatheca.

Females of *L. zanggaensis* sp. nov. can be distinguished from other congeners by the presence of right-angled, stalked spermathecae (Fig. 40A) (vs. the absence of right-angled, stalked spermathecae in congeners), and the external genitalia is distinctly depressed medially (Fig. 40B) (vs. the absence of indented external genitalia in congeners).

##### Description.

**Female.** Total length 3.12; carapace 1.09 long, 1.09 wide; abdomen 2.03 long, 0.80 wide. Carapace round and brown, with median dark brown longitudinal band (Fig. 40C). Chelicerae brown (Fig. 56L). Clypeus dark brown medially. Endites dark brown, light brown basally. Labium dark brown. Sternum with dark brown heart shape. Abdomen elongated, antero-dorsum with pairs of dark brown longitudinal spots, posteriorly with dark brown horizontal stripes (Fig. 40C), antero-ventrally with elliptical external genitalia region, posterior with complex dark brown spots. Legs uniformly brown; measurements: I 13.11 (3.75, 0.31, 3.80, 3.75, 1.50), II–III missing, IV 13.40 (3.75, 0.40, 4.00, 3.75, 1.50). Epigastric area (Fig. 40B): linear light brown patch with distinct indented slit. Endogyne (Fig. 40A): a pair of right-angled, stalked spermathecae, with slightly downward curving blunt ends, vertical parts five times shorter than horizontal parts.

**Male**. Unknown.

##### Distribution.

Known only from the type locality (Fig. 58).

#### Leclercera aniensis sp. nov.

Taxon classificationAnimaliaAraneaePsilodercidae

F154C692-46C3-522E-B465-7A42F7A4F985

http://zoobank.org/62DFCEEF-797A-4766-8EEB-485CDCD80BB9

**Figure 41.**
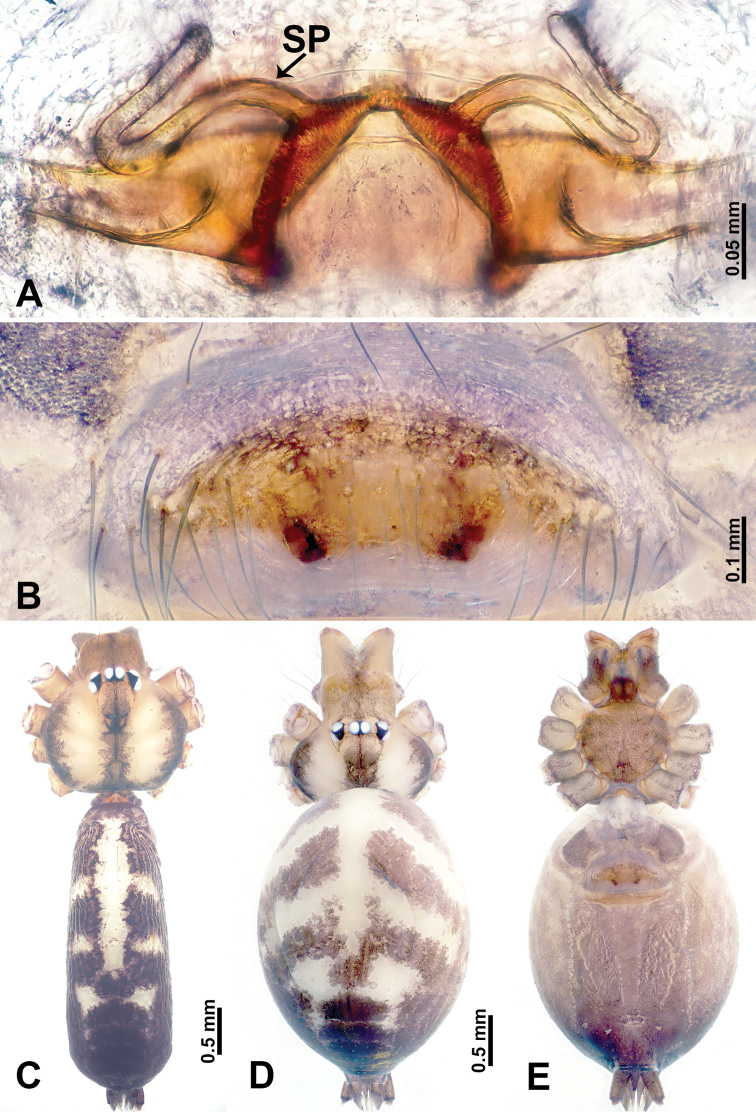
*Leclercera aniensis* sp. nov., male holotype and female paratype. **A** Endogyne, dorsal view **B** female epigastric area, ventral view **C** male habitus, dorsal view **D** female habitus, dorsal view **E** female habitus, ventral view. Abbreviation: SP = spermatheca

**Figure 42.**
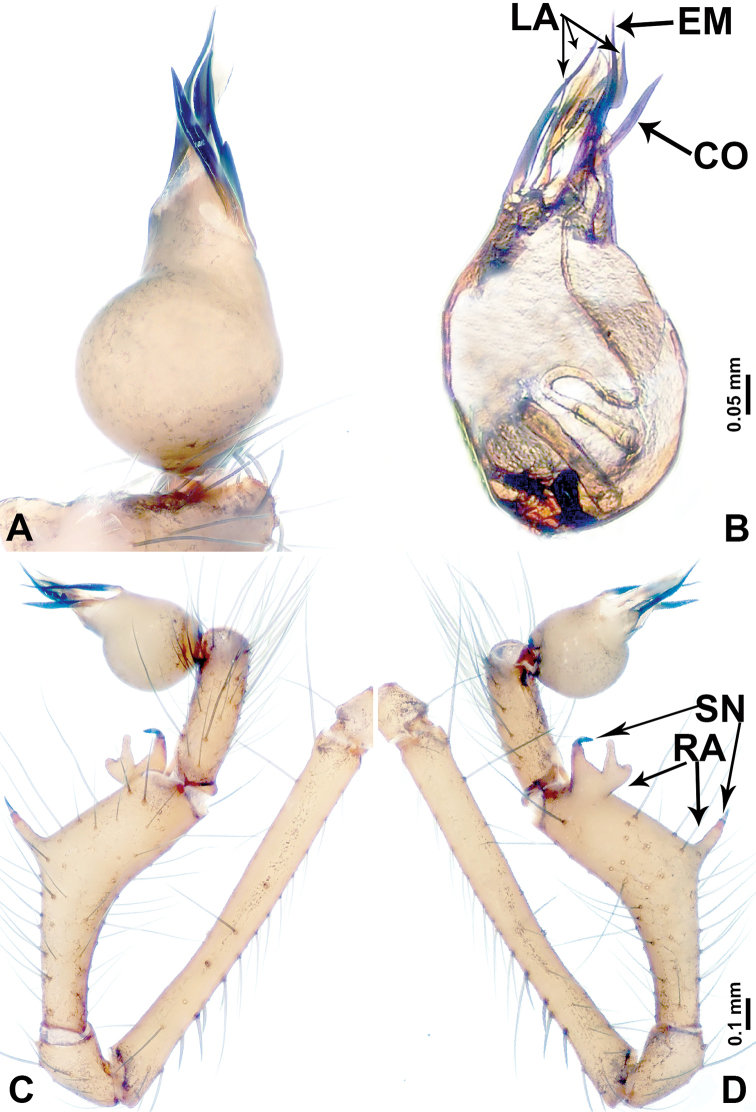
*Leclercera aniensis* sp. nov. **A** Palp, ventral view **B** bulb, ventral view **C** palp, prolateral view **D** palp, retrolateral view. Abbreviations: CO = conductor, EM = embolus, LA = laminar apophyses, RA = retrolateral apophyses, SN = spines.

Figs 41 , 42 , 57A , 58

##### Types.

***Holotype***: ♂ (IZCAS), China, Tibet Autonomous Region, Nyingchi, Medog County, Medog Town, Beibung Village, road from Jiefang Bridge to Ani Bridge, 30°11.2620'N, 94°19.4180'E, elevation ca 3087 m, 18.VII.2019, X. Zhang, Z. Bai and J. Liu leg. ***Paratype***: 1♀ (IZCAS), same data as holotype.

##### Etymology.

The species name is an adjective referring to the type locality.

##### Diagnosis.

Diagnostic features of males and females are discussed in the diagnosis of *L. jiazhongensis* sp. nov.

##### Description.

**Male** (Holotype). Total length 4.38; carapace 1.38 long, 1.50 wide; abdomen 3.00 long, 1.09 wide. Carapace round and brown, with three dark brown longitudinal bands, median band and lateral bands similar in width (Fig. 41C). Chelicerae brown (Fig. 57A). Clypeus dark brown, darkens anteriorly. Endites dark brown, light brown basally. Labium and sternum dark brown. Abdomen elongated, dorsum with three pairs of dark brown spots laterally, posterior with dark brown horizontal stripes, antero-ventrally with a pair of dark brown square spots laterally, followed by elliptical yellowish spot, posterior with indistinct purplish veined spots. Legs uniformly brown; measurements: I 18.91 (5.45, 0.64, 5.77, 5.13, 1.92), II 16.21 (4.49, 0.50, 4.81, 4.49, 1.92), III 11.50 (3.50, 0.40, 3.20, 3.00, 1.40), IV missing. Palp (Fig. 42A–D): femur slender, five times longer than patella; patella not swollen; tibia swollen, resembles isosceles triangle, with two retrolateral apophyses anteriorly and medially (Fig. 42D), anterior apophysis branched and two times wider than the median apophysis, one branch bearing a tiny crooked spine, another branch with a divided protrusion; median apophysis bearing spine three times shorter than apophysis; cymbium three times shorter than femur; bulb brown, pyriform, with embolus, laminar apophysis, and conductor arising distally; embolus black and slender, longest between laminar apophyses and conductor; three laminar apophyses (middle apophysis three times wider than the other two) adjacent to embolus; conductor slightly similar in width to, shorter than, and basally connected to embolus (Fig. 42B).

**Female** (Paratype). General features and coloration similar to those of male (Fig. 41D, E). Measurements: total length 3.44; carapace 0.94 long, 1.25 wide; abdomen 2.50 long, 1.80 wide. Leg measurements: I 11.73 (3.21, 0.40, 3.50, 3.21, 1.41), II 8.44 (2.40, 0.40, 2.25, 2.19, 1.20), III missing, IV 11.70 (3.40, 0.40, 3.40, 3.00, 1.50). Epigastric area (Fig. 41B): semi-circular patch, outer region pale purple and inner region brownish, with a pair of dark brown dots. Endogyne (Fig. 41A): pair of slightly twisted, upturned, stalked spermathecae with isosceles triangle-shaped receptacles, upturned spermathecae with blunt tips.

##### Distribution.

Known only from the type locality (Fig. 58).

#### Leclercera renqinensis sp. nov.

Taxon classificationAnimaliaAraneaePsilodercidae

9CEC64B8-E32A-5292-99D8-5D7C2B711BF7

http://zoobank.org/517CC264-4972-45A5-9D2B-D8C6E5860AB6

**Figure 43.**
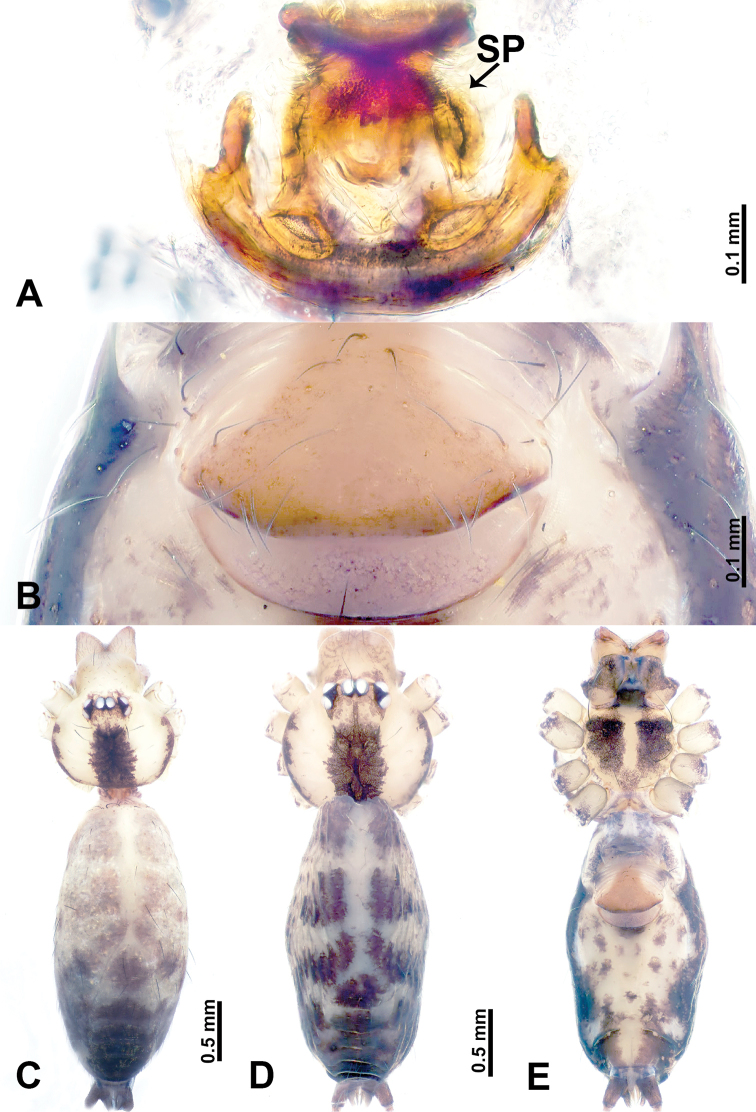
*Leclercera renqinensis* sp. nov., male holotype and female paratype. **A** Endogyne, dorsal view **B** female epigastric area, ventral view **C** male habitus, dorsal view **D** female habitus, dorsal view **E** female habitus, ventral view. Abbreviation: SP = spermatheca.

**Figure 44.**
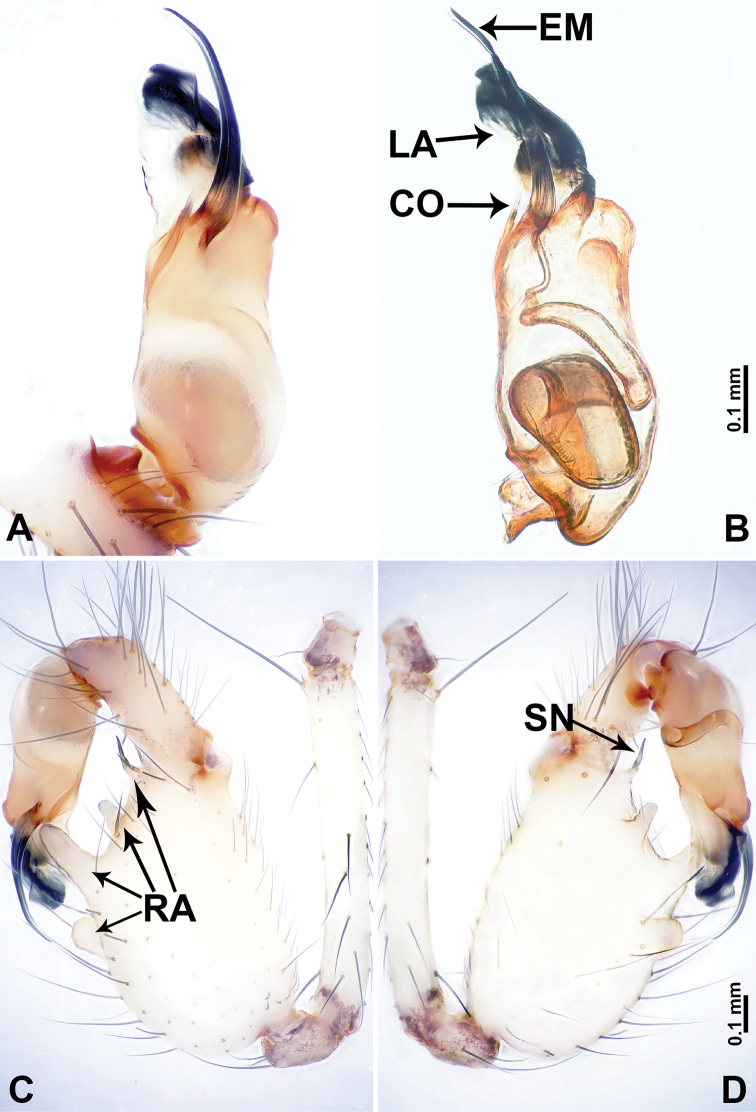
*Leclercera renqinensis* sp. nov. **A** Palp, ventral view **B** bulb, ventral view **C** palp, prolateral view **D** palp, retrolateral view. Abbreviations: CO = conductor, EM = embolus, LA = laminar apophysis, RA = retrolateral apophyses, SN = spine.

Figs 43 , 44 , 57B , 58

##### Types.

***Holotype***: ♂ (IZCAS), China, Tibet Autonomous Region, Nyingchi, Medog County, Medog Town, road to Renqinbeng Temple, 29°18.4260'N, 95°21.5100'E, elevation ca 2036 m, 21.VII.2019, X. Zhang, Z. Bai and J. Liu. ***Paratype***: 1♀ (IZCAS), same data as holotype.

##### Etymology.

The species name is an adjective referring to the type locality.

##### Diagnosis.

Males of *L. renqinensis* sp. nov. resemble *L. tudao* sp. nov. but can be distinguished by the presence of four uneven retrolateral apophyses on a swollen tibia, with one apophysis bearing a spine (Fig. 44C) (vs. the presence of a spine and three retrolateral apophyses bearing spines on a swollen tibia (Fig. 50C)), tibia swollen pentagonally (Fig. 44C) (vs. tibia swollen triangularly (Fig. 50C)), pyriform bulb rather slender and elongated (Fig. 44B) (vs. pyriform bulb rather plump (Fig. 50B)), a thin strip of the conductor arises at the margins (Fig. 44B) (vs. a wide part of the conductor arises at the margins), laminar apophysis and embolus not attached to each other (Fig. 44B) (vs. embolus attached to laminar apophysis); females can be recognized by having a pair of spermathecae resembling a bow (Fig. 43A) (vs. a pair of stalked spermathecae (Fig. 49A)), external genitalia with triangular orange area (Fig. 43B) (vs. dark brown area (Fig. 49B)).

##### Description.

**Male** (Holotype). Total length 3.30; carapace 0.96 long, 1.09 wide; abdomen 2.34 long, 1.25 wide. Carapace round and brown, with three dark brown longitudinal bands, median band six times wider than lateral band (Fig. 43C). Chelicerae brown (Fig. 57B). Clypeus pale brown. Endites dark brown, light brown basally. Labium dark brown. Sternum dark brown laterally, light brown medially. Abdomen elongated, dorsum with brown spots, posterior with dark brown horizontal stripes, antero-ventrally with distinct triangular orange area, posterior with dark brown bands laterally, scattered brown spots medially. Legs uniformly brown; measurements: I 16.00 (4.25, 0.40, 4.75, 5.00, 1.60), II 11.61 (3.21, 0.40, 3.20, 3.40, 1.40), III 7.71 (2.20, 0.31, 2.20, 2.00, 1.00), IV 12.50 (3.85, 0.40, 3.50, 3.50, 1.25). Palp (Fig. 44A–D): femur slender, five times longer than patella; patella not swollen; tibia swollen pentagonally, 1.2 times shorter and 4 times wider than femur, with four retrolateral apophyses anteriorly (Fig. 44C) (one apophysis bearing a spine, three others with rounded tip, with apophysis one being longest); cymbium three times shorter than femur, brown; bulb brown, pyriform, with embolus, laminar apophysis, and conductor arising distally; embolus black and slender, longer than laminar apophysis and conductor; laminar apophysis with blunt tip, two times wider and not attached to embolus; conductor brown, arising marginally as a thin strip, embolus three times wider and longer than conductor (Fig. 44B).

**Female** (Paratype). General features and coloration similar to those of male (Fig. 43D, E). Measurements: total length 2.82; carapace 0.94 long, 1.00 wide; abdomen 1.88 long, 1.00 wide. Leg measurements: I 12.65 (2.75, 0.40, 4.00, 4.00, 1.50), II 10.11 (2.80, 0.31, 3.00, 2.80, 1.20), III 9.06 (1.75, 0.31, 3.00, 2.80, 1.20), IV 10.80 (3.00, 0.40, 3.20, 3.00, 1.20). Epigastric area (Fig. 43B): distinct triangular orangeish patch with pale brown crescent at base. Endogyne (Fig. 43A): spermathecae resemble a ribbon knot with a pair of ovoid droplet-shaped marks, receptacles with rounded upturned ends.

##### Distribution.

Known only from the type locality (Fig. 58).

#### Leclercera shergylaensis sp. nov.

Taxon classificationAnimaliaAraneaePsilodercidae

C25A5D32-0D82-56BA-A313-FBA2441EEEAA

http://zoobank.org/5D098902-6A00-480F-91C4-7706C93EECEE

**Figure 45.**
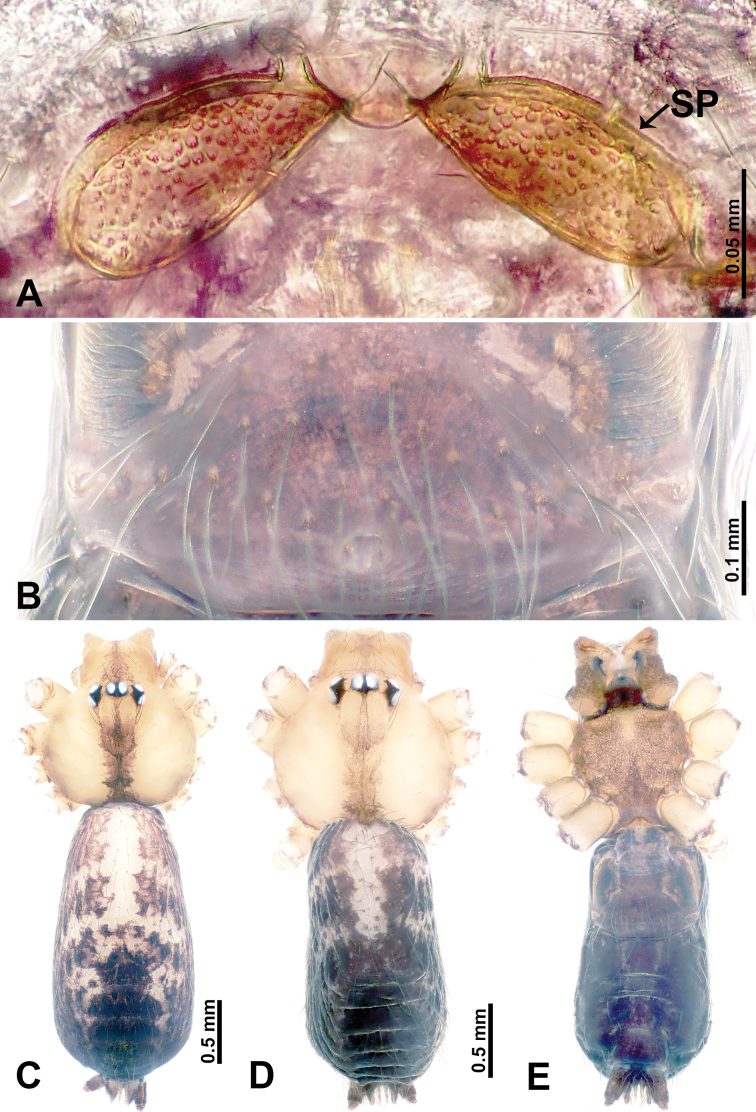
*Leclercera shergylaensis* sp. nov., male holotype and female paratype. **A** Endogyne, dorsal view **B** female epigastric area, ventral view **C** male habitus, dorsal view **D** female habitus, dorsal view **E** female habitus, ventral view. Abbreviation: SP = spermatheca.

**Figure 46.**
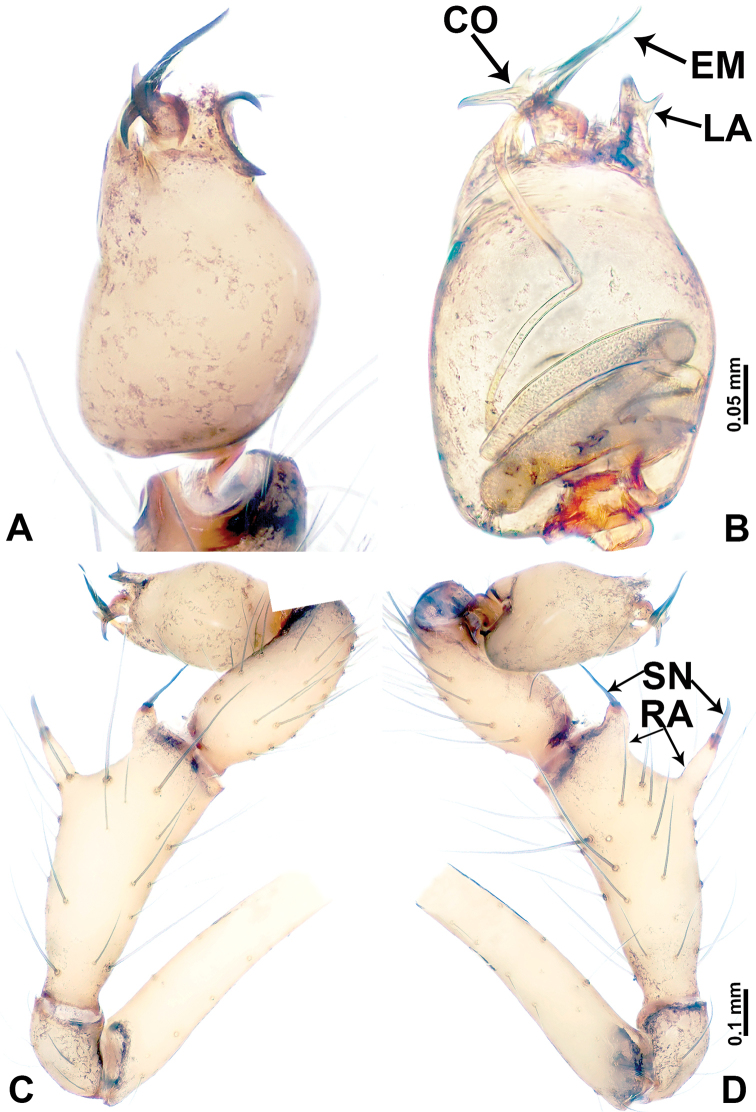
*Leclercera shergylaensis* sp. nov. **A** Palp, ventral view **B** bulb, ventral view **C** palp, prolateral view **D** palp, retrolateral view. Abbreviations: CO = conductor, EM = embolus, LA = laminar apophysis, RA = retrolateral apophyses, SN = spines.

Figs 45 , 46 , 57C , 58

##### Types.

***Holotype***: ♂ (IZCAS), China, Tibet Autonomous Region, Nyingchi, Shergyla Mountain, 29°33.7980'N, 94°34.2060'E, elevation ca 3764 m, 15.VII.2019, X. Zhang, Z. Bai and J. Liu leg. ***Paratype***: 1♀ (IZCAS), same data as holotype.

##### Etymology.

The species name is an adjective referring to the type locality.

##### Diagnosis.

Males of *L. shergylaensis* sp. nov. resemble *L. pulongensis* sp. nov. and *L. duibaensis* sp. nov. but can be distinguished by the presence of a conductor (Fig. 46B) (vs. absence of a conductor in *L. pulongensis* sp. nov. (Fig. 48B) and *L. duibaensis* sp. nov. (Fig. 52B)), two retrolateral apophyses bearing a spine on the tibia (Fig. 46D) (vs. a prolateral apophysis bearing a hooked spine on the tibia in *L. pulongensis* sp. nov. (Fig. 48C)) and a retrolateral apophysis bearing spine on tibia in *L. duibaensis* sp. nov. (Fig. 52D)), absence of strong setae in both *L. shergylaensis* sp. nov. and *L. duibaensis* sp. nov. (vs. the presence of strong setae on the tibia anteriorly in *L. pulongensis* sp. nov. (Fig. 48D)), and the absence of a bulge on the bulb of both *L. shergylaensis* sp. nov. and *L. pulongensis* sp. nov. (vs. the presence of a bulge on the bulb of *L. duibaensis* sp. nov. (Fig. 52B)), absence of an apophysis on the cymbium in both *L. shergylaensis* sp. nov. and *L. pulongensis* sp. nov. (vs. the presence of a retrolateral apophysis bearing four spines on the cymbium in *L. duibaensis* sp. nov. (Fig. 52D)), and the tibia swollen angularly (Fig. 46C) (vs. tibia roundly swollen in both *L. pulongensis* sp. nov. and *L. duibaensis* sp. nov.).

##### Description.

**Male** (Holotype). Total length 3.13; carapace 1.00 long, 1.13 wide; abdomen 2.13 long, 1.09 wide. Carapace round and brown, with median dark brown band (Fig. 45C). Chelicerae brown (Fig. 57C). Clypeus pale brown. Endites dark brown, light brown basally. Labium dark brown. Sternum dark brown, with short light brown band medially. Abdomen elongated, dorsum with dark brown spots, posterior with dark brown horizontal stripes, antero-ventrally with dark brown inverted fan-shaped pattern medially and kidney-shaped pattern laterally, posterior dark brown with two light vertical traces medially. Legs uniformly brown; measurements: I 9.60 (2.60. 0.40. 3.00, 2.40, 1.20), II 8.94 (2.60, 0.40, 2.50, 2.24, 1.20), III missing, IV 9.20 (2.60, 0.40, 2.80, 2.20, 1.20). Palp (Fig. 46A–D): femur slender, four times longer than patella; patella not swollen; tibia swollen pentagonally, similar length to, and 2 times wider than femur, with two retrolateral apophyses bearing spine (Fig. 46D) (one apophysis on anterior, the other medially on tibia), spine and apophysis similar in length; cymbium two times shorter than femur, dark brown distally; bulb brown, ovate, with embolus, laminar apophysis, and conductor arising distally; embolus thin, two times longer than conductor and laminar apophysis; branched laminar apophysis, two times wider than embolus, adjacent to conductor; conductor branched, with the longer branch longer than laminar apophysis, attached to embolus (Fig. 46B).

**Female** (Paratype). General features and coloration similar to those of male (Fig. 45D, E). Measurements: total length 2.44; carapace 0.94 long, 1.00 wide; abdomen 1.50 long, 0.88 wide. Leg measurements: I 7.69 (2.24, 0.31, 2.34, 1.80, 1.00), II 6.70 (1.80, 0.31, 2.03, 1.56, 1.00), III 5.29 (1.41, 0.31, 1.38, 1.25, 0.94), IV 6.10 (1.80, 0.31, 2.19, 1.80, 1.00). Epigastric area (Fig. 45B): elliptical dark brown patch with distinct whitish slit. Endogyne (Fig. 45A): a pair of ovoid spermathecae, thickening at margins.

##### Distribution.

Known only from the type locality (Fig. 58).

#### Leclercera pulongensis sp. nov.

Taxon classificationAnimaliaAraneaePsilodercidae

AEE24768-4965-50A2-A5A3-653C4A6A7DBF

http://zoobank.org/7D2D5BA0-C547-4B26-A3E3-1A7A12FE7C26

**Figure 47.**
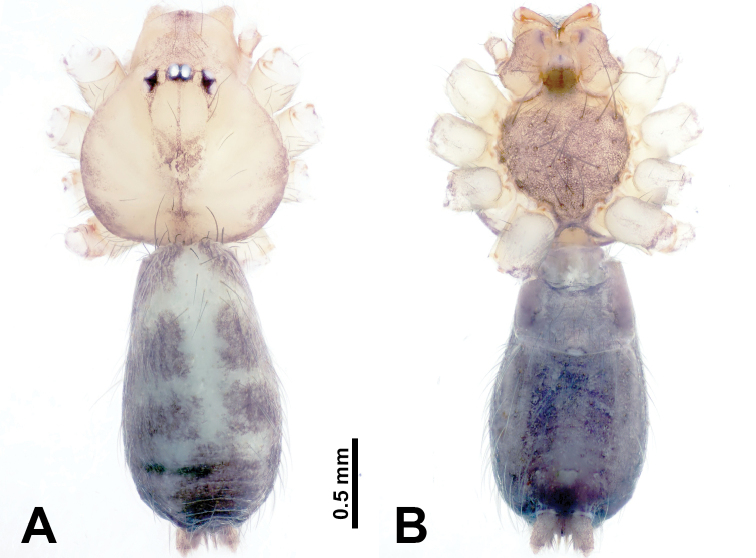
*Leclercera pulongensis* sp. nov., male holotype. **A** Habitus, dorsal view **B** habitus ventral view.

**Figure 48.**
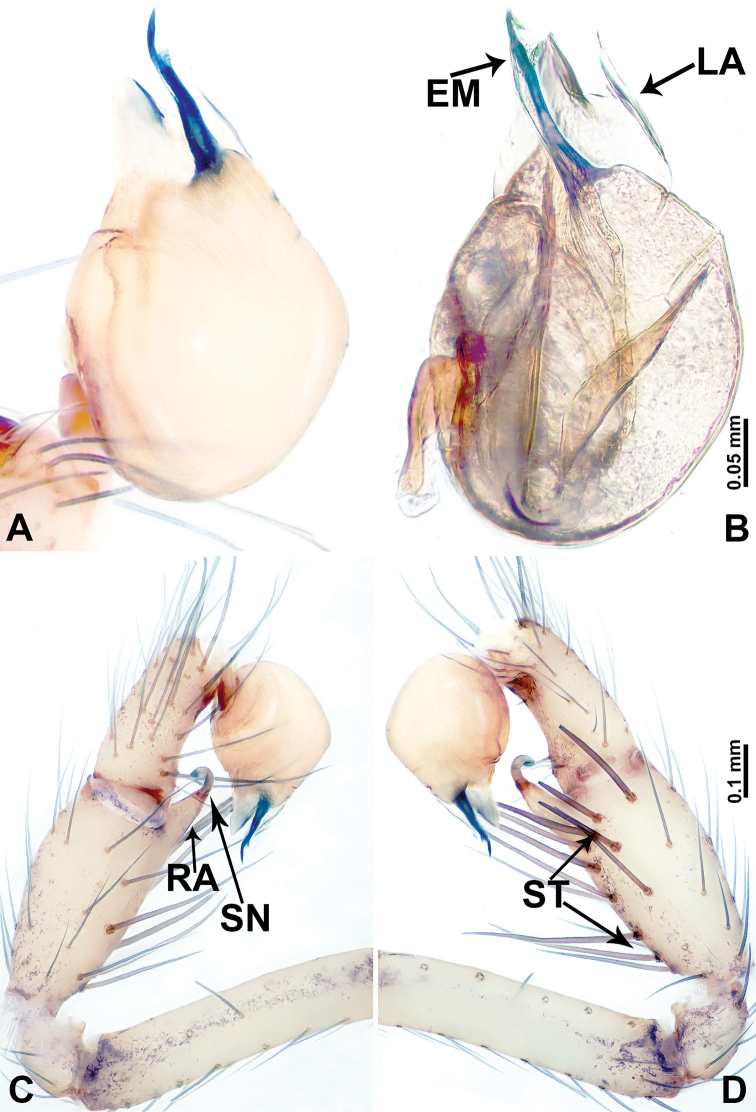
*Leclercera pulongensis* sp. nov. **A** Palp, ventral view **B** bulb, ventral view **C** palp, prolateral view **D** palp, retrolateral view. Abbreviations: EM = embolus, LA = laminar apophysis, RA = retrolateral apophysis, SN = spine, ST = strong setae.

Figs 47 , 48 , 57D , 58

##### Types.

***Holotype***: ♂ (IZCAS), China, Tibet Autonomous Region, Nyingchi, Mainling County, Pulong Village, 29°16.0980'N, 93°32.5380'E, elevation ca 3335 m, 10.VIII.2019, X. Zhang, Z. Bai and J. Liu leg.

##### Etymology.

The species name is an adjective referring to the type locality.

##### Diagnosis.

Diagnostic features of males are discussed in the diagnosis of *L. shergylaensis* sp. nov.

##### Description.

**Male** (Holotype). Total length 2.57; carapace 0.94 long, 1.13 wide; abdomen 1.63 long, 0.90 wide. Carapace round and brown, with dark brown traces marginally and medially (Fig. 47A). Chelicerae brown (Fig. 57D). Clypeus pale brown, with brown traces medially. Endites brown, light brown basally. Labium and sternum dark brown. Abdomen elongated, antero-dorsally with three pairs of dark brown spots laterally, posterior with dark brown horizontal stripes medially, antero-ventrally with pairs of brown kidney-shaped spots laterally, medially with elliptical patch, posterior with indistinct dark brown pattern. Legs uniformly brown; measurements: I 10.00 (3.00, 0.40, 3.00, 2.40, 1.20), II 9.31 (2.75, 0.31, 2.81, 2.19, 1.25), III 6.58 (1.80, 0.40, 1.88, 1.56, 0.94), IV 9.60 (2.80, 0.40, 2.80, 2.40, 1.20). Palp (Fig. 48A–D): femur slender, five times longer than patella; patella not swollen; tibia swollen, 1.2 times shorter and two times wider than femur, with prolateral apophysis bearing a hooked spine anteriorly, and more than 10 strong setae; cymbium 2.5 times shorter than femur; bulb brown, globose, with embolus and laminar apophysis arising distally; embolus thin and black, slightly longer than, and not attached to, laminar apophysis; laminar apophysis branched, with one branch four times wider than the other (Fig. 48B).

**Female**. Unknown.

##### Distribution.

Known only from the type locality (Fig. 58).

#### Leclercera tudao sp. nov.

Taxon classificationAnimaliaAraneaePsilodercidae

1A32C045-4735-58D7-A5D9-59291239F48F

http://zoobank.org/9514B93C-82C8-4B13-9B64-1A50A42CFF68

**Figure 49.**
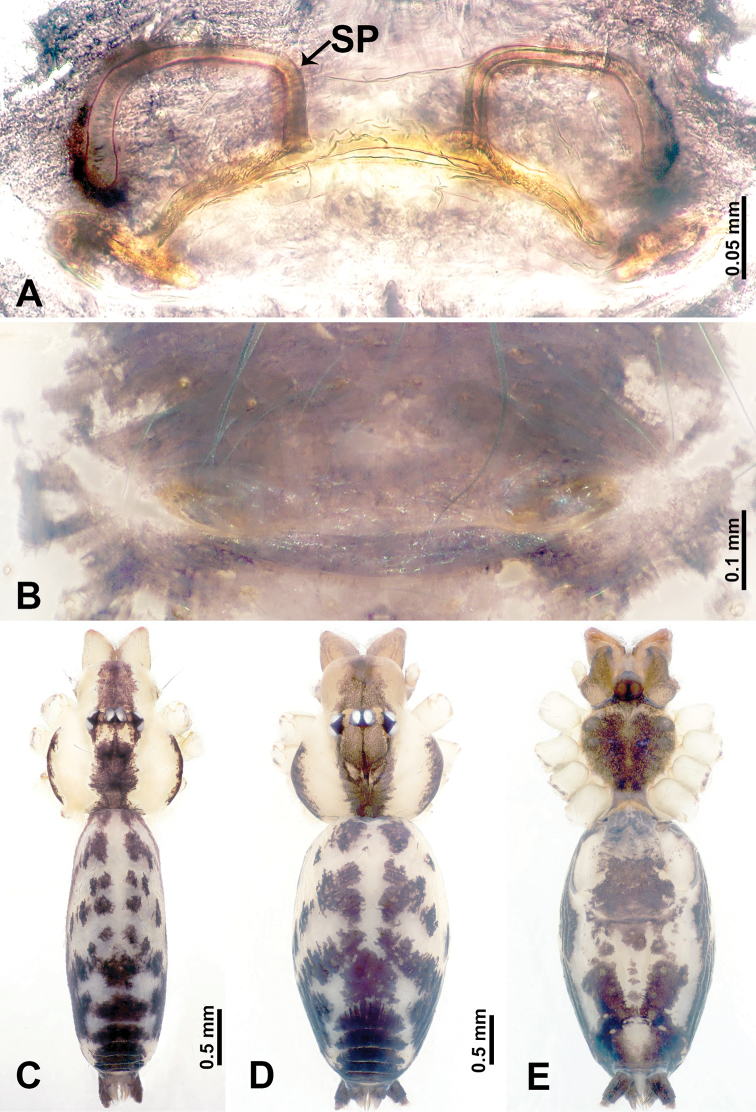
*Leclercera tudao* sp. nov., male holotype and female paratype. **A** Endogyne, dorsal view **B** female epigastric area, ventral view **C** male habitus, dorsal view **D** female habitus, dorsal view **E** female habitus, ventral view. Abbreviation: SP = spermatheca.

**Figure 50.**
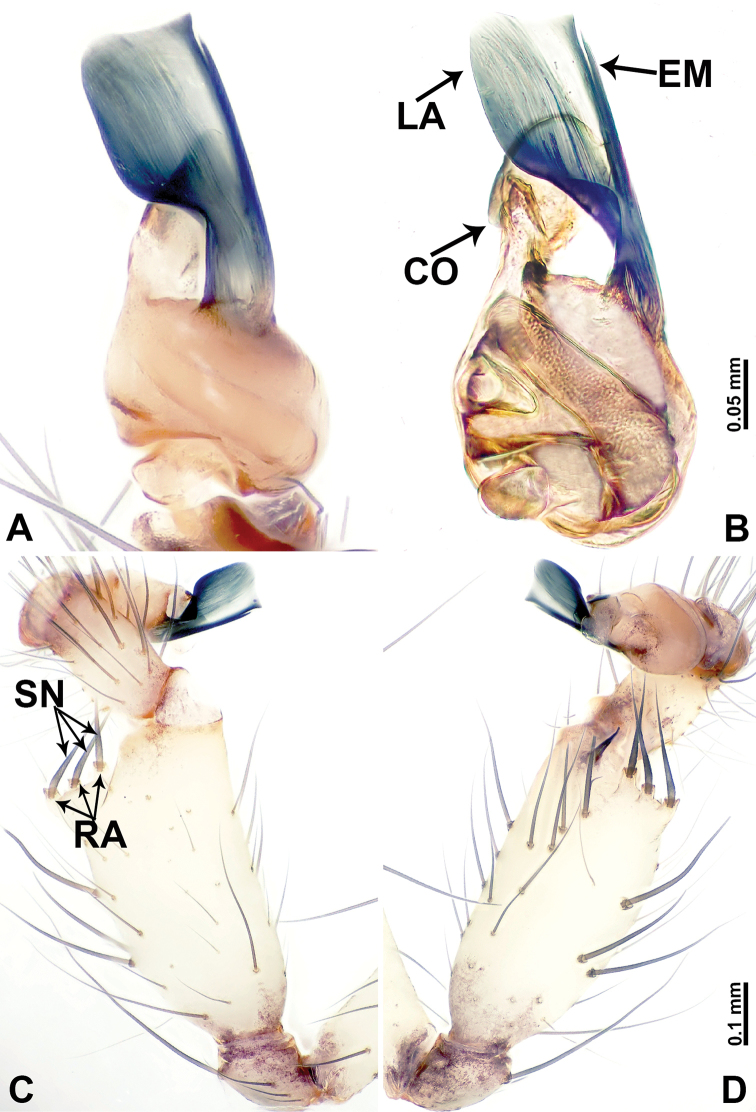
*Leclercera tudao* sp. nov. **A** Palp, ventral view **B** bulb, ventral view **C** palp, prolateral view **D** palp, retrolateral view. Abbreviations: CO = conductor, EM = embolus, LA = laminar apophysis, RA = retrolateral apophyses, SN = spines.

Figs 49 , 50 , 57E , 58

##### Types.

***Holotype***: ♂ (IZCAS), China, Tibet Autonomous Region, Xigaze, Nyalam County, Zham Town, road to Guomen, 27°28.7100'N, 85°58.6920'E, elevation ca 2333 m, 10.VII.2019, X. Zhang, Z. Bai and J. Liu leg. ***Paratype***: 1♀ (IZCAS), same data as holotype.

##### Etymology.

The species name is a noun in apposition derived from the Chinese pinyin “túdāo” (cleaver) and refers to the structure of the laminar apophysis and embolus together resembling a pair of cleavers (Fig. 50A).

##### Diagnosis.

Diagnostic features of the males are discussed in the diagnosis of *L. renqinensis* sp. nov.

##### Description.

**Male** (Holotype). Total length 3.13; carapace 1.00 long, 1.10 wide; abdomen 2.13 long, 0.90 wide. Carapace round and pale brown, with three longitudinal dark brown bands, median band five times wider than lateral bands (Fig. 49C). Chelicerae brown (Fig. 57E). Clypeus pale brown, dark brown medially. Endites dark brown, light brown basally. Labium and sternum dark brown. Abdomen elongated, dorsum with pairs of dark brown spots, posterior with dark brown horizontal stripes, ventrum with dark brown band marginally, antero-ventrally with elliptical dark brown patch, posterior with a pair of distinct rectangular dark brown spots. Legs uniformly brown; measurements: I 20.35 (5.45, 2.00, 5.13, 5.77, 2.00), II 12.95 (3.75, 0.40, 3.60, 3.80, 1.40), III 13.35 (3.75, 0.40, 3.80, 4.00, 1.40), IV 8.74 (2.50, 0.31, 2.34, 2.50, 1.09). Palp (Fig. 50A–D): femur slender, five times longer than patella; patella not swollen; tibia swollen, 1.2 times shorter and 2 times wider than femur, with a distinct spine and three retrolateral apophyses bearing spines anteriorly, spines about three times longer than apophysis (Fig. 50C); cymbium three times shorter than femur; bulb brown, globose, with embolus, laminar apophysis, and conductor arising distally; embolus thin and black, adjacent and basally attached to laminar apophysis; laminar apophysis about 8 times wider than embolus, with flat tip, adjacent to conductor but not attached; conductor with rounded tip, similar to laminar apophysis but two times shorter (Fig. 50B).

**Female** (Paratype). General features and coloration similar to those of male (Fig. 49D, E). Measurements: total length 3.00; carapace 1.00 long, 1.09 wide; abdomen 2.00 long, 1.25 wide. Leg measurements: I – (4.81, 0.40, 4.75, 4.50, -), II – (3.50, 0.40, 3.60, 3.40, -), III 8.60 (2.40, 0.40, 2.40, 2.20, 1.20), IV – (3.85, 0.40, 3.75, 3.50, -). Epigastric area (Fig. 49B): dark brown patch with yellowish and pale brownish slit. Endogyne (Fig. 49A): a pair of stalked spermathecae, stalks curving downwards, almost forming a pair of perpendicular stalks with rounded tips.

##### Distribution.

Known only from the type locality (Fig. 58).

#### Leclercera duibaensis sp. nov.

Taxon classificationAnimaliaAraneaePsilodercidae

9083CD14-23E1-5459-AA30-F98115512A41

http://zoobank.org/00AE7BE3-89B4-47DA-AA5D-BE3A2813C7A7

**Figure 51.**
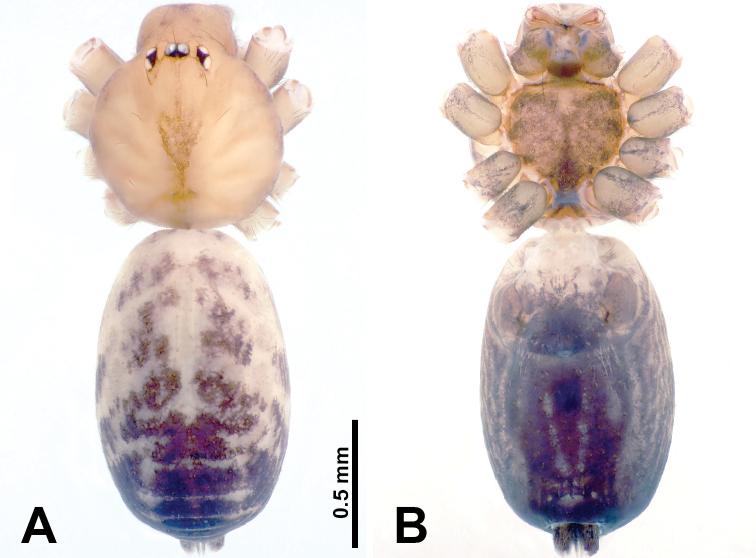
*Leclercera duibaensis* sp. nov., male holotype. **A** Habitus, dorsal view **B** habitus ventral view.

**Figure 52.**
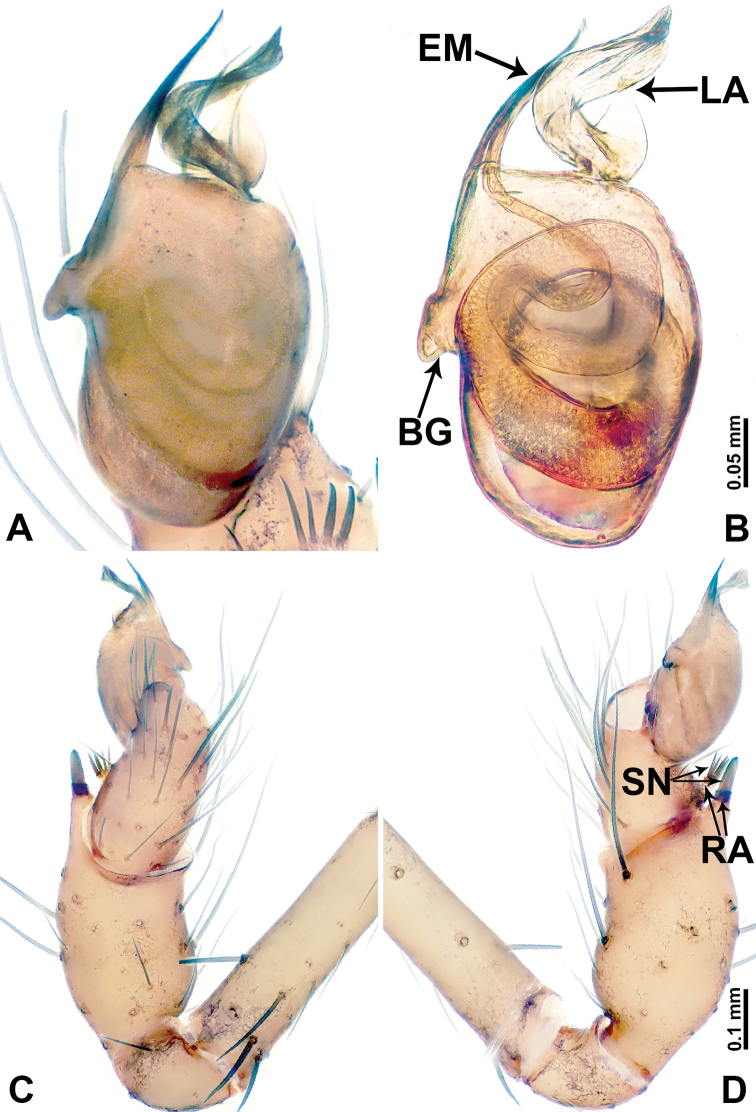
*Leclercera duibaensis* sp. nov. **A** Palp, ventral view **B** bulb, ventral view **C** palp, prolateral view **D** palp, retrolateral view. Abbreviations: BG = bulge, EM = embolus, LA = laminar apophysis, RA = retrolateral apophyses, SN = spines.

Figs 51 , 52 , 57F , 58

##### Types.

***Holotype***: ♂ (IZCAS), China, Tibet Autonomous Region, Shannan, Duopozhang Village, Duiba Village, 29°22.2840'N, 91°41.8320'E, elevation ca 4095 m, 14.VIII.2019, X. Zhang, Z. Bai and J. Liu leg.

##### Etymology.

The species name is an adjective referring to the type locality.

##### Diagnosis.

Diagnostic features of the males are discussed in the diagnosis of *L. shergylaensis* sp. nov.

##### Description.

**Male** (Holotype). Total length 3.60; carapace 1.41 long, 1.40 wide; abdomen 2.19 long, 1.00 wide. Carapace round and brown, with dark brown traces medially (Fig. 51A). Chelicerae brown (Fig. 57F). Clypeus brown. Endites dark brown, light brown basally. Labium dark brown. Sternum dark brown, with light brown “T”-shaped trace anteriorly. Abdomen elongated, antero-dorsally with pairs of dark brown spots laterally, posterior with dark brown horizontal stripes medially, antero-ventrally with pairs of brown, kidney-shaped spots laterally, medially with elliptical patch, posterior with indistinct dark brown vertical patterns. Legs uniformly brown; measurements: I 10.85 (3.85, 0.50, 3.75, 1.75, 1.00), II 10.25 (3.00, 0.50, 3.00, 2.50, 1.25), III 7.39 (2.19, 0.40, 2.00, 1.80, 1.00), IV 12.20 (3.50, 0.50, 4.00, 2.80, 1.40). Palp (Fig. 52A–D): femur slender, three times longer than patella; patella not swollen; tibia swollen, 1.5 times shorter and two times wider than femur, with a retrolateral apophysis bearing a spine anteriorly, spine and apophysis similar in length (Fig. 52D); cymbium two times shorter than femur, with retrolateral apophysis bearing four spines posteriorly, spines and apophysis similar in length (Fig. 52D); bulb brown, ovoid with embolus and laminar apophysis arising distally, presence of a bulge marginally; embolus fine and black, slightly shorter than laminar apophysis; laminar apophysis slightly twisted, forming a “U”-shaped branch, with one branch two times longer than other branch, four times wider than, and not attached to, embolus (Fig. 52B).

**Female**. Unknown.

##### Distribution.

Known only from the type locality (Fig. 58).

#### Leclercera jiazhongensis sp. nov.

Taxon classificationAnimaliaAraneaePsilodercidae

1EE49F61-3F14-564D-B52C-6AE46117DC43

http://zoobank.org/24C9F76A-CC61-4074-82DF-DF241361C705

**Figure 53.**
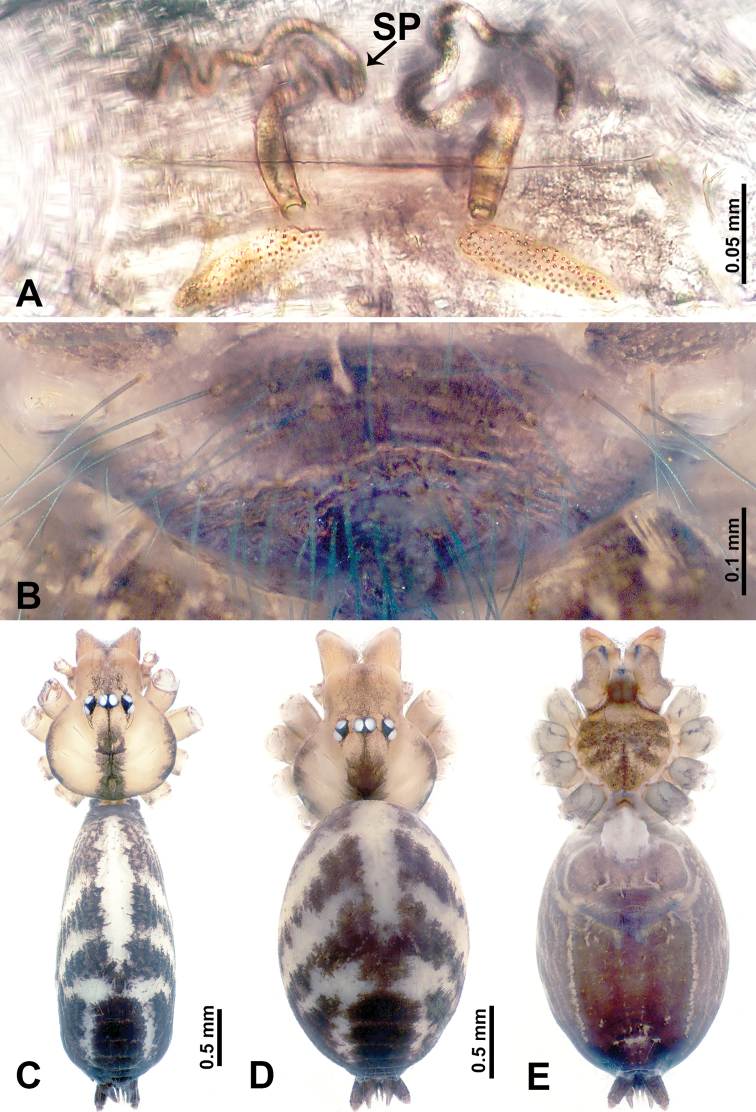
*Leclercera jiazhongensis* sp. nov., male holotype and female paratype. **A** Endogyne, dorsal view **B** female epigastric area, ventral view **C** male habitus, dorsal view **D** female habitus, dorsal view **E** female habitus, ventral view. Abbreviation: SP = spermatheca.

**Figure 54.**
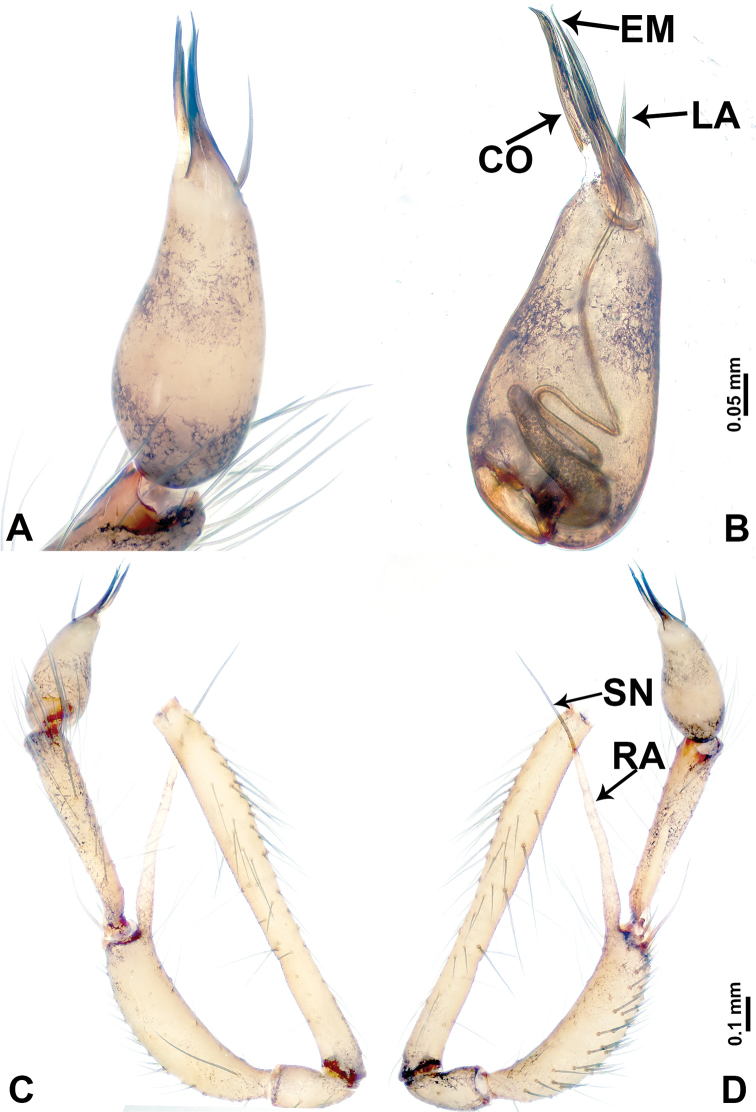
*Leclercera jiazhongensis* sp. nov. **A** Palp, ventral view **B** bulb, ventral view **C** palp, prolateral view **D** palp, retrolateral view. Abbreviations: CO = conductor, EM = embolus, LA = laminar apophysis, RA = retrolateral apophysis, SN = spine.

Figs 53 , 54 , 57G , 58

##### Types.

***Holotype***: ♂ (IZCAS), China, Tibet Autonomous Region, Nyingchi, Bomê County, Yigong Village, mountain in Jiazhong Village, 30°11.2620'N, 94°19.4180'E, elevation ca 3087 m, 18.VII.2019, X. Zhang, Z. Bai and J. Liu leg. ***Paratype***: 1♀ (IZCAS), same data as holotype.

##### Etymology.

The species name is an adjective referring to the type locality.

##### Diagnosis.

Males of *L. jiazhongensis* sp. nov. resemble *L. aniensis* sp. nov. but can be distinguished by the presence of a prolateral apophysis bearing a spine on the tibia (Fig. 54D) (vs. the presence of two retrolateral apophyses, each bearing a spine, on the tibia, where the anterior apophysis is branched and resembles a claw (Fig. 42D)), cymbium similar in length to tibia (vs. cymbium about three times shorter than the tibia), tibia swollen, resembles “D” shape (vs. tibia swollen, resembles isosceles triangle), bulb rather slender and elongated (Fig. 54B) (vs. bulb rather plump and short (Fig. 42B)), and the presence of one laminar apophysis (vs. the presence of three laminar apophyses); females can be distinguished by a pair of long, wavy, stalked spermathecae (Fig. 53A) (vs. a pair of short and upturned stalked spermathecae (Fig. 41A)).

##### Description.

**Male** (Holotype). Total length 3.44; carapace 0.94 long, 1.13 wide; abdomen 2.50 long, 1.13 wide. Carapace round and brown, with three dark brown longitudinal bands, median band four times wider than lateral band (Fig. 53C). Chelicerae brown (Fig. 57G). Clypeus brown, dark brown medially. Endites dark brown, light brown basally. Labium dark brown. Sternum with dark brown stripes. Abdomen elongated, dorsum with three pairs of dark brown spots laterally, posterior with dark brown horizontal stripes, antero-ventrally with a pair of kidney-shaped dark brown spots laterally, followed by elliptical dark brown region, posterior dark brown, delimiting vertical light brown lines laterally. Legs uniformly brown; measurements: I 10.84 (2.24, 0.40, 3.60, 3.00, 1.60), II 10.60 (3.00, 0.40, 3.20, 2.75, 1.25), III 7.49 (2.20, 0.40, 2.00. 1.80, 1.09), IV 10.55 (3.00, 0.40, 3.00, 2.75, 1.40). Palp (Fig. 54A–D): femur slender, five times longer than patella; patella not swollen; tibia swollen forming a “D” shape, 2 times shorter and only slightly wider than femur, with a prolateral apophysis bearing spine anteriorly, spine two times shorter than apophysis (Fig. 54D); cymbium two times shorter than femur, similar in length but two times narrower than tibia; bulb brown, pyriform, with embolus, laminar apophysis, and conductor arise distally; embolus black and slender, widest between laminar apophysis and conductor, similar in length to conductor and two times longer than laminar apophysis; laminar apophysis appears to be shortest, basally connected to embolus; conductor alongside embolus, and similar in width to embolus (Fig. 54B).

**Female** (Paratype). General features and coloration similar to those ofmale (Fig. 53D, E). Measurements: total length 3.00; carapace 1.00 long, 1.00 wide; abdomen 2.00 long, 1.50 wide. Leg measurements: I 10.51 (2.80, 0.31, 3.20, 2.60, 1.60), II 9.35 (2.75, 0.40, 2.75, 2.25, 1.20), III 6.46 (1.75, 0.31, 1.80, 1.60, 1.00), IV 9.32 (2.60, 0.31, 2.66, 2.34, 1.41). Epigastric area (Fig. 53B): semi-circular dark brown patch. Endogyne (Fig. 53A): pair of lengthy, stalked spermathecae with anterior wavy horizontal part and twisted vertical posterior part, both parts with rounded tips, posterior ends with a pair of elliptical bodies.

##### Distribution.

Known only from the type locality (Fig. 58).

**Figure 55.**
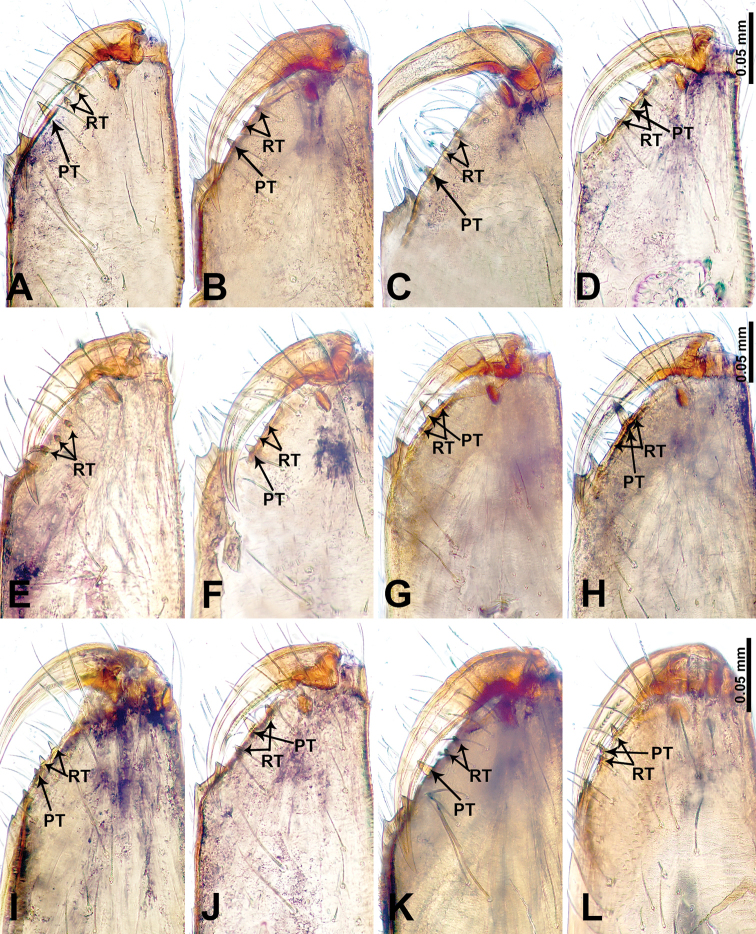
Cheliceral retromargin, posterior view. **A***Leclercera xiaodai* sp. nov. **B***L. zhamensis* sp. nov. **C***L. sanjiao* sp. nov. **D***L. banensis* sp. nov. **E***L. jianzuiyu* sp. nov. **F***L. xiangbabang* sp. nov. **G***L. maochong* sp. nov. **H***L. shanzi* sp. nov. **I***L. yandou* sp. nov. **J***L. suwanensis* sp. nov. **K***L. duandai* sp. nov. **L***L. yanjing* sp. nov. Abbreviations: PT = promargin teeth, RT = retromargin teeth.

**Figure 56.**
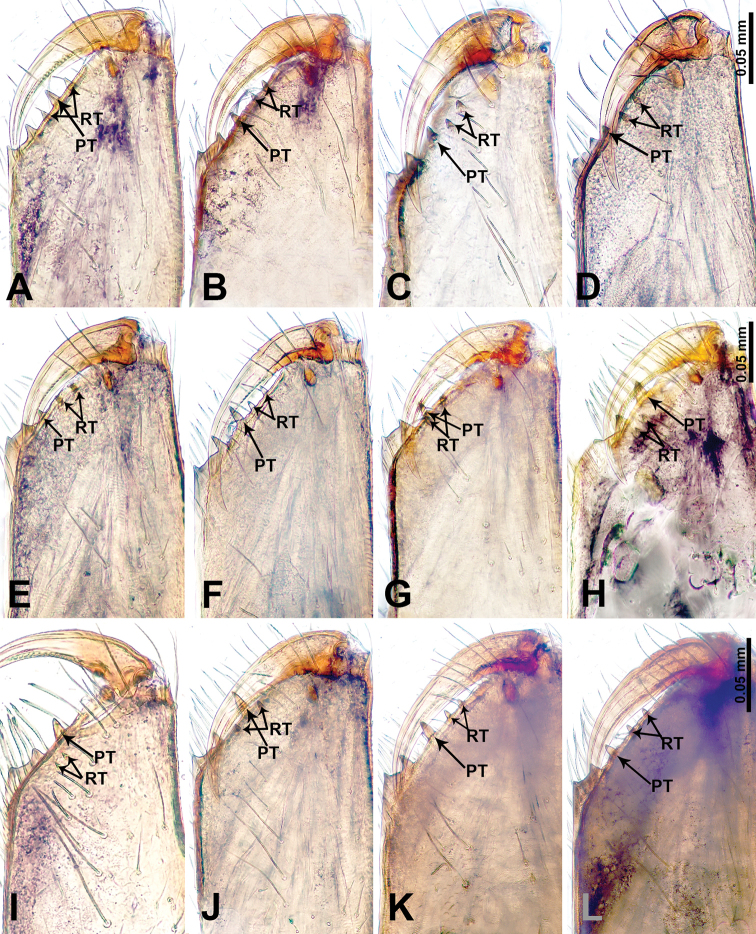
Cheliceral retromargin, posterior view. **A***Leclercera dumuzhou* sp. nov. **B***L. ekteenensis* sp. nov. **C***L. thamkaewensis* sp. nov. **D***L. yamaensis* sp. nov. **E***L. thamsangensis* sp. nov. **F***L. hponensis* sp. nov. **G***L. lizi* sp. nov. **H***L. mianqiu* sp. nov. **I***L. selasihensis* sp. nov. **J***L. yuanzhui* sp. nov. **K***L. paiensis* sp. nov. **L***L. zanggaensis* sp. nov. Abbreviations: PT = promargin teeth, RT = retromargin teeth.

**Figure 57.**
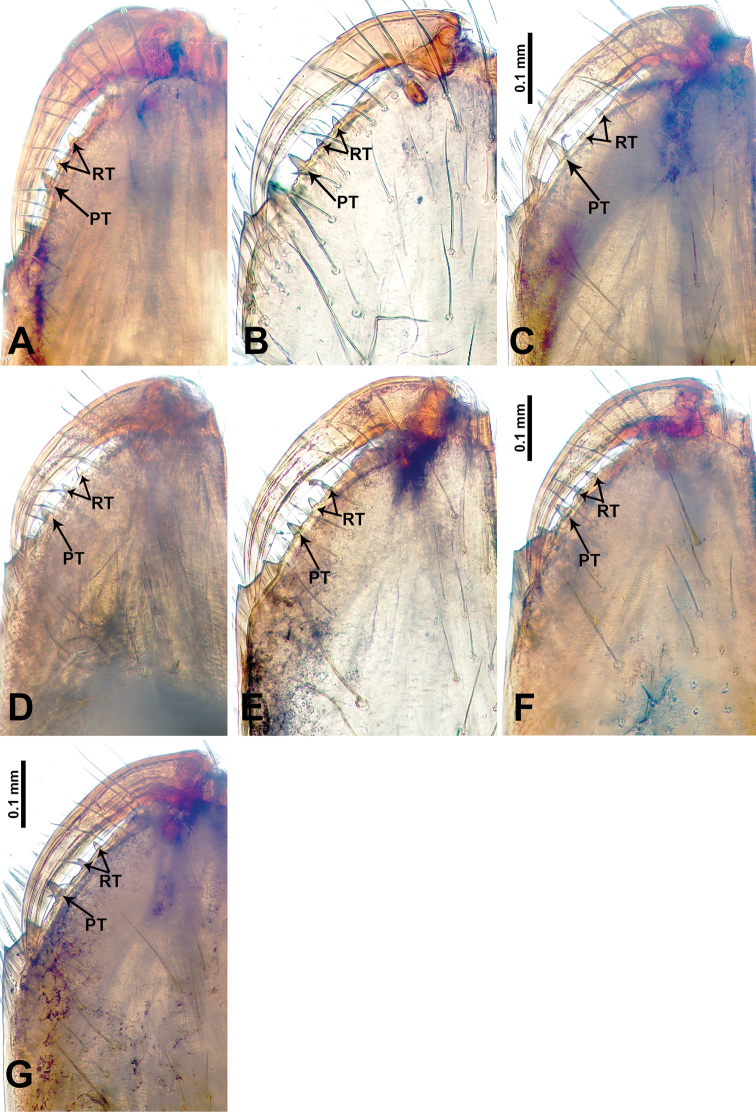
Cheliceral retromargin, posterior view. **A***Leclercera aniensis* sp. nov. **B***L. renqinensis* sp. nov. **C***L. shergylaensis* sp. nov. **D***L. pulongensis* sp. nov. **E***L. tudao* sp. nov. **F***L. duibaensis* sp. nov. **G***L. jiazhongensis* sp. nov. Abbreviations: PT = promargin teeth, RT = retromargin teeth.

**Figure 58.**
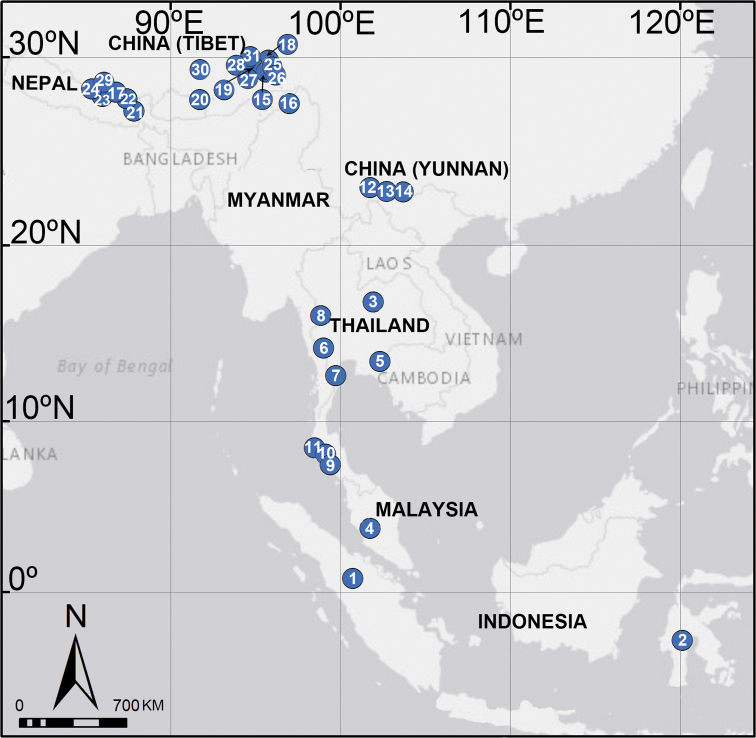
Distribution of new *Leclercera* species in Southeast Asia. **1***L. selasihensis* sp. nov. **2***L. mianqiu* sp. nov. **3***L. thamsangensis* sp. nov. **4***L. yandou* sp. nov. **5***L. thamkaewensis* sp. nov. **6***L. xiangbabang* sp. nov. **7***L. jianzuiyu* sp. nov. **8***L. yamaensis* sp. nov. **9***L. banensis* sp. nov. **10***L. dumuzhou* sp. nov. **11***L. suwanensis* sp. nov. **12***L. maochong* sp. nov. **13***L. yuanzhui* sp. nov. **14***L. shanzi* sp. nov. **15***L. duandai* sp. nov. **16***L. hponensis* sp. nov. **17***L. lizi* sp. nov. **18***L. xiaodai* sp. nov. **19***L. paiensis* sp. nov. **20***L. yanjing* sp. nov. **21***L. ekteenensis* sp. nov. **22***L. zanggaensis* sp. nov. **23***L. zhamensis* sp. nov. **24***L. sanjiao* sp. nov. **25***L. aniensis* sp. nov. **26***L. renqinensis* sp. nov. **27***L. shergylaensis* sp. nov. **28***L. pulongensis* sp. nov. **29***L. tudao* sp. nov. **30***L. duibaensis* sp. nov. **31***L. jiazhongensis* sp. nov.

## Discussion

This study describes 31 new species, yielding a total of 42 species in the genus *Leclercera*. *Leclercera* species are reported for the first time from the Tibet Autonomous Region of China, Malaysia, Indonesia, and Myanmar. The large amount of new species discovered in Tibet (15 new species in this study) are noteworthy. The new Tibetan species occur in close proximity to one another, but this study surprisingly reveals large differences in genital morphology. This is congruent with previous studies of the abundant biodiversity and biological diversification due to the uplift of the Tibetan Plateau (Zhang et al. 2002, Zhao and Li 2017). This study provides a strong case for taxonomic studies in Southeast Asia, especially Tibet. Based on this work and additional observations, we predict that there are additional species in this genus awaiting discovery.

## Supplementary Material

XML Treatment for Leclercera

XML Treatment for Leclercera mianqiu

XML Treatment for Leclercera thamsangensis

XML Treatment for Leclercera yandou

XML Treatment for Leclercera thamkaewensis

XML Treatment for Leclercera xiangbabang

XML Treatment for Leclercera jianzuiyu

XML Treatment for Leclercera yamaensis

XML Treatment for Leclercera banensis

XML Treatment for Leclercera dumuzhou

XML Treatment for Leclercera suwanensis

XML Treatment for Leclercera maochong

XML Treatment for Leclercera shanzi

XML Treatment for Leclercera duandai

XML Treatment for Leclercera hponensis

XML Treatment for Leclercera lizi

XML Treatment for Leclercera xiaodai

XML Treatment for Leclercera yanjing

XML Treatment for Leclercera ekteenensis

XML Treatment for Leclercera zhamensis

XML Treatment for Leclercera sanjiao

XML Treatment for Leclercera selasihensis

XML Treatment for Leclercera yuanzhui

XML Treatment for Leclercera paiensis

XML Treatment for Leclercera zanggaensis

XML Treatment for Leclercera aniensis

XML Treatment for Leclercera renqinensis

XML Treatment for Leclercera shergylaensis

XML Treatment for Leclercera pulongensis

XML Treatment for Leclercera tudao

XML Treatment for Leclercera duibaensis

XML Treatment for Leclercera jiazhongensis
